# High-Throughput Discovery of Ferrocene Mechanophores with Enhanced Reactivity and Network Toughening

**DOI:** 10.1021/acscentsci.5c00707

**Published:** 2025-08-01

**Authors:** Ilia Kevlishvili, Jafer Vakil, David W. Kastner, Xiao Huang, Stephen L. Craig, Heather J. Kulik

**Affiliations:** † Department of Chemical Engineering, 2167 Massachusetts Institute of Technology, Cambridge, Massachusetts 02139, United States; ‡ NSF Center for the Chemistry of Molecularly Optimized Networks, 3065 Duke University, Durham, North Carolina 27708, United States; § Department of Chemistry, 3065 Duke University, Durham, North Carolina 27708, United States; ∥ Department of Biological Engineering, 2167 Massachusetts Institute of Technology, Cambridge, Massachusetts 02139, United States; ⊥ Department of Chemistry, 2167 Massachusetts Institute of Technology, Cambridge, Massachusetts 02139, United States

## Abstract

Mechanophores are molecules that undergo chemical changes in response to mechanical force, offering unique opportunities in chemistry, materials science, and drug delivery. However, many potential mechanophores remain unexplored. For example, ferrocenes are attractive targets as mechanophores due to their combination of high thermal stability and mechanochemical lability. However, the mechanochemical potential of ferrocene derivatives remains dramatically underexplored despite the synthesis of thousands of structurally diverse complexes. Herein, we report the computational, machine learning guided discovery of synthesizable ferrocene mechanophores. We identify over one hundred potential target ferrocene mechanophores with wide-ranging mechanochemical activity and use data-driven computational screening to identify a select number of promising complexes. We highlight design principles to alter their mechanochemical activation, including regio-controlled transition state stabilization through bulky groups and a change in mechanism through noncovalent ligand–ligand interactions. The computational screening is validated experimentally both at the polymer strand level through sonication experiments and at the network level, where a computationally discovered ferrocene mechanophore cross-linker leads to greater than 4-fold enhancement in material tearing energy. This work establishes a generalizable framework for the high-throughput discovery and rational design of mechanophores and offers insights into structure–activity relationships in mechanically responsive materials.

## Graphical abstract

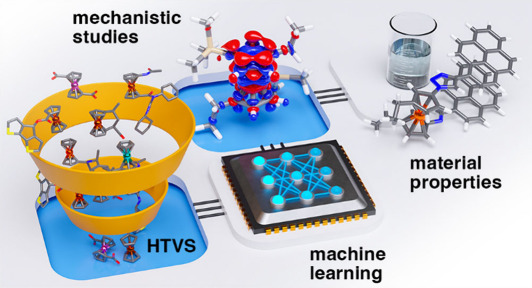

## 1 Introduction

The design and synthesis of mechanophores continues to define contemporary polymer mechanochemistry.^1^ Mechanophores exhibit a wide range of chemical responses to force, including covalent rearrangement,^2^ the release of cargo,^3^ self-strengthening hydrogels,^4–6^ and changed catalytic activity.^7^ Their discovery and study have led to new insights and opportunities in fundamental chemical reactivity,^8^ imaging,^9^ drug delivery.^10^ The most common chemical response to mechanical force, however, remains scission, and the fracture of single polymer chains and of materials made from those chains can be dictated by the reactivity of embedded mechanophores.^11,12^ Notably, depending on network topology, more easily scissile mechanophores do not necessarily lead to more easily fractured materials. Recent advances^12^ have shown that side-chain cross-linked polymer networks incorporating scissile mechanophores can provide materials that are stronger than their nonmechanophore-containing counterparts without any measurable changes to other bulk material properties. A combination of simulations and structure–activity relationships supported a mechanism for toughening in which each preferential scission of a side-chain cross-linker results in an effective increase in the length of the highly tensioned strand, and the longer strand accommodates more stored elastic energy when it ultimately breaks to allow the crack to propagate.^12^ As a result, the network can withstand greater stresses without tearing than it would if the high tension were constrained to only the strand located between the initial cross-linking points. As such, mechanochemistry offers one of several approaches geared toward enhancing the durability of polymer networks,^13–18^ which would address challenges related to the performance and sustainability of polymers^19^ as well as the goal of addressing the plastic waste crisis.^20,21^

However, the identification of new mechanophores is often a trial-and-error process, and experimental techniques to quantitatively study mechanophore kinetics are laborious.^22,23^ Therefore, computational chemistry has been instrumental in advancing our understanding of mechanochemical reaction outcomes,^8,24–26^ reactivity,^27–29^ and mechanisms of scission under tension,^30,31^ in providing insights into dynamic effects,^2,32^ and in developing predictive models for mechanochemical scission.^33–35^ Notably, simulations using the constrained geometries to simulate external force (CoGEF)^36^ approach have found widespread use as a screening tool for mechanophore design.^37,38^

While the design space of possible mechanophores is large, ideal mechanophores for strengthening materials must possess a combination of sufficient thermal stability and mechanochemical reactivity, remaining inert under normal conditions and exhibiting the desired reactivity under tension. Of particular interest for material toughening are metallocenes, a privileged class of mechanophores^39,40^ that are both thermally stable and mechanically labile.^41,42^ Widespread interest in metallocenes, spanning applications in catalysis, energy storage, biological activity, sensing, and more,^43^ has led to the synthesis of diverse derivatives with thousands of unique metallocene structures (Figure 1). Chemical diversity of these complexes includes, but is not limited to, systems with highly sterically crowded substituents such as polysilylated ferrocenes,^44^ extended π-conjugated systems,^45^ heteroatomic substituents on the ligands,^46^ and groups capable of forming intramolecular or intermolecular hydrogen bonds in the ligand backbone.^47^ Furthermore, distinct mechanochemical dissociation mechanisms have been identified that affect mechanochemical reactivity of ferrocene mechanophores. This includes a shearing mechanism where unconstrained cyclopentadienyl (Cp) rings first undergo rotation followed by concerted dissociation through ring slippage or, alternately, a distinct peeling mechanism where distal backbone locking constrains initial ring rotation (Figure 2).

**Figure 1:**
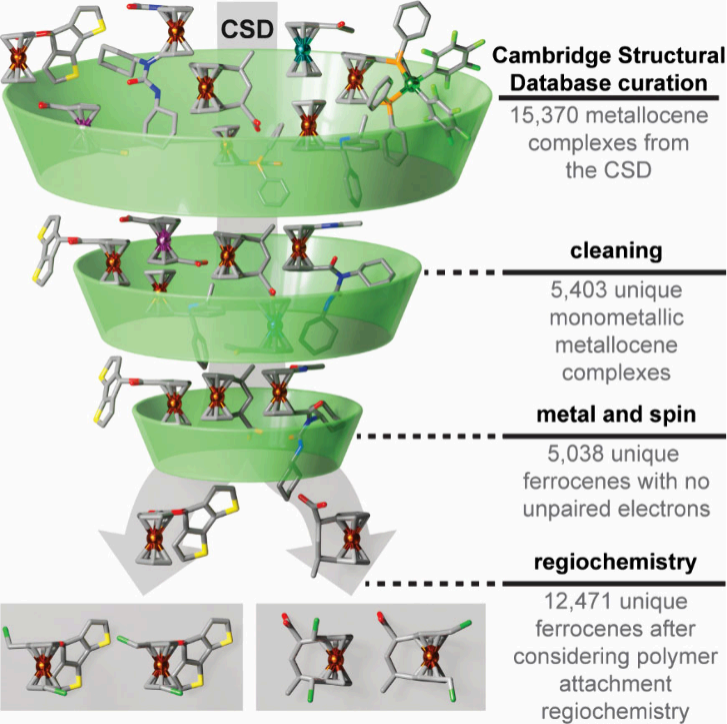
Screening procedure of the CSD curated data set of ferrocene complexes. Randomly sampled complexes are shown as cartoon renderings. Atoms are colored with hydrogen in white, carbon in gray, iron in dark orange, oxygen in red, nitrogen in blue, chlorine in green, and phosphorus in light orange.

**Figure 2:**
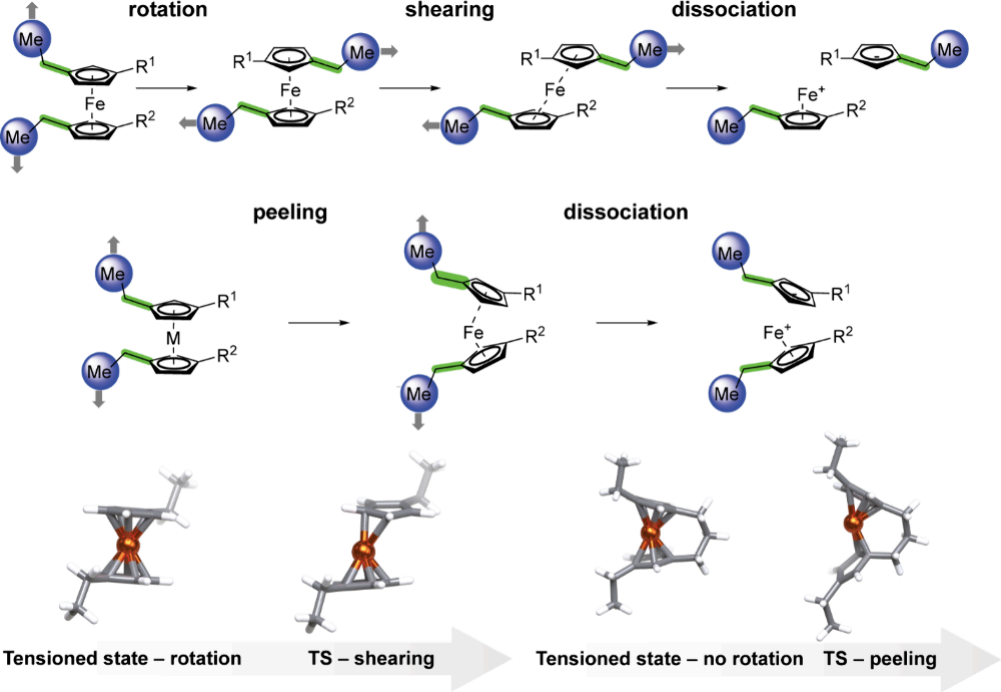
Top: Schematic representation of the shearing dissociation mechanism. Middle: Schematic representation of the peeling dissociation mechanism. Bottom: 3D renderings of two different mechanisms. For the top and middle schematics, blue spheres around the methyl groups indicate two atoms on the introduced ethyl groups that are pulled apart during the calculations. Green highlighted bonds indicate four atoms that define the dihedral angle that distinguishes between the two mechanisms. For the bottom 3D structures, atoms are colored as follows: hydrogen in white, carbon in gray, and iron in dark orange.

Despite the number of unique metallocene structures, only a few have been experimentally studied for their mechanochemical activation.^40–42,48^ A traditional trial-and-error approach to discovering new metallocene mechanophores is unlikely to scratch the surface of this vast chemical space, and a transformative alternative is needed to systematically uncover optimal mechanophores from vast chemical libraries. Moreover, even low-cost computational screening of all known metallocenes presents significant challenges, as this would correspond to tens of thousands of simulations. We therefore propose a machine learning approach based on computationally simulated data to guide the mechanophore screening process, with a goal of identifying synthetically accessible compounds that are more mechanochemically scissile than their predecessors. Further, we demonstrate that our high-throughput computational workflow leads to the discovery of novel compositions of matter with improved toughness and tear resistance as bulk materials, i.e. by incorporating the computationally discovered mechanophores as cross-linkers in rubbery polymer networks.

In the rest of this paper, we describe our machine learning-accelerated screening and analysis of a vast space of synthetically accessible ferrocene derivative mechanophores. We use this set to identify strategies for modulating the force responsiveness of ferrocene derivatives. We then describe how we validate a promising, surprisingly low-force lead compound using both single-strand sonication and polymer network tearing experiments. This integrated screening strategy represents a scalable model for computationally guided discovery of functional molecular units in polymer science.

## 2 Results and Discussion

### 2.1 Machine Learning Accelerated Discovery of Ferrocene Mechanophores

We curated a data set of candidate mechanophores from the Cambridge Structural Database (CSD) to understand the diversity of synthetically accessible metallocenes suitable for mechanochemical activation (Figure 1 and Supporting Information Text S1). Most linear metallocenes contained ferrocene or ferrocenium cores, motivating our decision to limit our investigation to iron complexes (Supporting Information Figure S1). The final calculation-ready set consists of 5,038 unique functionalized ferrocene complexes, and consideration of potential regioisomers for polymer integration expands the data set to 12,471 unique structures (Figure 1). We investigated mechanical responsiveness by carrying out low-cost constrained geometries simulate external force (CoGEF) simulations^36^ for a subset of curated ferrocene complexes. We randomly sampled and characterized roughly 5% of the total set (i.e., 425 functionalized complexes arising from 381 unique synthesized complexes) with CoGEF using density functional theory (DFT) to calculate an initial set of maximum force (*F*_max_) values for generating structure–force relationships. The simulated *F*_max_ values in the randomly selected data set range from ∼2.1 nN to 4.4 nN, with most of the complexes exhibiting an *F*_max_ of around 3.8 nN (Figure 3a). This cluster around 3.8 nN corresponds to complexes that largely behave like unsubstituted ferrocene, with the functionalizations only weakly perturbing the intrinsic reactivity of ferrocene. While a small number of unbridged ferrocenes, i.e., ferrocenes that do not have covalent cross-linkers between two Cp rings, are significantly more labile than the median, the vast majority of more reactive ferrocenes are bridged ansa-ferrocenes containing a covalent cross-linker between two Cp rings (Figure 3a). A range of mechanochemical activity with varying cross-linker identity and relative stereochemistry of polymer attachment has been observed for bridged ferrocenes,^42^ but the diverse reactivity of unbridged ferrocenes has not yet been studied. Therefore, we focused our search on identifying unbridged ferrocenes with increased mechanochemical reactivity. Despite the high *F*_max_ around 3.8 nN for most complexes, encouragingly, 11% of unbridged ferrocenes are predicted to be significantly more reactive with *F*_max_ below 3.5 nN, while also covering a wide range of reactivity (Figure 3a). However, given that the majority of complexes have relatively low reactivity (i.e., high *F*_max_), the identification of more reactive species through random screening is inefficient and requires an alternative approach.

**Figure 3:**
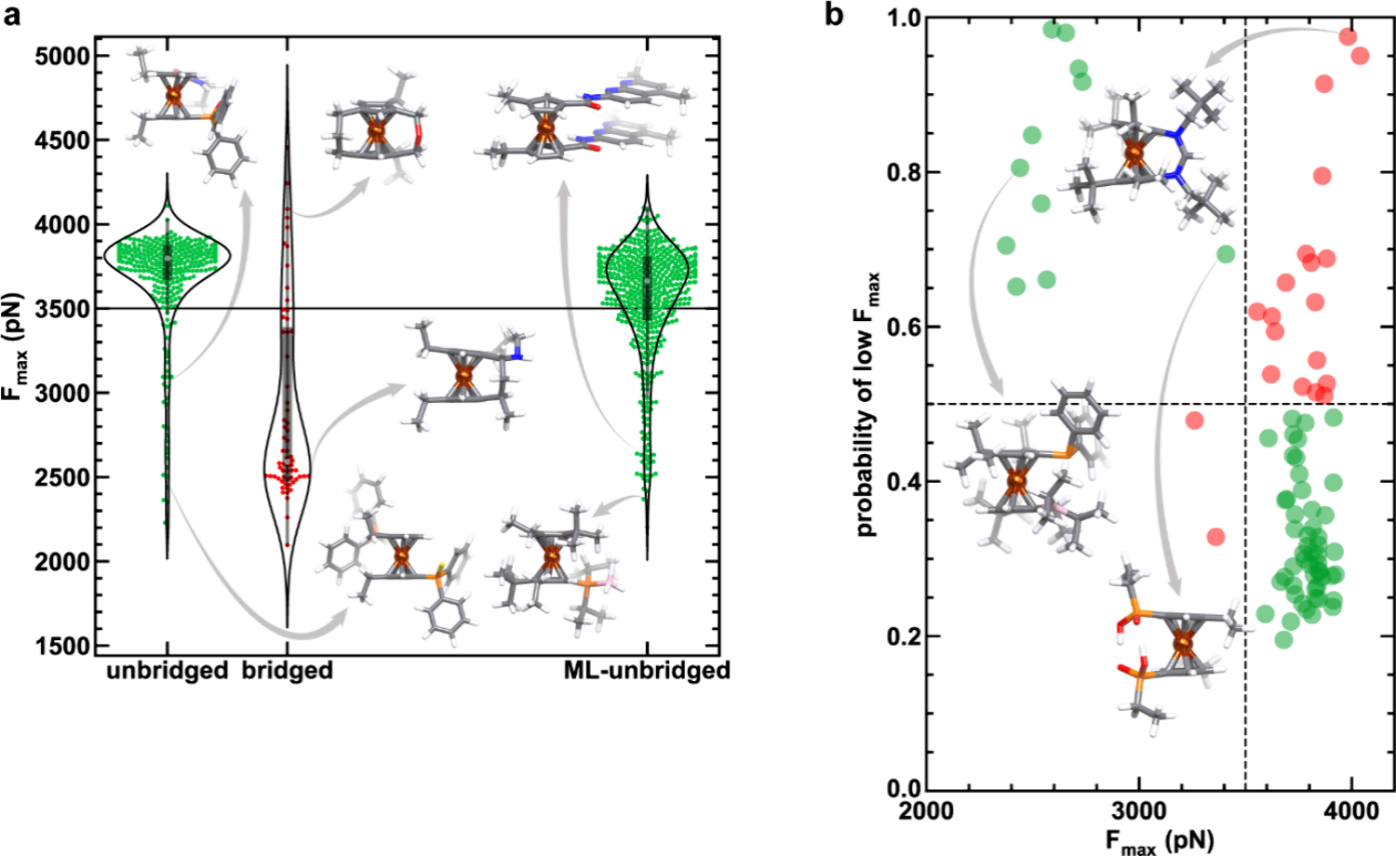
Machine learning guided discovery of ferrocene mechanophores. **a**, The maximum force distribution (violin plots) of the randomly selected unbridged (unbridged, green), randomly selected bridged (bridged, red) and machine learning selected (ML-unbridged, green) ferrocenes. The violin plots are shown with the density, the median (white marker), and the interquartile range (box) displayed. Structures from all sets with varying degrees of mechanochemical reactivity are displayed as insets. **b**, Classifier probability as a function of *F*_max_. Decision boundaries are shown as dashed lines. Correctly classified complexes are colored in green and incorrectly classified complexes are colored in red. Examples of true positive and false positive complexes are shown as insets. All structures are colored as follows: hydrogen in white, carbon in gray, nitrogen in blue, oxygen in red, phosphorus in orange, boron in pink, and iron in dark orange.

To leverage the information from our initial screen while biasing the choice of complexes for CoGEF simulation toward those with lower *F*_max_ values, we trained machine learning (ML) classification models that can predict whether a complex will have high reactivity (i.e., low *F*_max_), increasing the likelihood of high mechanochemical lability. Given a limited understanding of mechanochemical reactivity of ferrocenes and the need for a low-cost ML model representation for featurization, we used revised autocorrelations (RACs).^49^ The RAC features are sums of products and differences of five atom-wise properties (topology, identity, electronegativity, covalent radius and nuclear charge) on the molecular graph that have shown good performance for transition metal complex property predictions (see Computational Details and Supporting Information Text S2).^49–51^ We trained an artificial neural network (ANN) classifier in order to accelerate the screening of our entire data set to identify additional ferrocenes with high reactivity (Supporting Information Table S5 and Section 4.3). We chose an ANN because it offered better performance for classification, including much better identification of reactive complexes on the set aside test set in comparison to alternatives (Supporting Information Table S1). As a classification model, the output produced is a continuous value between 0 and 1 representing the probability of a complex having an *F*_max_ below 3.5 nN (Supporting Information Text S3 and Table S1).

Our ANN classification model demonstrates promising performance on a set-aside 20% test set, identifying the majority (85%) of more reactive complexes accurately (Figure 3b and Supporting Information Figure S2 and Table S1). Therefore, we used the classifier to identify ferrocene complexes in the full data set that were predicted to have low *F*_max_. Consistent with the randomly selected subset, a majority of the structures are identified as less reactive (i.e., higher *F*_max_) by the classifier (Supporting Information Figure S3). After introducing additional constraints, including a cutoff to isolate high-probability predictions (i.e., classifier probability >0.7) and limiting the complex size (i.e., the number of atoms) for simulation efficiency, we identified 556 promising unbridged complexes for follow-up calculation with CoGEF (Supporting Information Text S4). Our DFT-computed *F*_max_ values over this set indicate a significant improvement in the reactivity of these complexes (Figure 3a). After incorporating this data and retraining an ML classifier, we found that the space of reactive ferrocenes was nearly exhausted because this retrained model identified less than 100 additional reactive complexes (Supporting Information Figures S4–S6). Thus, we did not continue to explore the space for additional leads with DFT and instead analyzed properties of the resulting complexes.

### 2.2 Design Principles of Ferrocene Mechanophores

We explored the geometric and electronic factors that could explain the behavior of unbridged ferrocenes identified by our ML models to better understand the underlying design principles for promoting mechanochemical activation of ferrocene complexes. Among the experimentally studied ferrocene mechanophores, bridged- or ansa-ferrocenes have shown the highest reactivity, and a lower dihedral angle between attachment points was shown to lower activation energies for ansa-ferrocenes.^42^ However, we expected that because Cp ring rotation in unbridged ferrocenes is highly labile with a relatively low barrier,^52^ this dihedral angle in the ground state should not necessarily alter ferrocene activation. Nevertheless, based on CoGEF simulations and DFT-optimized structures, we indeed found that the increased attachment point overlap (i.e., 1/abs­(dihedral)), improves mechanochemical reactivity for unbridged ferrocenes (Figure 4a and Supporting Information Figure S7). Further analysis of more reactive complexes with low dihedral angles between attachment points reveals that these complexes undergo ligand dissociation through a peeling mechanism, much like bridged ferrocenes, whereas most other unbridged ferrocenes undergo a shearing dissociation (Figure 2 and Supporting Information Figure S8). Furthermore, complexes with low *F*_max_ and low dihedral angles also largely have noncovalent interactions between two coordinating Cp ligands, such as hydrogen bonding and π–π stacking interactions (Figure 4a). Therefore, we postulate that favorable noncovalent interactions between two ligands could lead to conformational locking, similar to that caused by the covalent linkages in bridged complexes that change the ligand dissociation mechanism and improve reactivity. This hypothesis is further supported by estimation of ligand–ligand interaction energies and corresponding energy decomposition analysis (Supporting Information Text S5 and Figures S9–S13).

**Figure 4:**
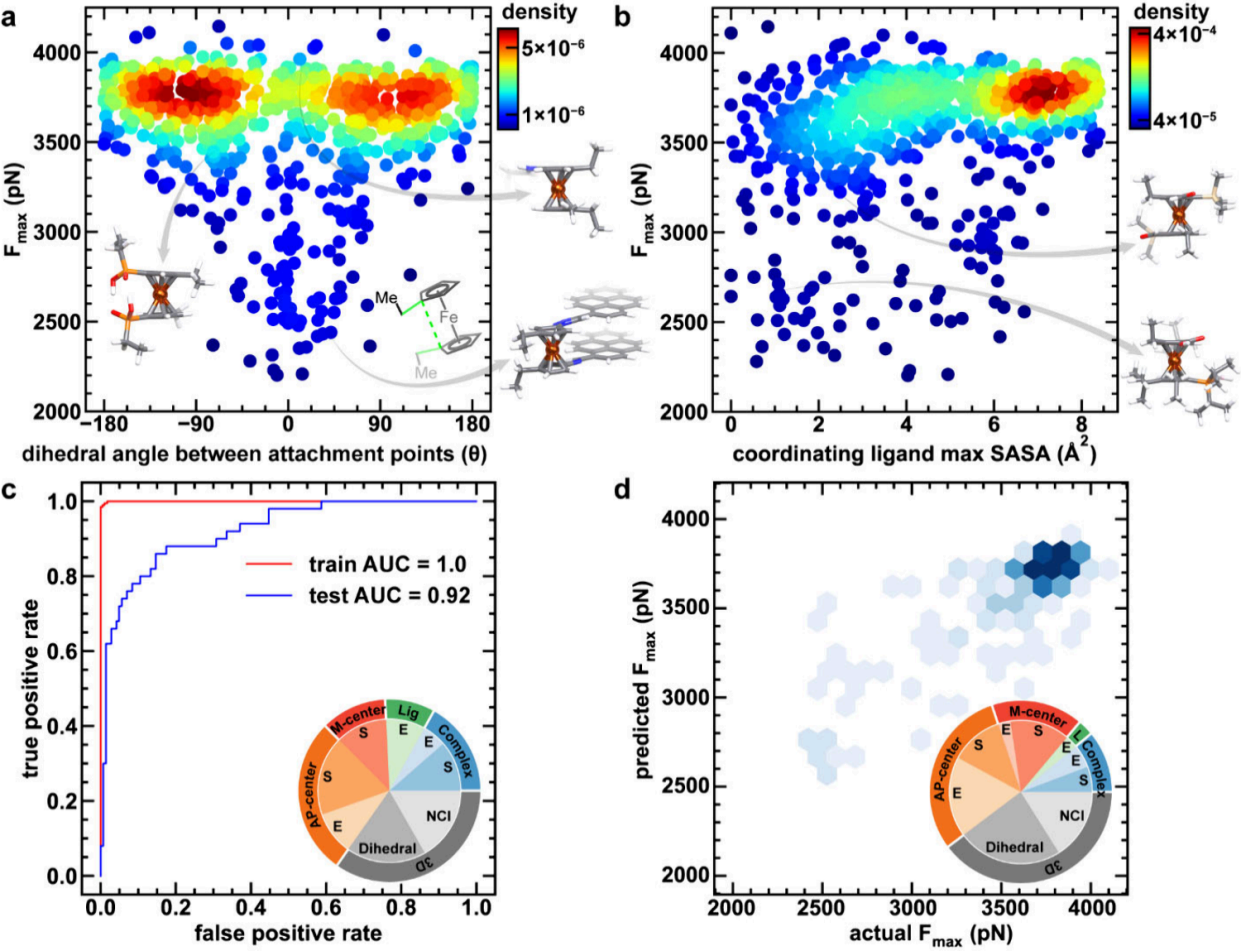
Factors promoting mechanochemical reactivity of ferrocenes. CoGEF simulated *F*_max_ values as a function of **a**, dihedral angle between attachment points and **b**, coordinating ligand maximum solvent accessible surface area. **c**, ROC-AUC curve for classification and **d**, predicted vs actual *F*_max_ values for the regression task. The relevant dihedral angle is highlighted in light green in the inset of **a**. Data points are colored by kernel density estimation (KDE) density values, as indicated by inset color bars for **a** and **b**. Example structures are shown as insets. Atoms are colored as follows: iron in dark orange, carbon in gray, hydrogen in white, oxygen in red, nitrogen in blue, boron in pink, silicon in beige. Feature importance analyses for each model are shown as insets. Training sets are in red, and test sets are in blue for c. Data points for regression in **d** are shown as a hex plot with only the test set shown, and points of higher density are indicated in darker blue colors. In the inset pie charts for **c** and **d**, Lig (L) refers to ligand-based RAC features, M-centered refers to metal-centered RAC features,AP-centered refers to attachment point-centered RAC features,and complex refers to full complex-based RACs features. Geometric features (i.e., topology, covalent radius, and identity) are indicated by the letter S and electronic features (i.e., electronegativity and atomic number) are indicated by the letter E.

To extend our investigation to the environment immediately around the metal, we computed the atom-localized solvent-accessible surface area^53^ (SASA) of the metal-coordinating atoms of the two Cp rings. The maximum SASA between the two ligands, i.e., the SASA of the less sterically crowded ligand, positively correlates with the computed *F*_max_ values (Figure 4b). Given that the lower SASA indicates lower solvent-accessible surface area for the Cp ring, a low maximum SASA computed over both ligands suggests that both ligands are sterically congested. While this descriptor does not have a high correlation to *F*_max_, we observe an increased occurrence low activation forces when this descriptor is small. This suggests that increased steric crowding near both Cp rings (i.e., a decrease in maximum SASA value) improves mechanochemical reactivity. Furthermore, we observe that the Mulliken partial charge on the iron center in the ground state calculated from the DFT optimized geometry is also moderately positively correlated with the *F*_max_ value, highlighting that a more negative charge on the metal center can lead to more labile ligand dissociation (Supporting Information Figure S14). This observation can be attributed to the heterolytic nature of the Cp ligand dissociation,^41^ which would be more facile if the metal is less highly charged in the ground state. Other electronic and steric descriptors that relate to both metal-local, and more global steric and electronic trends, such as Sterimol parameters,^54^ show weaker correlation with observed mechanochemical activity (Supporting Information Figures S15–S23, Text S5, and Table S2). These findings underscore the complex, multidimensional factors governing mechanochemical activation in ferrocenes—a picture that resists reduction to simple heuristics and instead benefits from data-driven exploration.

We thus trained new random forest regression and classification models to develop a more comprehensive understanding of ferrocene complex reactivity under force and to establish design principles for mechanochemical reactivity by interpreting their learned feature importances. Random forest models provide a highly interpretable framework for developing heuristic guidelines because they provide a transparent weighting of the features that impact the resulting models. We incorporated two additional 3D features, which we expected to affect reactivity but could not be easily captured with RACs, the dihedral angle of the DFT-optimized ground state structure and the DFT-computed ligand–ligand interaction energy. Other features such as the SASA or Sterimol parameters can be expected to be captured by the RACs that describe the size and bulk of the molecule and were not added to the feature set. To enhance model interpretability, we conducted feature selection, resulting in a reduced set of 14 features for classification and 22 features for regression models (Supporting Information Text S7 and Tables S3–S4). The final models show promising performance, with improved class separation for the classification model over our original ANN and a mean absolute error of 0.15 nN for the regression model (Figure 4c,d and Supporting Information Figure S24).

Feature importance analysis confirms the high relevance of the newly introduced 3D features, which account for approximately one-third of the total feature importance in both models (Figure 4). Additionally, metal-local features, both due to RACs centered around the metal itself (M-center) and due to RACs around the coordinating atom (AP-center) where the force is applied, account for 40–50% of the total model feature importance (Figure 4). The significant contribution of AP-centered features emphasizes the importance of regiochemistry and can be attributed to the directional nature of mechanical force as a stimulus (Figure 4). A notable difference between the two models’ feature importance scores is the relative significance of electronic features, which become more crucial for the regression task. This finding suggests that while sterically large substituents impact *F*_max_, altering the electronic character leads to subtler changes, allowing for fine-tuning of the mechanochemical response. It is also noteworthy that while the two models have distinct features, the regression model can still correctly classify 76% of the complexes if treated as a classification model with a 3.5 nN decision boundary. Together, these models highlight the importance of backbone locking through noncovalent interactions (NCI) and sterically large substituents with an emphasis on regiochemistry for altering the mechanochemistry of ferrocenes.

### 2.3 Mechanistic Insights from Force-Modified Potential Energy Surfaces

While large-scale screening provides general guiding principles for mechanochemical understanding, a detailed mechanistic study provides deeper insights into factors affecting reactivity. Therefore, we selected eight complexes to simulate their mechanochemical activation using the external force is explicitly included (EFEI) method to gain insights into how mechanochemical activation is promoted and to validate high-throughput CoGEF results. EFEI calculations can be used to build force-modified potential energy surfaces and provide additional mechanistic insights into factors governing reactivity. We selected these eight complexes for their wide-ranging CoGEF-calculated *F*_max_ values from 2.3 nN to 3.7 nN as well as their distinct chemical features such as steric crowding and ligand–ligand NCIs. Because of our intention to test the results of the computations in experiments, we also evaluated the synthetic accessibility for polymer mechanochemistry as follows. First, our standard experimental method for attaching cross-linking groups to ferrocene derivatives involves lithiation of the Cp rings, and so we prioritized compounds with functional groups that were likely to be compatible with that methodology. Second, we made an initial appraisal of the likely synthetic effort that would be required and prioritized compounds that we perceived to require less intensive synthesis. No additional attempt to judge the likely mechanochemical reactivity of the candidate complexes was made or considered. More specifically, the eight EFEI-characterized complexes included three previously experimentally studied ferrocene mechanophores (unsubstituted ferrocene, *cis*-ansa ferrocene and *trans*-ansa ferrocene, i.e., the experimental set); a noncovalent interaction (NCI) set consisting of a complex with a larger dihedral value between attachment points and the presence of a strong H-bond NCI (CSD refcode: **EGIPER**), a complex with relatively weaker extended π-stacking interaction and a small dihedral angle (CSD refcode: **EFILAI**) and a complex with weak H-bonding interactions and a small dihedral angle (CSD refcode: **BADXOU**); and a steric set consisting of a complex with a bulky trimethylsilyl (TMS) group and an aldehyde substituent (CSD refcode: **NUSZEG**) as well as a simpler complex that only contained a single TMS substituent on each Cp ring (m-TMS) to isolate the steric effects from the TMS group. The simplification in the lattermost case was motivated to increase synthetic accessibility.

Modeling of the experimental set shows good qualitative agreement between experimental and calculated reactivity, successfully capturing the relative barriers under applied force for the three species as measured by single-molecule force spectroscopy in prior work^9^ (Supporting Information Figure S25). This experimental set highlights that a smaller dihedral angle between the two atoms where the force is applied in backbone-locked ferrocenes leads to improved mechanochemical coupling (i.e, a steeper slope in the linear fit shown in Supporting Information Figure S25). Small dihedral angles are also associated with reactivity in the NCI set. For the **EFILAI** and **EGIPER** complexes with relatively strong ligand–ligand interactions, through-space interactions are maintained under external force until the transition state associated with dissociation (Figure 5a and Supporting Information Figures S26–S27). Consistent with observations on ansa-ferrocenes, dihedral angle alignment leads to an improved mechanochemical coupling for this NCI set. Analysis of the ground state structures under force of **EFILAI**, **EGIPER**, and unsubstituted ferrocene complexes reveals that better spatial alignment of the force vector with the handles coupled with the rotational locking leads to increased distortion of the complex along the reaction pathway, promoting reactivity (Figure 5b). That is, the attachment points (APs, i.e., ethyl groups) are nearly aligned in **EFILAI**, like *cis*-ansa Fc, resulting in a strong mechanochemical coupling due to backbone NCI locking hindering rotation. On the other hand, in **EGIPER** APs are more staggered, like *trans*-ansa Fc, resulting in a moderate mechanochemical coupling, which is still stronger than unsubstituted Fc. However, when ligand–ligand interaction is weak, as is the case for the complex **BADXOU**, hydrogen bonding is easily disrupted at forces above 1.0 nN external force, resulting in a lower activation energy at 1.00 nN external force. Consequently, the **BADXOU** complex behaves like unsubstituted ferrocene at higher forces (Supporting Information Figure S28).

**Figure 5:**
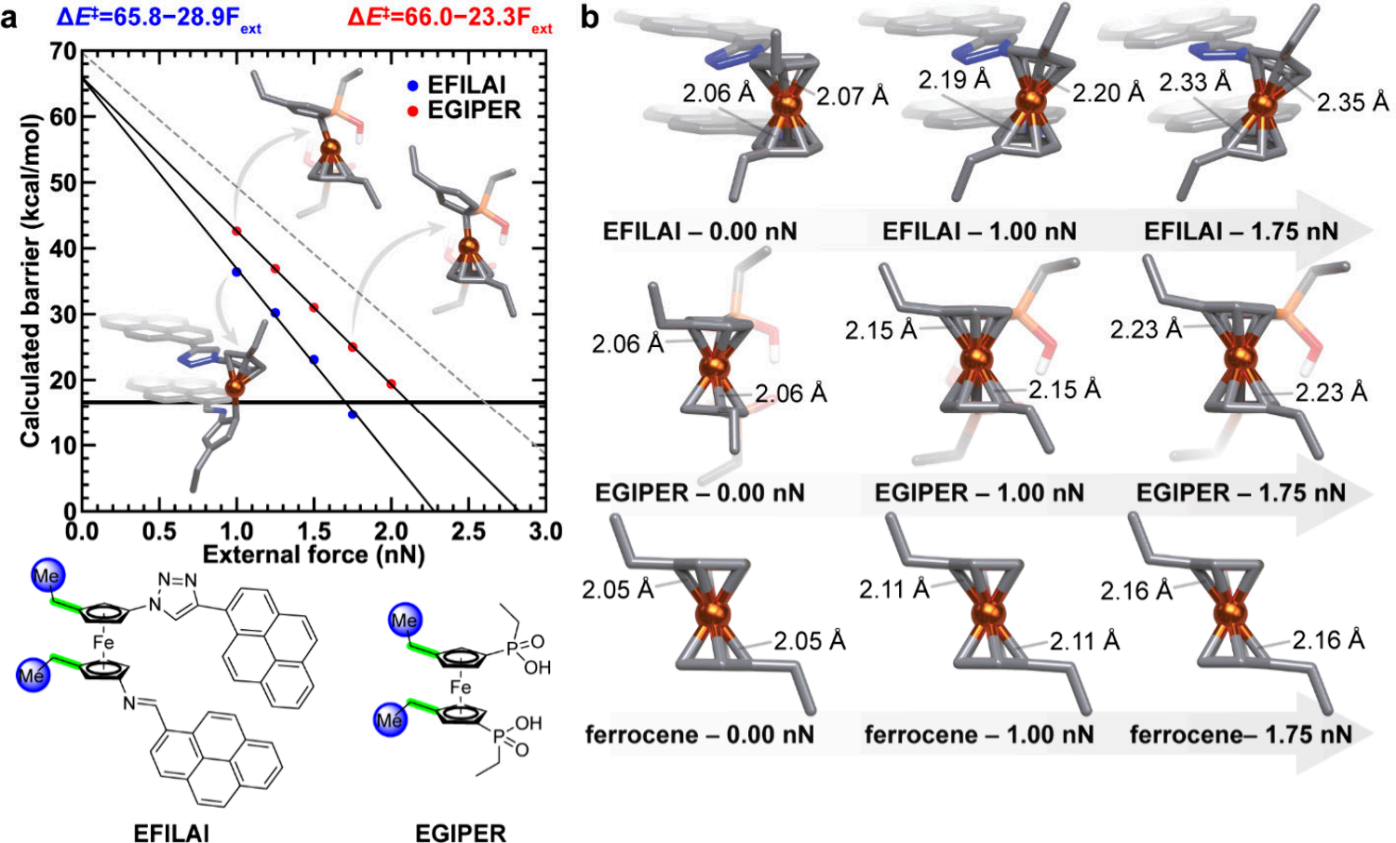
Force-modified ferrocene ligand dissociation of NCI complexes with noncovalent interactions and π-stacking. **a**, Calculated ligand dissociation barriers of **EFILAI** and **EGIPER** complexes as a function of force; **b**, ground state structures of **EFILAI**, **EGIPER**, and unsubstituted ferrocene at different applied external forces. Linear fits for each curve are shown as black lines and their equations are shown at top. For reference, the linear fit of unsubstituted ferrocene is shown as the dashed gray line. The threshold energy of 16.6 kcal/mol that can be used to estimate activation forces measured by single molecule force spectroscopy is shown as a bolded black horizontal line. Transition state structures are shown as insets. H atoms are omitted. For insets, atoms are colored as follows: C in gray, N in blue, Fe in orange, P in light orange, O in red. Schematic representations of the two substituted complexes studied here are shown in the bottom left corner. Atoms that had forces applied to them are highlighted as blue spheres. The relevant dihedral angle is highlighted in light green on both schematic representations.

On the other hand, in the steric set, the **NUSZEG** complex has reduced activation energies for force-induced rupture due to the increased inherent reactivity toward dissociation, as quantified by a y-intercept of the linear fit that is 13.7 kcal/mol lower than that for unsubstituted ferrocene (Figure 6a). While the linearity of force-dependent activation energies can deviate at very low and very high forces, the intercept still provides valuable qualitative insights on relative thermal activation energies. Similarly, the extrapolated force-free activation energy of m-TMS ferrocene is also substantially (i.e., 8 kcal/mol) lower than that of unsubstituted ferrocene (Figure 6a). We computed the electron density differences between the whole complex and that for the separate density obtained on the dissociating ligand and the remaining complex both in the ground state and in the transition state. This analysis reveals that, in the transition state, TMS groups interact with the metal center, leading to the delocalization of the C–Si bond density toward iron, an interaction absent in the ground state (Figure 6b). These findings are further supported by energy decomposition analysis^55^ (i.e., ALMO-EDA), which reveals that the most favorable changes in interaction energies, when compared to unsubstituted ferrocene, result from increased intramolecular polarization. This leads to stronger electrostatic interactions rather than improved strain relief through either lower distortion or lower Pauli repulsion in the transition state (Supporting Information Table S5). This method of transition state stabilization was unanticipated by us and plays a crucial role in the reactivity predicted for TMS-substituted ferrocenes. To test the hypothesis of transition state stabilization and disentangle effects from the ground state destabilization due to steric crowding, we also analyzed a TMS-substituted ferrocene where the polymer handles are substituted ortho to TMS groups (o-TMS-Fc). We hypothesized that due to force vector alignment, stabilizing interactions between the TMS group and the metal center would be absent in this complex in the transition state, leading to higher dissociation barriers. Indeed, TMS group interaction with the metal center is absent both in the ground state and the transition state for o-TMS-Fc, leading to significant reduction in reactivity, and the force-modified reactivity of o-TMS-Fc strongly resembles that of unsubstituted ferrocene (Figure 6). This analysis is again consistent with the findings from ALMO-EDA (Supporting Information Table S5). This finding reveals the significance of transition state stabilization in altering mechanochemical reactivity and the importance of the directionality of applied forces, consistent with our feature importance analysis of random forest models assigning high importance to AP-centered features.

**Figure 6:**
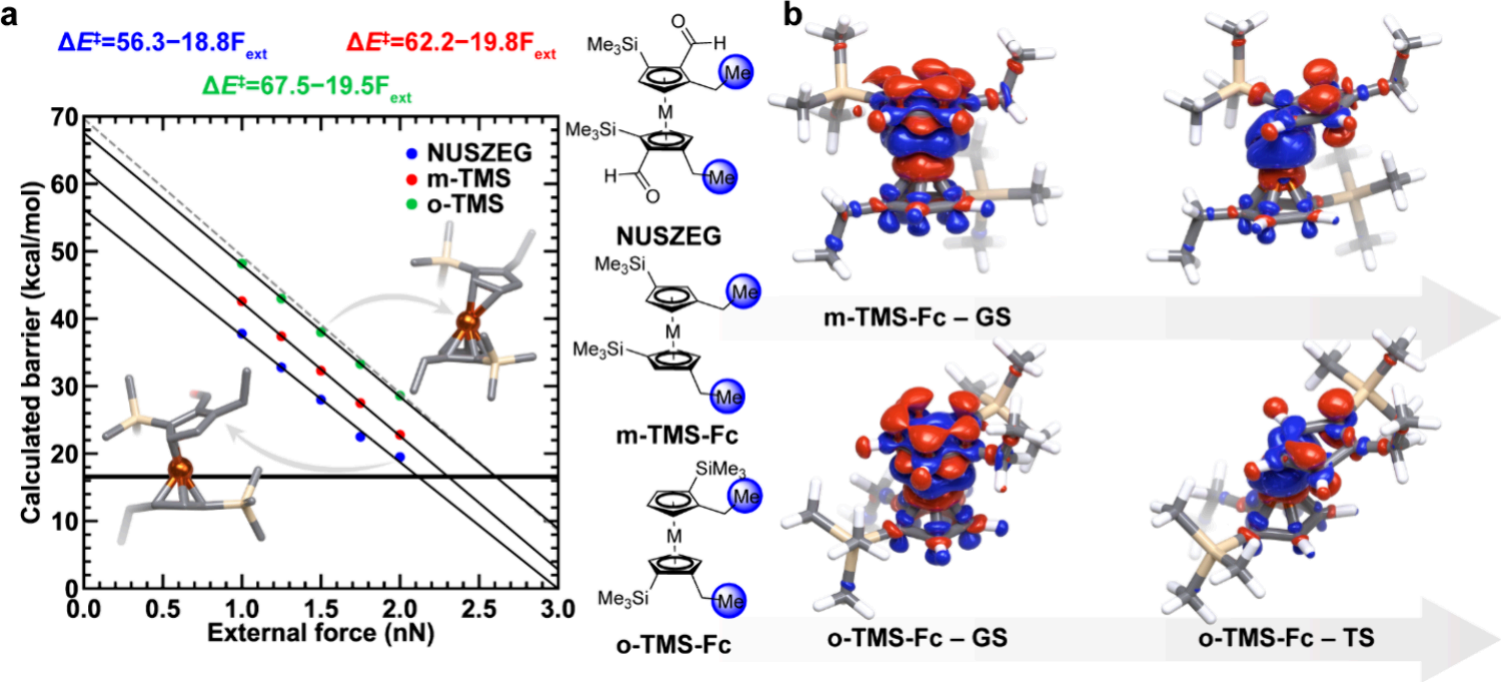
Force-modified ferrocene ligand dissociation of complexes with steric bulk. **a**, Calculated ligand dissociation barriers of **NUSZEG**, m-TMS-Fc, and o-TMS-FC complexes as a function of force. Linear fits for each curve are shown as black lines and their equations are shown at top. For reference, the linear fit of unsubstituted ferrocene is shown as a dashed gray line. The threshold energy of 16.6 kcal/mol that can be used to estimate activation forces measured by single molecule force spectroscopy is shown as a bolded black horizontal line. Schematic representations of relevant complexes are shown in the middle with atoms that had force applied to them highlighted as blue spheres. **b**, Density difference plots of the full complex, with dissociating ligand and the rest of the complex in the ground and transition states at 1 nN external force, where red corresponds to negative density and blue corresponds to positive density.

### 2.4 Experimental Studies of m-TMS-Ferrocene

With a promising simulated mechanochemical reactivity and relatively low synthetic complexity, we chose to pursue m-TMS-Fc as an ideal target to experimentally validate our computational screening of ferrocene mechanophores with enhanced reactivity. This selection highlights a practical advantage of computational discovery: the mechanism of silyl facilitation of mechanochemical cleavage was unanticipated by us, and so we were unlikely to experimentally explore either the TMS substituent or the importance of its position on the ferrocene until after many rounds of significant experimental iteration, which would have been extremely laborious. The mechanochemical lability of m-TMS-Fc was experimentally characterized by exploiting the previously established competition between chain scission and the reaction of nonscissile mechanophores that are randomly incorporated along the same polymer backbone. We have previously used *gem*-dichlorocyclopropane (*g*DCC) mechanophores as the nonscissile mechanophore for this purpose, and we employ that same design here to compare the relative lability of m-TMS-Fc to that of a ferrocene derivative that is unsubstituted except for the polymer attachments (Figure 7).

**Figure 7:**
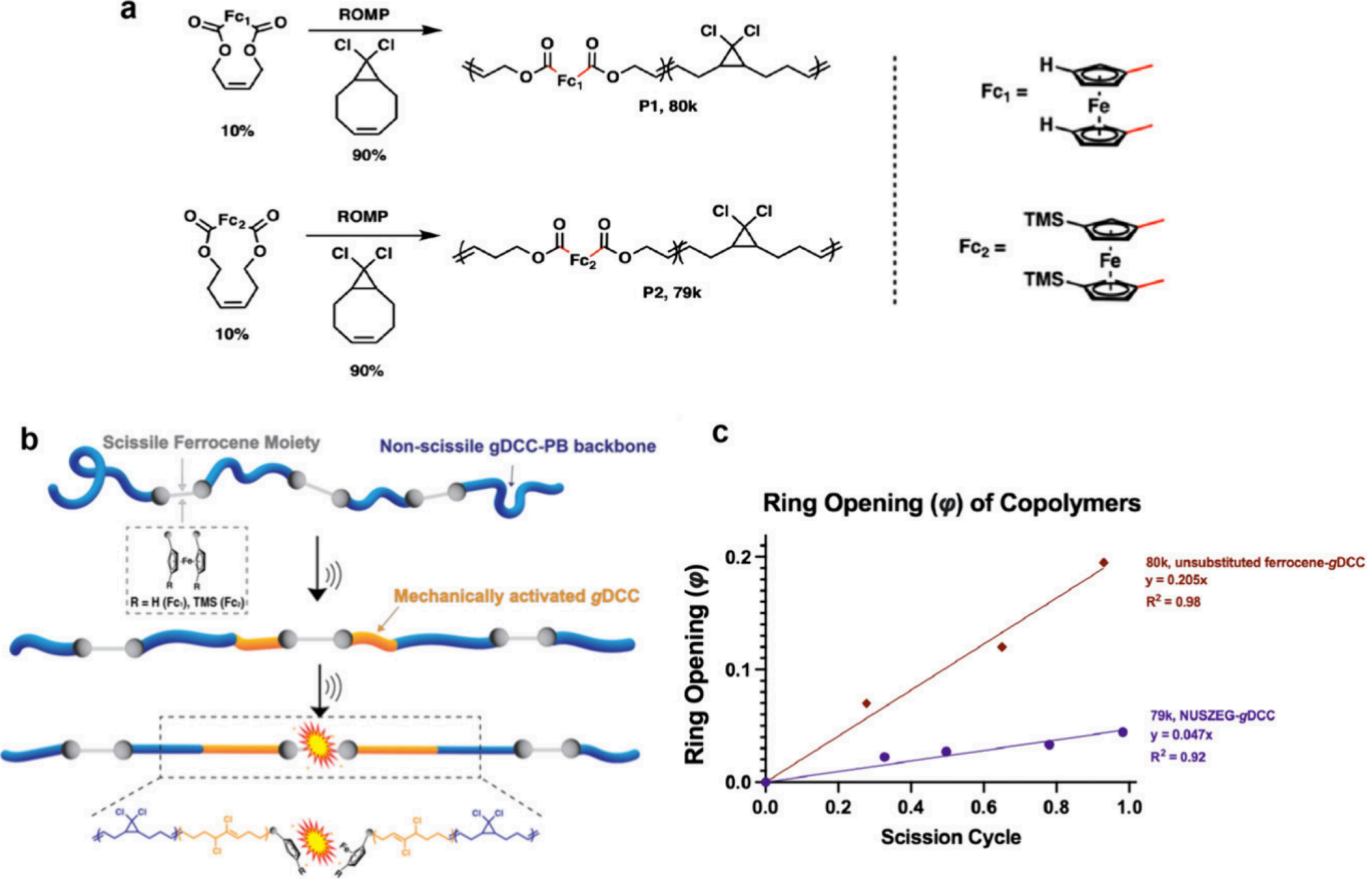
Synthesis of copolymers P1 and P2, competition graphic, and ring opening values. **a**, Synthesis of **P1** ferrocene-*g*DCC copolymer and **P2** m-TMS-Fc-*g*DCC copolymer by entropically driven ring-opening metathesis polymerization (ED-ROMP). **b**, Schematic depiction of the competition between scissile ferrocene bonds and nonscissile *g*DCC. The picture is illustrative only, and it is likely that the specific sites of mechanochemistry are not rigorously central to the overall polymer. **c**, Ring opening values as a function of the scission cycle, where the amount of *g*DCC ring-opening is expressed as φ, the fraction of *g*DCC mechanophores that have reacted.

Copolymers containing unsubstituted ferrocene/*g*DCC (**P1**, number-averaged molecular mass, *M*_n_ = 81 kDa) and m-TMS/*g*DCC (**P2**, *M*_n_ = 79 kDa), each with 10 mol % of its ferrocene repeat, were synthesized by ring-opening metathesis polymerization (ROMP). The polymers were then characterized by pulsed ultrasonication of solutions of the polymers, which is the most commonly employed technique for characterizing mechanochemical reactivity in polymers. Sonication leads to cavitation of gas bubbles within the solution, and the bubble collapse creates an elongational flow field in the solution that rushes to center of the collapsing bubbles and stretches polymer chains caught within it. As the polymers stretch, force increases, and two mechanochemical events are possible. First, the *g*DCC mechanophores can open in a nonscissile fashion (i.e., without the polymer breaking) to give a 2,3-dichloroalkene product that can be quantified by ^1^H NMR. These ring-opening reactions continue to occur along high-force regions of the polymer until either the bubble completes its collapse or the second mechanochemical event occurs—the polymer chain breaks, which can be quantified by size exclusion chromatography and multiangle light scattering. Because neither the *g*DCC nor its ring opened product are themselves sites of preferred scission, the extent of *g*DCC reactivity per chain breaking event gives an indication of the relative mechanical strength of the weakest bonds along the rest of the polymer backbone.^56,57^

By employing this approach, pulsed ultrasonication in tetrahydrofuran confirmed the computational predictions (Figure 7). The amount of *g*DCC ring opening is expressed as φ, the fraction of *g*DCC mechanophores that have reacted, and we evaluate φ relative to the number of scission events per chain in order to achieve a direct competition. Consistent with the predictions, when normalized to the amount of chain scission, the fraction of *g*DCC that reacts is a factor of 4 greater for **P1** than **P2**. In other words, **P1** sustains larger forces before breaking than does **P2**, and because the two polymers differ only in substitution on the ferrocene mechanophore, the computationally discovered m-TMS-Fc is breaking at a lower force than the unsubstituted ferrocene. Additional evidence supporting the enhanced scissile reactivity of m-TMS-Fc is found in the fact that **P2** breaks at a greater initial rate than **P1** under identical sonication conditions, and that **P2** will ultimately break down into smaller fragments than **P1** in response to extended sonication (Supporting Information Figures S29–S32).

Finally, we evaluated how the enhanced reactivity of the discovered m-TMS-Fc mechanophore translates to bulk material properties. The installation of sufficiently reactive mechanophores in the junctions of randomly cross-linked networks has recently been shown to lead to enhancements in tearing energy.^12,58^ The mechanochemical reactivity is critical to this effect because the mechanism requires that, when a polymer strand between two cross-links is under high tension, one of the cross-linkers will break prior to scission of the strand itself. This occurs even if the cross-linker is under less tension than the strand in question. The selective scission of the cross-linker effectively increases the length of the highly tensioned strand, which now extends to the next cross-linker in sequence along the primary chain within the network. The effective lengthening of the highly tensioned strand leads to more elastic energy that must be stored in the strand when it stretched up to the point that it eventually breaks or (if no more cross-linkers remain) slips out of the network to allow a growing crack to propagate.^12^ The magnitude of the effect depends on the mechanochemical lability of the scissile cross-linker.^12^ For example, a more labile diphenylcyclobutane is known to give a much greater enhancement in tearing energy than its less reactive dialkylcyclobutane analog in polyacrylate networks. By tearing energy, we refer to the energy stored in applied stress to the network that is necessary for a crack to propagate through the material. A larger tearing energy, therefore, corresponds to a material that is more difficult to tear. The sonication experiments had confirmed experimentally in single chains what the calculations predicted, namely that m-TMS-Fc will break at lower force than the other, more conventional components of the polymer. Based on our earlier studies,^12^ we therefore expected that same preferential mechanochemical reactivity to give rise to mechanophore toughening effects in material tearing tests, even though the sonication and tearing experiments are very different techniques.

Gratifyingly and strikingly, the computationally guided discovery of m-TMS-Fc yielded a cross-linker that not only validated its enhanced lability in single-chain experiments but also translated into a substantial improvement in macroscopic tear resistance—demonstrating predictive control over bulk material performance. Three networks were made under identical conditions except for the identity of the cross-linker employed. Controlled free radical polymerization of *n*-butyl acrylate with an azobis­(isobutyronitrile) initiator and 4-cyano-4-(((dodecylthio)­carbonothioyl)­thio)­pentanoic acid chain transfer agent (Monomer:CTA:Initiator, 1:1400:2000) in the presence of a diacrylate cross-linker (∼1% with respect to monomer) resulted in a series of elastomers with indistinguishable moduli and swelling ratios (Supporting Information Figures S33–S34 and Text S11). Network **N1** was made with butanediol diacrylate (**C1**), **N2** with ferrocene diacrylate **(C2)**, and **N3** with m-TMS-Fc diacrylate (**C3**). In a pure shear tensile test, the m-TMS-Fc network **N3** is significantly more extensible than either **N2** or **N1** (Figure 8). The resistance to failure is observed as well in notched (Rivlin-Thomas) tearing tests, with the measured tearing energies of 183, 82, and 38 J/m^2^ of **N3**, **N2**, and **N1**, respectively, aligned with the relative mechanochemical activity of the cross-linkers (**C3** > **C2** ≫ **C1**, Figure 8, Video S1). Thus, the mechanistic insights into metallocene mechanochemistry provided by our computational approach are complemented by a practical advance—the successful translation of those insights into the discovery of bulk polymer network compositions with enhanced tear resistance (Supporting Information Figure S35).

**Figure 8:**
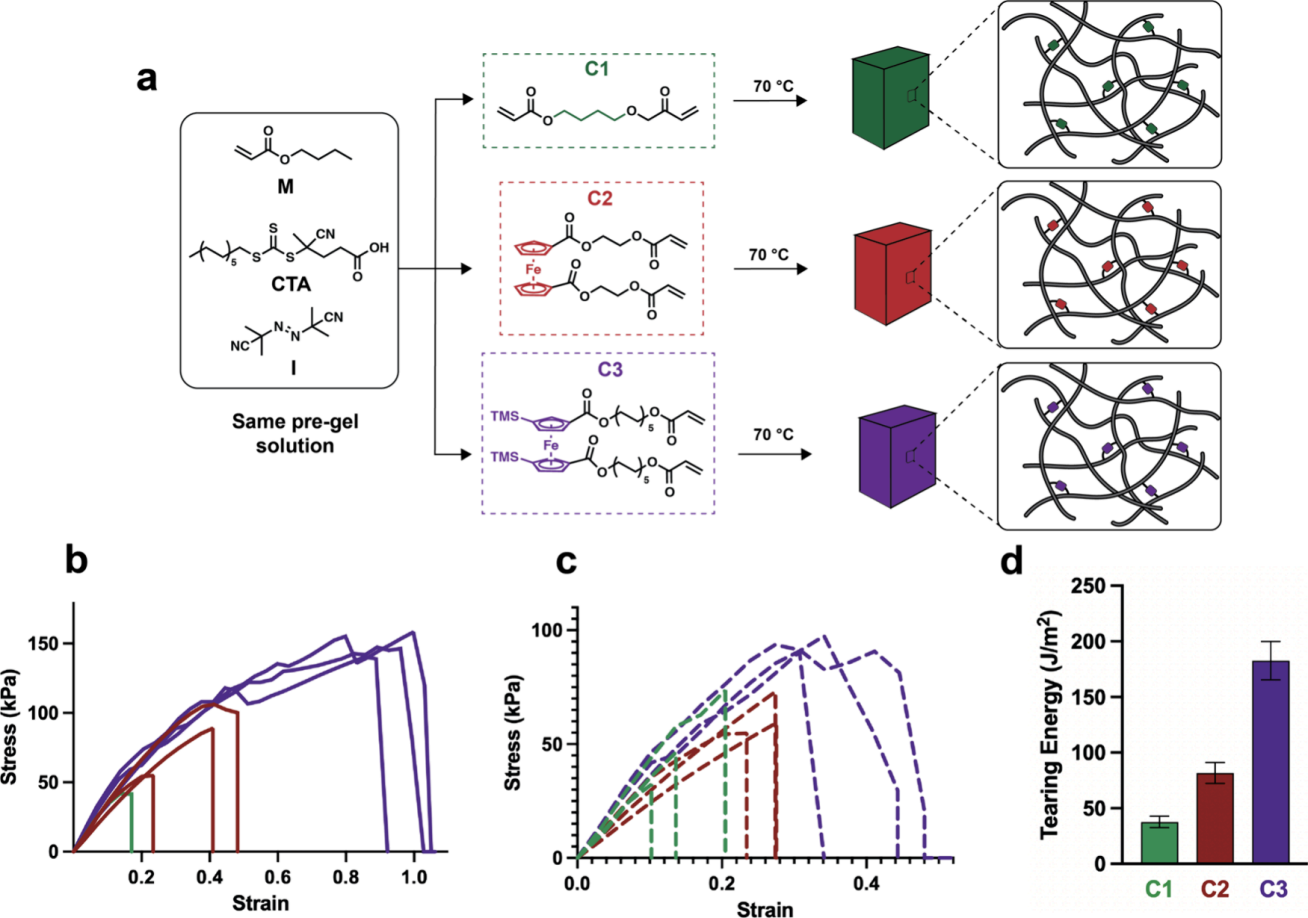
Synthesis and characterization of networks. **a**, Pregel solutions containing equal amounts of monomer, chain transfer agent, and thermal initiator in DMF are degassed and added to stoichiometrically equivalent amounts of cross-linker. The networks are cured via thermal reversible addition-fragmentation chain transfer (RAFT) polymerization. **b**, Pure shear tensile tests. **c**, shear tearing tests, and **d**, tearing energies calculated by the Rivlin-Thomas method.

## 3 Conclusions

In this work, we developed and utilized a high-throughput virtual screening workflow, employing previously synthesized ferrocene complexes to discover novel, synthesizable mechanophores that exhibit enhanced mechanical lability. We demonstrated that combining high-throughput virtual screening with detailed mechanistic insights accelerated the screening and ultimately the identification of novel transition metal mechanophores. Our mechanistic analysis revealed different design strategies to modulate force-promoted reactivity. Enhanced scission is possible through either steric crowding of dissociating ligands, rotational locking of Cp rings with ligand–ligand noncovalent interactions, or through the introduction of functional groups that lead to transition-state-favoring metal–ligand interactions. Such design principles could likely be generalized to other transition metal mechanophores where interligand interactions are capable of modulating reactivity. A computationally promising complex was synthesized, and its enhanced mechanochemical activity in polymer strands was verified experimentally. Finally, we find that the enhanced mechanochemistry predicted computationally and verified in polymer strands translates to bulk materials, as evidenced by enhanced tearing energies in ferrocene-cross-linked *n*-butyl acrylate elastomers. The latter exemplifies the potential of a combination of machine learning and molecule-based mechanistic insights to accelerate material discovery efforts in polymer networks.

This high-throughput platform lays a foundation for the rational integration of mechanically responsive motifs into advanced material design pipelines, offering both a mechanistic framework and a practical toolkit for tailoring polymer durability at the molecular level. Future incorporation of a broader range of the identified ferrocene mechanophores with varying mechanochemical reactivity into networks will lead to a better understanding of the relationship between mechanophore kinetics and bulk material properties. The approach has the potential to further integrate diverse mechanophore discovery into advanced polymer material design, including those based on renewable, recyclable feedstocks. We foresee the capacity to increase the useful lifetime of materials as a contributor to sustainable solutions that address current societal challenges in plastic waste accumulation. In addition, mechanochemical scission of other mechanophore motifs has been used to trigger drug release^10^ and signal damage in polymeric materials,^9^ and the workflow established here should be translatable to those objectives. By illuminating how molecular-scale features control macroscopic material response, this work charts a path toward the predictive design of next-generation soft materials. In cases where extensive previously synthesized variations of a mechanophore are not available, in silico functionalization of a known molecular unit could be pursued (in conjunction with scores for synthetic complexity or accessibility) in order to provide a data set for the development of structure–reactivity relationships and the discovery of improved mechanophores.

## 4 Computational Details

### 4.1 Data Set Curation

The data set of ferrocene complexes was curated using the Cambridge Structural Database (CSD)^59^ version 5.42 (November 2020). The procedure employed the Conquest graphical interface and the CSD Python API. Each component molecule within the crystal structure (refcode) was screened to select molecules that contained a single transition metal with distinct molecular weight and no unknown elements using the CSD Python API. The quality of the crystal structure (i.e., R-factor) was not used as a filtering constraint. Missing hydrogen atoms were added using CSD Python API tools. Duplicate structures with identical connectivity were discarded using the molecular graph determinant.^60^ To sample the regiochemistry of polymer attachment, the NetworkX^61^ shortest path function was utilized to identify target hydrogen atoms across each Cp ring that were subsequently replaced with ethyl groups using a custom script with molSimplify,^62^ available on the Zenodo repository^63^ associated with this paper. Duplicate regioisomers of the ethyl-substituted complexes were discarded using the molecular graph determinant.

### 4.2 Electronic Structure Calculations

We employed a developer version of the GPU-accelerated TeraChem v1.9 ^64,65^ code to carry out the high-throughput screening DFT calculations. All calculations were carried out using the range-separated hybrid ωPBEh^66^ functional with the semiempirical D3 ^67^ dispersion correction and using Becke–Johnson damping.^68–70^ The LACVP* basis set was used, employing 6–31G* for light atoms (H–Ar) and the LANL2DZ^71^ effective core potential for other atoms, including Fe. All calculations were carried out as closed-shell singlets in a restricted formalism, and level shifting^72^ of 0.25 Ha was applied to aid self-consistent field (SCF) convergence. A closed-shell singlet was chosen because past experimental and computational work has demonstrated that mechanochemical ferrocene cleavage proceeds through heterolytic cleavage,^41^ and closed-shell treatment has the added benefit of being lower in computational cost. All calculations were performed with an accelerated implementation of the conductor-like polarizable continuum model (C-PCM) model^73,74^ using a dielectric of ε = 78.39 to model water solvent and aid convergence of heterolytic cleavage. Initial geometries were optimized with the translation rotation internal coordinate (TRIC) optimizer,^75^ using the BFGS algorithm with default convergence thresholds of a maximum energy gradient of 4.5 × 10^–4^ hartree/bohr and an energy difference between steps of 10^–6^ hartree. The constrained geometries simulate external force (CoGEF) calculations were carried out by increasing terminal carbon atom distances at 0.2 Å increments using the TRIC optimizer, while all other internal coordinates were allowed to relax (Figure 2).

Force-modified potential energy surfaces were constructed using the external force is explicitly included (EFEI) method, as implemented in ORCA v. 5.0.1. External forces in 250 pN increments in the range of 1–2 nN were applied to the terminal carbon atoms. All geometry optimizations and calculations in ORCA were performed using the r^2^SCAN-3c composite functional,^76^ which utilizes the r^2^SCAN^77^ meta-GGA functional, a modified version of the def2-TZVP basis set^78^ (mTZVPP), the D4 correction,^79^ and geometrical counterpoise correction.^80^ This low-cost, highly optimized, method was chosen to accelerate the optimization of several transition state structures needed to build force dependence curves. To show that the results obtained using the low-cost r^2^SCAN-3c composite functional are similar to those obtained with a range-separated hybrid used in the CoGEF protocol, we computed force-modified ligand dissociation activation energies of unsubstituted ferrocene using the ωB97X-D4/def2-TZVP method, with geometries optimized at ωB97X-D4/def2-SVP level of theory. We observe no noticeable differences between the two methods in the force-modified activation energies (Supporting Information Figure S37). Calculations were carried out using the C-PCM implicit solvent model with a dielectric of ε = 7.25 to model the experimental THF solvent in sonication experiments. To ensure that no spin crossover occurs at highly tensioned states, we evaluated the vertical spin-splitting energies for the m-TMS-Fc complex at 1 and 2 nN external applied force (Supporting Information Table S6). These results indicate that, regardless of the level of theory, the singlet state is significantly more stable than the quintet state. Geometry optimizations were carried out using the BFGS algorithm in redundant internal coordinates implemented to the default tolerances of 3 × 10^–4^ hartree/bohr for the maximum gradient and 5 × 10^–6^ hartree for the change in energy between steps. Transition states (TSes) were optimized in three stages. First, a relaxed surface scan for the longest Fe–C bond was performed in increments of 0.1 Å at 1.0 nN external force. The converged highest-energy constrained structure was then used to carry out a partitioned rational function optimization (P-RFO) to locate the transition state structure at 1.0 nN. The optimized TS structure was then used as a guess structure for the P-RFO optimization to locate the transition state structure at the nearest external force increment and the final step was repeated until the TS structure was obtained at 2.0 nN. The validity of transition states as first-order saddle points was confirmed using frequency calculations.

### 4.3 Featurization and Machine Learning Models

Complexes were featurized using revised autocorrelations (RACs),^49^ which were computed using molSimplify. RACs are connectivity-based representations that have been successfully applied to transition metal complex property predictions.^49,50,81,82^ RAC features are generated from molecular graphs of a complex or a ligand, where a vertex and an unweighted edge represent each atom and each bond, respectively. Each RAC feature is the sum of products or the sum of differences of heuristic atomic properties (electronegativity, nuclear charge, covalent radius, topology, and identity) at depth *d* (i.e., the number of bonds separating two atoms) on a molecular graph. The RACs in this work include features that span the entire complex, or ligands, where every atom is used as a starting atom in RACs, as well as features that are centered around either the transition metal atom or centered around the attachment point carbon atom (i.e., a carbon atom where an associated hydrogen atom was replaced with an ethyl group), which were introduced specifically to better model mechanochemistry with a maximum depth *d* = 5. The increased depth of autocorrelations used here, compared to commonly employed maximum depth of *d* = 3, was motivated by common structural motifs near a metal atom (i.e., Fe with Cp rings). Overall, the set of RACs used here consists of 150 total features (Supporting Information Text S8).

ANN models were trained using the Keras software package^83^ with Tensorflow^84^ as the backend. Hyperparameters were selected using Hyperopt^85^ (Supporting Information Table S7). We used a 64/16/20 stratified random train/validation/test split. Hyperparameters were selected using the validation set with binary cross-entropy as the figure of merit. For random forest models, features were eliminated using a recursive feature elimination cross-validation (RFECV) algorithm implemented in scikit-learn v1.3.2 ^86^ using 10 stratified folds. Random forest models were trained using the RFECV subset of features utilizing a 5-fold split for cross-validation. Random forest models were trained with hyperparameters selected using Hyperopt (Supporting Information Table S8). We used an 80/20 random train/test split (stratified for classification) and hyperparameters were optimized using repeated k-fold cross-validation, with five folds and three repeats.

### 4.4 Experimental Methods

All experimental methods and characterization details can be found in the Supporting Information (Supporting Information Text S9–S12, Figures S38–S48).

## Supplementary Material







## Data Availability

All data are available in the main text, , and a Zenodo repository.^63^

## Notes

Data set curation and transition metal distribution among metallocenes; classification task setup and model performance (ROC, PR curves, classifier probabilities, feature selection); analysis of second-generation candidates using retrained models; CoGEF simulations examining force–structure relationships (e.g., *F*_max_ vs dihedral angle, NCI energy, charge, orbital energies, dipole moments, steric descriptors); electronic and noncovalent interaction analyses; random forest and ANN model parameters and feature sets; experimental methods for small molecule and polymer synthesis, ferrocene incorporation, sonication studies, SEC overlays, rheological testing, and swelling measurements; mechanophore discovery workflow; validation using alternative DFT functionals; and full characterization of key compounds (NMR, ESI-MS) (PDF)
Video S1: Side-by-side tearing comparison of networks at identical stoichiometries containing, from left to right, a covalent linker (N1), a ferrocene control linker (N2), and m-TMS-ferrocene linker (N3); Video sped up ∼80× (AVI)
Transparent Peer Review report available (PDF)

## References

[1] Li J., Nagamani C., Moore J. S. (2015). Polymer Mechanochemistry: From Destructive
to Productive. Acc. Chem. Res..

[2] Chen Z., Zhu X., Yang J., Mercer J. A. M., Burns N. Z., Martinez T. J., Xia Y. (2020). The Cascade
Unzipping of Ladderane Reveals Dynamic Effects in Mechanochemistry. Nat. Chem..

[3] Küng R., Göstl R., Schmidt B. M. (2022). Release of Molecular
Cargo from Polymer
Systems by Mechanochemistry. Chem. Eur. J..

[4] Wang Z. J., Jiang J., Mu Q., Maeda S., Nakajima T., Gong J. P. (2022). Azo-Crosslinked
Double-Network Hydrogels Enabling Highly
Efficient Mechanoradical Generation. J. Am.
Chem. Soc..

[5] Wang Z. J., Li W., Li X., Nakajima T., Rubinstein M., Gong J. P. (2025). Rapid Self-Strengthening
in Double-Network Hydrogels
Triggered by Bond Scission. Nat. Mater..

[6] Jiang, J. ; Kubota, K. ; Jin, M. ; Wang, Z. J. ; Nakajima, T. ; Ito, H. ; Gong, J. P. ; Maeda, S. Computational Exploration of Polymer Mechanochemistry: Quantitation of Activation Force and Systematic Discovery of Reaction Sites Utilizing Two Forces. ChemRxiv, Nov. 23, 2022. 10.26434/chemrxiv-2022-fr09l. PMC12426933 40882976

[7] Groote R., Jakobs R. T. M., Sijbesma R. P. (2013). Mechanocatalysis:
Forcing Latent
Catalysts into Action. Polym. Chem..

[8] Ong M. T., Leiding J., Tao H., Virshup A. M., Martínez T. J. (2009). First Principles
Dynamics and Minimum Energy Pathways for Mechanochemical Ring Opening
of Cyclobutene. J. Am. Chem. Soc..

[9] Stratigaki M., Göstl R. (2020). Methods for
Exerting and Sensing Force in Polymer Materials
Using Mechanophores. ChemPlusChem..

[10] Shi Z., Song Q., Göstl R., Herrmann A. (2021). Mechanochemical Activation
of Disulfide-Based Multifunctional Polymers for Theranostic Drug Release. Chemical Science.

[11] Wang S., Beech H. K., Bowser B. H., Kouznetsova T. B., Olsen B. D., Rubinstein M., Craig S. L. (2021). Mechanism Dictates
Mechanics: A Molecular Substituent Effect in the Macroscopic Fracture
of a Covalent Polymer Network. J. Am. Chem.
Soc..

[12] Wang S., Hu Y., Kouznetsova T. B., Sapir L., Chen D., Herzog-Arbeitman A., Johnson J. A., Rubinstein M., Craig S. L. (2023). Facile Mechanochemical Cycloreversion of Polymer Cross-Linkers
Enhances Tear Resistance. Science.

[13] Zhao X. (2014). Multi-Scale
Multi-Mechanism Design of Tough Hydrogels: Building Dissipation into
Stretchy Networks. Soft Matter.

[14] Lei H., Dong L., Li Y., Zhang J., Chen H., Wu J., Zhang Y., Fan Q., Xue B., Qin M., Chen B., Cao Y., Wang W. (2020). Stretchable Hydrogels
with Low Hysteresis and Anti-Fatigue Fracture Based on Polyprotein
Cross-Linkers. Nat. Commun..

[15] Wang Z., Xiang C., Yao X., Le Floch P., Mendez J., Suo Z. (2019). Stretchable Materials of High Toughness
and Low Hysteresis. Proc. Natl. Acad. Sci. U.
S. A..

[16] Ducrot E., Chen Y., Bulters M., Sijbesma R. P., Creton C. (2014). Toughening
Elastomers with Sacrificial Bonds and Watching Them Break. Science.

[17] Lin S., Liu X., Liu J., Yuk H., Loh H.-C., Parada G. A., Settens C., Song J., Masic A., McKinley G. H., Zhao X. (2019). Anti-Fatigue-Fracture Hydrogels. Sci. Adv..

[18] Wang Z., Zheng X., Ouchi T., Kouznetsova T. B., Beech H. K., Av-Ron S., Matsuda T., Bowser B. H., Wang S., Johnson J. A., Kalow J. A., Olsen B. D., Gong J. P., Rubinstein M., Craig S. L. (2021). Toughening Hydrogels
through Force-Triggered Chemical Reactions That Lengthen Polymer Strands. Science.

[19] Li B., Cao P.-F., Saito T., Sokolov A. P. (2023). Intrinsically Self-Healing
Polymers: From Mechanistic Insight to Current Challenges. Chem. Rev..

[20] Law K. L., Starr N., Siegler T. R., Jambeck J. R., Mallos N. J., Leonard G. H. (2020). The United States’ Contribution
of Plastic Waste
to Land and Ocean. Sci. Adv..

[21] Geyer R., Jambeck J. R., Law K. L. (2017). Production,
Use, and Fate of All
Plastics Ever Made. Sci. Adv..

[22] Bao Y., Luo Z., Cui S. (2020). Environment-Dependent
Single-Chain Mechanics of Synthetic
Polymers and Biomacromolecules by Atomic Force Microscopy-Based Single-Molecule
Force Spectroscopy and the Implications for Advanced Polymer Materials. Chem. Soc. Rev..

[23] McFadden M. E., Overholts A. C., Osler S. K., Robb M. J. (2023). Validation of an
Accurate and Expedient Initial Rates Method for Characterizing Mechanophore
Reactivity. ACS Macro Lett..

[24] Ribas-Arino J., Marx D. (2012). Covalent Mechanochemistry:
Theoretical Concepts and Computational
Tools with Applications to Molecular Nanomechanics. Chem. Rev..

[25] Roessler A. G., Zimmerman P. M. (2018). Examining the Ways to Bend and Break Reaction Pathways
Using Mechanochemistry. J. Phys. Chem. C.

[26] Huo Z., Skala S. J., Falck L. R., Laaser J. E., Statt A. (2022). Computational
Study of Mechanochemical Activation in Nanostructured Triblock Copolymers. ACS Polymers Au.

[27] Li Y., Xue B., Yang J., Jiang J., Liu J., Zhou Y., Zhang J., Wu M., Yuan Y., Zhu Z., Wang Z. J., Chen Y., Harabuchi Y., Nakajima T., Wang W., Maeda S., Gong J. P., Cao Y. (2024). Azobenzene as a Photoswitchable Mechanophore. Nat. Chem..

[28] Koti
Ainavarapu S. R., Wiita A. P., Dougan L., Uggerud E., Fernandez J. M. (2008). Single-Molecule Force Spectroscopy Measurements of
Bond Elongation during a Bimolecular Reaction. J. Am. Chem. Soc..

[29] Smalo̷ H. S., Uggerud E. (2012). Ring Opening Vs. Direct Bond Scission of the Chain
in Polymeric Triazoles under the Influence of an External Force. Chem. Commun..

[30] Horst M., Meisner J., Yang J., Kouznetsova T. B., Craig S. L., Martínez T. J., Xia Y. (2024). Mechanochemistry of
Pterodactylane. J. Am. Chem. Soc..

[31] Cardosa-Gutierrez M., De Bo G., Duwez A.-S., Remacle F. (2023). Bond Breaking of Furan-Maleimide
Adducts Via a Diradical Sequential Mechanism under an External Mechanical
Force. Chemical Science.

[32] Liu Y., Holm S., Meisner J., Jia Y., Wu Q., Woods T. J., Martinez T. J., Moore J. S. (2021). Flyby Reaction
Trajectories:
Chemical Dynamics under Extrinsic Force. Science.

[33] Sun Y., Kevlishvili I., Kouznetsova T. B., Burke Z. P., Craig S. L., Kulik H. J., Moore J. S. (2024). The Tension-Activated Carbon–Carbon
Bond. Chem.

[34] Bell G. I. (1978). Models
for the Specific Adhesion of Cells to Cells. Science.

[35] Stauch T., Dreuw A. (2016). Advances in Quantum Mechanochemistry: Electronic Structure Methods
and Force Analysis. Chem. Rev..

[36] Beyer M. K. (2000). The Mechanical
Strength of a Covalent Bond Calculated by Density Functional Theory. J. Chem. Phys..

[37] Klein I. M., Husic C. C., Kovács D. P., Choquette N. J., Robb M. J. (2020). Validation of the Cogef Method as a Predictive Tool
for Polymer Mechanochemistry. J. Am. Chem. Soc..

[38] Nixon R., De Bo G. (2020). Three Concomitant C-C
Dissociation Pathways during the Mechanical
Activation of an N-Heterocyclic Carbene Precursor. Nat. Chem..

[39] Di
Giannantonio M., Ayer M. A., Verde-Sesto E., Lattuada M., Weder C., Fromm K. M. (2018). Triggered Metal
Ion Release and Oxidation: Ferrocene as a Mechanophore in Polymers. Angew. Chem., Int. Ed..

[40] Sha Y., Zhang Y., Xu E., McAlister C. W., Zhu T., Craig S. L., Tang C. (2019). Generalizing Metallocene Mechanochemistry
to Ruthenocene Mechanophores. Chem. Sci..

[41] Sha Y., Zhang Y., Xu E., Wang Z., Zhu T., Craig S. L., Tang C. (2018). Quantitative
and Mechanistic Mechanochemistry
in Ferrocene Dissociation. ACS Macro Lett..

[42] Zhang Y., Wang Z., Kouznetsova T. B., Sha Y., Xu E., Shannahan L., Fermen-Coker M., Lin Y., Tang C., Craig S. L. (2021). Distal Conformational Locks on Ferrocene
Mechanophores
Guide Reaction Pathways for Increased Mechanochemical Reactivity. Nat. Chem..

[43] Larik F. A., Saeed A., Fattah T. A., Muqadar U., Channar P. A. (2017). Recent
Advances in the Synthesis, Biological Activities and Various Applications
of Ferrocene Derivatives. Appl. Organomet. Chem..

[44] Rupf S. M., Sievers R., Riemann P. S., Reimann M., Kaupp M., Fasting C., Malischewski M. (2023). Persilylation
of Ferrocene: The Ultimate
Discipline in Sterically Overcrowded Metal Complexes. Dalton Transactions.

[45] Wang W.-Y., Ma N.-N., Sun S.-L., Qiu Y.-Q. (2014). Redox Control of
Ferrocene-Based Complexes with Systematically Extended Π-Conjugated
Connectors: Switchable and Tailorable Second Order Nonlinear Optics. Phys. Chem. Chem. Phys..

[46] Siemeling U., Auch T.-C. (2005). 1,1′-Di­(Heteroatom)-Functionalised Ferrocenes
as [N, N], [O, O] and [S, S] Chelate Ligands in Transition Metal Chemistry. Chem. Soc. Rev..

[47] Moriuchi T., Hirao T. (2010). Design of Ferrocene-Dipeptide Bioorganometallic Conjugates to Induce
Chirality-Organized Structures. Acc. Chem. Res..

[48] Cha Y., Zhu T., Sha Y., Lin H., Hwang J., Seraydarian M., Craig S. L., Tang C. (2021). Mechanochemistry
of Cationic Cobaltocenium
Mechanophore. J. Am. Chem. Soc..

[49] Janet J. P., Kulik H. J. (2017). Resolving Transition
Metal Chemical Space: Feature
Selection for Machine Learning and Structure-Property Relationships. J. Phys. Chem. A.

[50] Janet J. P., Chan L., Kulik H. J. (2018). Accelerating Chemical
Discovery with
Machine Learning: Simulated Evolution of Spin Crossover Complexes
with an Artificial Neural Network. J. Phys.
Chem. Lett..

[51] Nandy A., Duan C., Goffinet C., Kulik H. J. (2022). New Strategies for
Direct Methane-to-Methanol Conversion from Active Learning Exploration
of 16 Million Catalysts. JACS Au.

[52] Rosenblum M., Woodward R. (1958). The Structure and Chemistry
of Ferrocene. Iii. Evidence
Pertaining to the Ring Rotational Barrier. J.
Am. Chem. Soc..

[53] Shrake A., Rupley J. A. (1973). Environment and Exposure to Solvent of Protein Atoms.
Lysozyme and Insulin. J. Mol. Biol..

[54] Verloop, A. In Pesticide Chemistry: Human Welfare and Environment; Doyle, P. , Fujita, T. , Eds.; Pergamon, 1983; pp 339–344. 10.1016/B978-0-08-029222-9.50051-2.

[55] Horn P. R., Mao Y., Head-Gordon M. (2016). Probing Non-Covalent Interactions with a Second Generation
Energy Decomposition Analysis Using Absolutely Localized Molecular
Orbitals. Phys. Chem. Chem. Phys..

[56] Lenhardt J. M., Ogle J. W., Ong M. T., Choe R., Martinez T. J., Craig S. L. (2011). Reactive Cross-Talk
between Adjacent Tension-Trapped
Transition States. J. Am. Chem. Soc..

[57] Lee B., Niu Z., Wang J., Slebodnick C., Craig S. L. (2015). Relative Mechanical
Strengths of Weak Bonds in Sonochemical Polymer Mechanochemistry. J. Am. Chem. Soc..

[58] Watabe T., Aoki D., Otsuka H. (2022). Polymer-Network Toughening and Highly
Sensitive Mechanochromism Via a Dynamic Covalent Mechanophore and
a Multinetwork Strategy. Macromolecules.

[59] Groom C. R., Bruno I. J., Lightfoot M. P., Ward S. C. (2016). The Cambridge Structural
Database. Acta Crystallogr. B.

[60] Taylor M. G., Yang T., Lin S., Nandy A., Janet J. P., Duan C., Kulik H. J. (2020). Seeing
Is Believing: Experimental
Spin States from Machine Learning Model Structure Predictions. J. Phys. Chem. A.

[61] Hagberg A., Swart P., S Chult D. (2008). Exploring Network Structure,
Dynamics,
and Function Using Networkx. Proc. 7th Python
Sci. Conf..

[62] Ioannidis E. I., Gani T. Z. H., Kulik H. J. (2016). Molsimplify:
A Toolkit for Automating
Discovery in Inorganic Chemistry. J. Comput.
Chem..

[63] Zenodo Repository for High-Throughput Discovery of Ferrocene Mechanophores with Enhanced Reactivity and Network Toughening. 10.5281/zenodo.12770056. (Accessed July 2024). PMC12550622 41142348

[64] Petachem. http://www.petachem.com. (Accessed April 1, 2020).

[65] Ufimtsev I. S., Martinez T. J. (2009). Quantum Chemistry
on Graphical Processing Units. 3.
Analytical Energy Gradients, Geometry Optimization, and First Principles
Molecular Dynamics. J. Chem. Theory Comput..

[66] Rohrdanz M. A., Martins K. M., Herbert J. M. (2009). A Long-Range-Corrected
Density Functional
That Performs Well for Both Ground-State Properties and Time-Dependent
Density Functional Theory Excitation Energies, Including Charge-Transfer
Excited States. J. Chem. Phys..

[67] Grimme S., Antony J., Ehrlich S., Krieg H. (2010). A Consistent and Accurate
Ab Initio Parametrization of Density Functional Dispersion Correction
(Dft-D) for the 94 Elements H-Pu. J. Chem. Phys..

[68] Becke A. D., Johnson E. R. (2005). A Density-Functional
Model of the Dispersion Interaction. J. Chem.
Phys..

[69] Johnson E. R., Becke A. D. (2005). A Post-Hartree–Fock
Model of Intermolecular
Interactions. J. Chem. Phys..

[70] Johnson E. R., Becke A. D. (2006). A Post-Hartree-Fock
Model of Intermolecular Interactions:
Inclusion of Higher-Order Corrections. J. Chem.
Phys..

[71] Hay P. J., Wadt W. R. (1985). Ab Initio Effective
Core Potentials for Molecular Calculations.
Potentials for the Transition Metal Atoms Sc to Hg. J. Chem. Phys..

[72] Saunders V. R., Hillier I. H. (1973). A “Level–Shifting” Method for
Converging Closed Shell Hartree-Fock Wave Functions. Int. J. Quantum Chem..

[73] Liu F., Luehr N., Kulik H. J., Martínez T. J. (2015). Quantum
Chemistry for Solvated Molecules on Graphical Processing Units Using
Polarizable Continuum Models. J. Chem. Theory
Comput..

[74] Lange A. W., Herbert J. M. (2010). A Smooth, Nonsingular, and Faithful Discretization
Scheme for Polarizable Continuum Models: The Switching/Gaussian Approach. J. Chem. Phys..

[75] Wang L.-P., Song C. (2016). Geometry Optimization
Made Simple with Translation and Rotation Coordinates. J. Chem. Phys..

[76] Grimme S., Hansen A., Ehlert S., Mewes J.-M. (2021). R2scan-3c: A “Swiss
Army Knife” Composite Electronic-Structure Method. J. Chem. Phys..

[77] Furness J. W., Kaplan A. D., Ning J., Perdew J. P., Sun J. (2020). Accurate and
Numerically Efficient R2scan Meta-Generalized Gradient Approximation. J. Phys. Chem. Lett..

[78] Weigend F., Ahlrichs R. (2005). Balanced Basis Sets of Split Valence,
Triple Zeta Valence
and Quadruple Zeta Valence Quality for H to Rn: Design and Assessment
of Accuracy. Phys. Chem. Chem. Phys..

[79] Caldeweyher E., Ehlert S., Hansen A., Neugebauer H., Spicher S., Bannwarth C., Grimme S. (2019). A Generally Applicable
Atomic-Charge Dependent London Dispersion Correction. J. Chem. Phys..

[80] Kruse H., Grimme S. (2012). A Geometrical Correction
for the Inter- and Intra-Molecular
Basis Set Superposition Error in Hartree-Fock and Density Functional
Theory Calculations for Large Systems. J. Chem.
Phys..

[81] Nandy A., Duan C., Janet J. P., Gugler S., Kulik H. J. (2018). Strategies
and Software for Machine Learning Accelerated Discovery in Transition
Metal Chemistry. Ind. Eng. Chem. Res..

[82] Janet J. P., Gani T. Z. H., Steeves A. H., Ioannidis E. I., Kulik H. J. (2017). Leveraging Cheminformatics Strategies
for Inorganic
Discovery: Application to Redox Potential Design. Ind. Eng. Chem. Res..

[83] Chollet, F. Keras, 2015. https://keras.io/. (Retrieved 12/20/2023).

[84] Abadi, M. ; Agarwal, A. ; Barham, P. ; Brevdo, E. ; Chen, Z. ; Citro, C. ; Corrado, G. S. ; Davis, A. ; Dean, J. ; Devin, M. ; Ghemawat, S. ; Goodfellow, I. ; Harp, A. ; Irving, G. ; Isard, M. ; Jozefowicz, R. ; Jia, Y. ; Kaiser, L. ; Kudlur, M. ; Levenberg, J. ; Mané, D. ; Schuster, M. ; Monga, R. ; Moore, S. ; Murray, D. ; Olah, C. ; Shlens, J. ; Steiner, B. ; Sutskever, I. ; Talwar, K. ; Tucker, P. ; Vanhoucke, V. ; Vasudevan, V. ; Viégas, F. ; Vinyals, O. ; Warden, P. ; Wattenberg, M. ; Wicke, M. ; Yu, Y. ; Zheng, X. Tensorflow, 2015. https://www.tensorflow.org/. (Retrieved 12/20/2023).

[85] Bergstra J.
C. D. D., Yamins D. (2013). Hyperopt:
A Python Library for Optimizing the Hyperparameters
of Machine Learning Algorithms. Proc. 12th Python
Sci. Conf..

[86] Pedregosa F., Varoquaux G., Gramfort A., Michel V., Thirion B., Grisel O., Blondel M., Prettenhofer P., Weiss R., Dubourg V. (2011). Scikit-Learn:
Machine Learning in
Python. J. Mach. Learn. Res..

