# Supplementary material for: High-Throughput Discovery of Ferrocene Mechanophores with Enhanced Reactivity and Network Toughening
Source: ACS Cent Sci. 2025 Aug 1;11(10):1839–51. doi: 10.1021/acscentsci.5c00707 (PMC12550622; doi:10.1021/acscentsci.5c00707)
Supplement: Supplementary file 1 [file oc5c00707_si_001.pdf]

**Supporting Information for**
***High-Throughput Discovery of Ferrocene Mechanophores with Enhanced Reactivity and Network Toughening***

Ilia Kevlishvili[1,2], Jafer Vakil[2,3], David W. Kastner,[1,4] Xiao Huang[1,2,5], Stephen L. Craig[2,3], and Heather J. Kulik[1,2,5,*]

[1]Department of Chemical Engineering, Massachusetts Institute of Technology, Cambridge, MA 02139, USA

[2]NSF Center for the Chemistry of Molecularly Optimized Networks, Duke University, Durham, NC 27708, USA

[3]Department of Chemistry, Duke University, Durham, NC 27708, USA

[4]Department of Biological Engineering, Massachusetts Institute of Technology, Cambridge, MA 02139, USA

[5]Department of Chemistry, Massachusetts Institute of Technology, Cambridge, MA 02139, USA

[*]email: hjkulik@mit.edu

**Contents**

**Text S1** Dataset curation Page S3
**Figure S1** Count of transition metal identity among metallocene complexes Page S4
**Text S2** Description of RACs feature set Page S5
**Text S3** Boundary for the classification task Page S6
**Figure S2** Precision recall and receiver operating characteristic curves Page S7
**Table S1** Performance of the classifier with different decision boundaries Page S7
**Figure S3** The classifier predicted probability of ferrocene complexes Page S8
**Text S4** Identification of the second generation of complexes Page S9
**Figure S4** ROC curve and PR curve of the retrained test set Page S9
**Figure S**5 The ROC curve and the PR curve of the retrained classifier Page S10
**Figure S6** Retrained classifier probability of unbridged ferrocenes by # atoms Page S11
**Figure S7** CoGEF simulated $F_{max}$ values vs the handle dihedral angle Page S11
**Figure S8** CoGEF simulations of unbridged ferrocenes Page S12
**Text S5** Analysis of ligand ligand interaction energies Page S12
**Figure S9** CoGEF simulation relationship between $F_{max}$ and dihedral angle Page S13
**Figure S10** NCI visualization of a representative structure, NAGBEE Page S13
**Figure S11** NCI visualization of a representative structure, ORIMEJ Page S14
**Figure S12** CoGEF simulated $F_{max}$ values vs of the NCI energy colored by density Page S15
**Figure S13** CoGEF simulated $F_{max}$ values vs the NCI energy colored by dihedral Page S15
**Figure S14** CoGEF simulated $F_{max}$ values vs the charge on the iron Page S16
**Figure S15** CoGEF simulated $F_{max}$ values vs the HOMO energies Page S16
**Figure S16** CoGEF simulated $F_{max}$ values vs the LUMO energies Page S17
**Figure S17** CoGEF simulated $F_{max}$ values vs the complex dipole moment Page S17
**Text S6** Analysis of electronic descriptors Page S18
**Figure S18** CoGEF simulated $F_{max}$ values vs the sterimol B5 minimum Page S18

**Figure S19** CoGEF simulated $F_{max}$ values vs the sterimol B5 maximum Page S19
**Figure S20** CoGEF simulated $F_{max}$ values vs the sterimol L minimum Page S19
**Figure S21** CoGEF simulated $F_{max}$ values vs the sterimol L maximum Page S20
**Figure S22** CoGEF simulated $F_{max}$ values vs the sterimol B1 minimum Page S20
**Figure S23** CoGEF simulated $F_{max}$ values vs the sterimol B1 maximum Page S21
**Table S2** Pearson's correlation of different features with $F_{max}$ Page S22
**Text S7** Feature selection for random forest models Page S23
**Table S3** Features selected for random forest classification task Page S23
**Table S4** Features selected for random forest regression task Page S24
**Figure S24** Distributions of training and test set model errors for maximum force Page S25
**Figure S25** Force-modified $E_a$ for unsubstituted and ansa-cis/trans ferrocenes Page S26
**Figure S26** NCI analysis of EFILAI in the ground and transition states Page S27
**Figure S27** HB analysis of EGIPER under force in the ground and TS Page S27
**Figure S28** Force-modified activation energies for the dissociation of BADXOU Page S28
**Table S5** Energy decomposition analysis for the interaction energies Page S28
**Figure S29** SEC overlay of sonicated polymers containing unsubstituted ferrocene Page S29
**Figure S30** SEC overlay of sonicated polymers containing m-TMS-Fc Page S30
**Figure S31** First order exponential decay in $M_{n,t}$ as a function of sonication time Page S31
**Figure S32** Time evolution of **P2** sonication, and appearance of dichloroalkene peaks Page S32
**Figure S33** Rheological frequency sweep, 0.1-100 rad/s Page S33
**Figure S34** Swelling ratio (Q) by mass Page S34
**Text S8** Synthesis of networks containing Fc crosslinkers Page S34
**Figure S35** A workflow used for the discovery of new ferrocene mechanophores Page S35
**Figure S36** Force-modified activation energies with alternate DFT functionals Page S35
**Table S6** Calculated vertical spin-splitting energies for m-TMS-Fc under tension Page S36
**Table S7** Set of hyperparameters for ANN models Page S36
**Table S8** Set of hyperparameters for random forest models Page S36
**Text S9** General experimental details Page S37
**Text S10** Small molecule synthesis Page S38
**Text S11** Synthesis of ferrocene-containing polymers Page S41
**Figure S37** 1,1′-Bis(trimethylsilyl)ferrocene dicarboxylic acid **(1)** HNMR Page S42
**Figure S38** 1,1′-Bis(trimethylsilyl)ferrocene dicarboxylic acid **(1)** CNMR Page S43
**Figure S39** 1,1′-Bis(trimethylsilyl)ferrocene macrocycle **(3)** HNMR Page S44
**Figure S40** 1,1′-Bis(trimethylsilyl)ferrocene macrocycle **(3)** CNMR Page S45
**Figure S41** Ferrocene diacrylate **(4)** HNMR Page S46
**Figure S42** Ferrocene diacrylate **(4)** CNMR Page S47
**Figure S43** m-TMS-Fc diacrylate **(5)** HNMR Page S48
**Figure S44** m-TMS-Fc diacrylate **(5)** CNMR Page S49
**Figure S45** ESI-MS of **(3)** Confirmed by $H^+$, $Na^+$ adducts Page S50
**Figure S46** ESI-MS of **(4)** Confirmed by $H^+$, $Na^+$ adducts Page S51
**Figure S47** ESI-MS of **(5)** Confirmed by $H^+$, $Na^+$, $K^+$ adducts Page S51
**Figure S48** Structures considered in the first quarry Page S52
**Figure S49** Structures considered in the second quarry Page S52
**Text S12** Description of supplementary video Page S52
**References** Page S52

## Text S1. Dataset Curation

To curate a data set for the discovery of synthesizable metallocene mechanophores, we carried out two CSD queries to identify target molecules. Both searches were limited to complexes with resolved 3D coordinates, and we removed polymeric species. The first search was limited to linear metallocenes where the Cp centroid–metal–Cp centroid angle was required to be greater than 150 degrees. Furthermore, each Cp ring was restricted to containing at most two substituents on the five-membered ring to ensure greater synthetic accessibility (Supporting Information Figure S48). This resulted in the identification of 15,341 complexes, including duplicates. The second search was performed only to locate hetero-metallocenes, where one or both Cp rings had one carbon atom replaced by either nitrogen or phosphorus. Geometric constraints for this search were the same as the first search. Due to the limited size of the second set, no constraint was placed on the number of substitutions applied to the Cp rings (Supporting Information Figure S49). This second search led to the identification of an additional 129 complexes, including duplicates.

The total set of 15,470 complexes from both searches was subsequently filtered by removing any multimetallic species and any duplicate structures (i.e., with the same molecular connectivity), resulting in 5,403 unique complexes. To identify and eliminate these duplicates, we used molecular graph determinants, specifically the determinants of atomic number weighted connectivity matrices. Most of these complexes (i.e., 5,114) contained iron cores (Supporting Information Figure S1). Therefore, we decided to limit our investigation to ferrocene and ferrocenium complexes. Of the iron-containing set, only 557 of these complexes had CSD user-defined charges. To avoid a significant reduction of the data set, we assumed that, unless otherwise specified, a given structure would be a ferrocene core (i.e., Fe (II)) with a total complex charge of 0. This assumption was manually validated for 50 randomly sampled complexes with unassigned charges and was consistent for each of these complexes (see Zenodo repository[1]). To avoid open-shell calculations, we limited our search to complexes with an even number of electrons, which led to the elimination of complexes with explicitly specified Fe(III) oxidation state (for example, refcode: **ALEPUE**) and complexes with radical centers (for example, refcode: **BAFQUV**). This led to the elimination of an additional 76 complexes, with the final set consisting of 5,038 unique ferrocene complexes (Figure 1).

Because the direction of applied forces can significantly impact mechanochemical activation[2-4], we considered different possible regioisomers of polymer incorporation. One hydrogen atom on each Cp ring was replaced with an ethyl group to consider different pulling directions of the applied force. Duplicate regioisomers were identified and removed using molecular graph determinants as described above. After accounting for potential regioisomers of functionalized ferrocene complexes, we obtained a final set of 12,491 structures for mechanochemical activation.

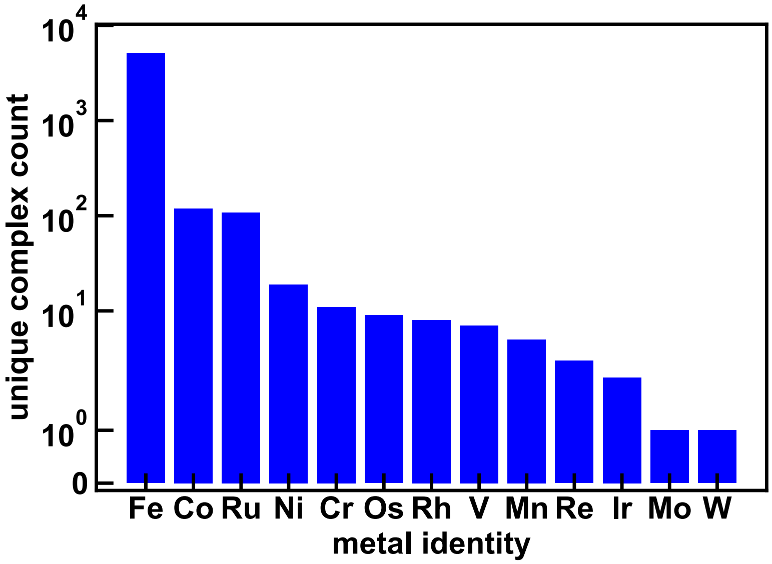


**Figure S1.** Count of transition metal identity among unique metallocene complexes curated from the CSD sorted by frequency. The y-axis (count) is shown on a log scale.

**Text S2.** Description of RACs feature set.

RACs are graph-based representations that are products and differences in the molecular graph of heuristic properties. They therefore incorporate information about connectivity but do not directly encode through-space interactions. A short description of RACs is provided in the main text. Here, we provide more technical details regarding the specific implementation of RACs used in this work to enable reproducibility. For RACs feature set, five atomwise heuristic properties were considered, including topology (*T*), identity (*I*), electronegativity (χ), covalent radius (*S*), and nuclear charge (*Z*). Full complex and ligand full-scope RACs features were considered, where every atom in the complex or ligand can be used as the starting atom. For atom-centered RAC features, either the metal center, or attachment point carbon atoms on the ligand serve as starting atoms. Autocorrelations are defined as

$$P_d = \sum_i \sum_j P_i P_j \delta(d_{ij}, d) \tag{1}$$

$$P'_d = \sum_i \sum_j (P_i - P_j) \delta(d_{ij}, d) \tag{2}$$

where $P_d$ is the graph autocorrelation for property $P$ at depth $d$, and $P'_d$ is the difference graph autocorrelation, $P_i$ is the property $P$ at atom $i$, $\delta$ is the Dirac delta function, and $d_{ij}$ is the through bond distance between atoms $i$ and $j$. For these features, the depth $d_{ij}$ was allowed to vary from zero to five for product RACs. The difference graph autocorrelations were computed for ligand-centered RACs, and depth was allowed to vary from zero to five. The final RAC feature vector consists of 30 full-scope complex RACs, 30 full-scope ligand RACs, 60 metal-centered RACs features, including 30 product RACs and 30 difference RACs, and 60 AP-centered RACs features, including 30 product RACs and 30 difference RACs. This gives a total of 180 RAC features. However, 30 features, including all difference identity RACs (12 total), as well as all zero-depth metal- and AP-centered (20 total, including two difference identity) RACs are constant over the full set. Therefore, this gives a total of 150 RAC features.

**Text S3.** Boundary for the classification task

We selected our ML approach based on the relative merits and flaws of our computational CoGEF protocol. Although CoGEF fails to predict experimentally determined forces based on SMFS experiments quantitatively[5], it provides good qualitative predictions in relative reactivity, consistent with experiments[5]. Therefore, we selected a classification approach as the most suitable strategy for the ML-accelerated discovery of more reactive ferrocene mechanophores to distinguish CoGEF-predicted high and low $F_{max}$ values in order to discover complexes with low $F_{max}$. We chose 3.5 nN as a heuristic cutoff for the two classes, where a complex with $F_{max}$ greater than or equal to 3.5 nN is labeled as member of the negative class with lower mechanochemical reactivity, and a complex with $F_{max}$ below 3.5 nN is identified as a member of the positive class with a higher mechanochemical reactivity. This cutoff was chosen to maximize the number of complexes in the positive class while maintaining a significant separation from the major cluster at $F_{max}$ = 3.8 nN. This approach leads to two imbalanced classes, with only 22% of the dataset assigned a positive label, but small changes (~0.1 nN) to the classifier boundary do not impact the performance of the classifier (Supporting Information Table S1).

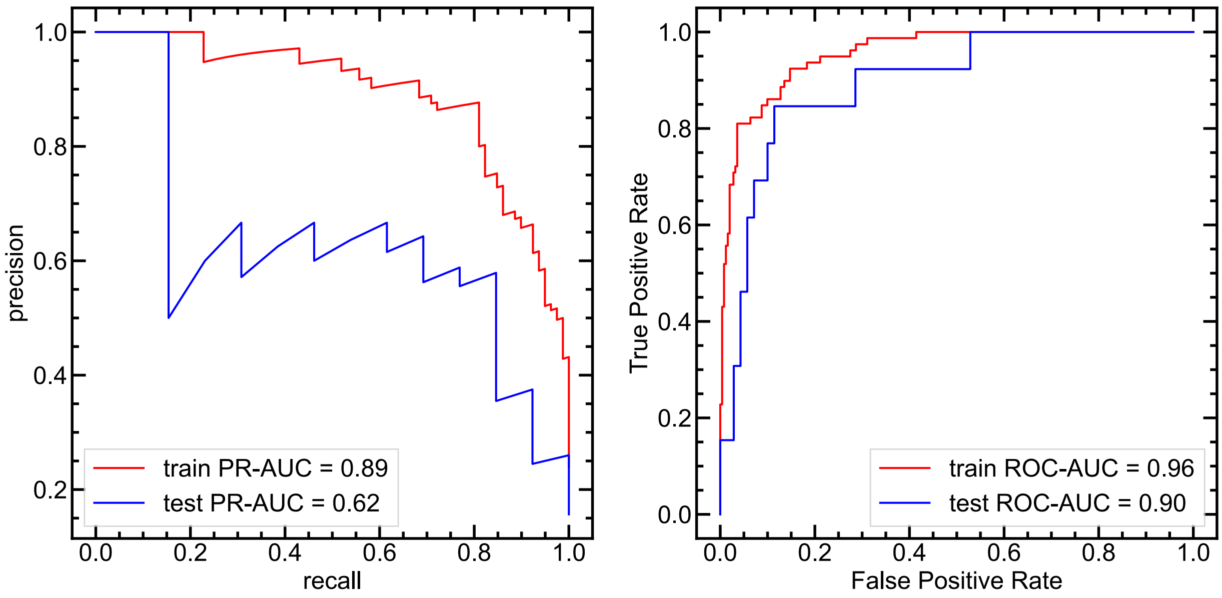


**Figure S2. Precision recall and receiver operating characteristic curves.** Precision recall (left) and receiver operating characteristic (right) curves of an ANN classifier based on modified RAC features predicting the probability of low $F_{max}$ with the data obtained from the initial random screen. The training set curve is shown in red and the test set curve is shown in blue. Areas under the curve are shown in the legend.

**Table S1.** Performance of the classifier trained on data obtained from the initial random screening using a modified RACs feature set and tested on the set-aside test set with different boundaries or different models for the classification task.

| Model | Boundary | ROC-AUC | PR-AUC | Accuracy | Precision | Recall |
|---|---|---|---|---|---|---|
| ANN | 3.5 nN | 0.90 | 0.62 | 0.76 | 0.38 | 0.85 |
| ANN | 3.4 nN | 0.90 | 0.67 | 0.72 | 0.32 | 0.83 |
| ANN | 3.6 nN | 0.90 | 0.62 | 0.76 | 0.41 | 0.80 |
| RF | 3.5 nN | 0.86 | 0.60 | 0.83 | 0.46 | 0.54 |

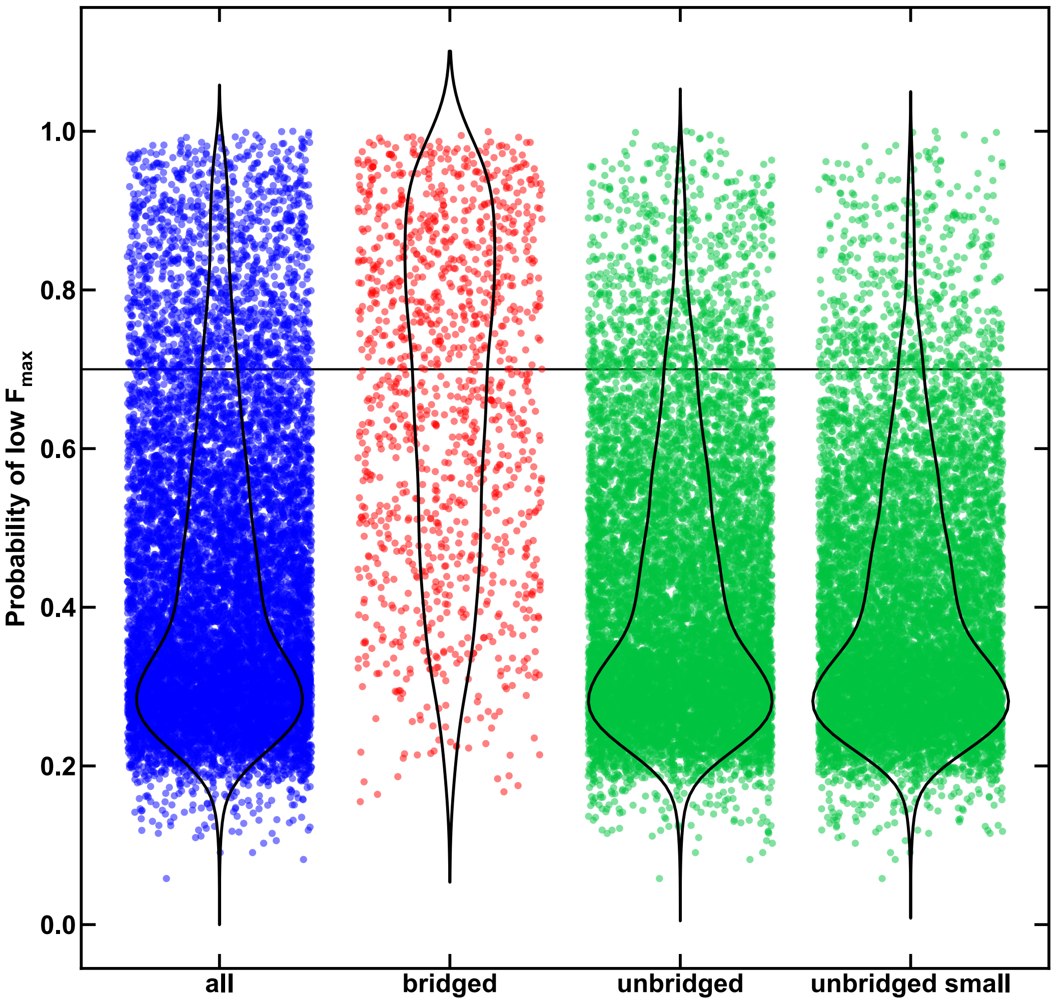


**Figure S3.** The classifier predicted probability of low $F_{max}$ of all ferrocene complexes (blue, 12,078 complexes), bridged ferrocenes (red, 1,168 complexes), unbridged ferrocenes (green, 10,910 complexes) and unbridged ferrocenes restricted by number of atoms (green, 10,231 complexes) not simulated in the randomly selected set. The density distribution of each set is shown with black outlines.

**Text S4**. Identification of the second generation of complexes

Consistent with the random screening approach, a majority of the structures are identified as less reactive by the classifier (Supporting Information Figure S3). Furthermore, decomposing the dataset into unbridged (10,910 complexes) and bridged (1,168 complexes) ferrocenes shows a consistent trend, indicating that the majority of the complexes that are predicted to be more reactive are bridged ferrocenes (Supporting Information Figure S3). Therefore, to uncover additional unbridged ferrocenes with higher reactivity, we only selected unbridged ferrocene complexes that were predicted to be more reactive with a higher degree of certainty (>0.7, Supporting Information Figure S3). To accelerate simulations of these complexes, we removed any complexes with more than 90 atoms, based on the atom counts in the Cambridge Structural Database (CSD) (Supporting Information Figure S3). These constraints led to the selection of 556 complexes for further examination.

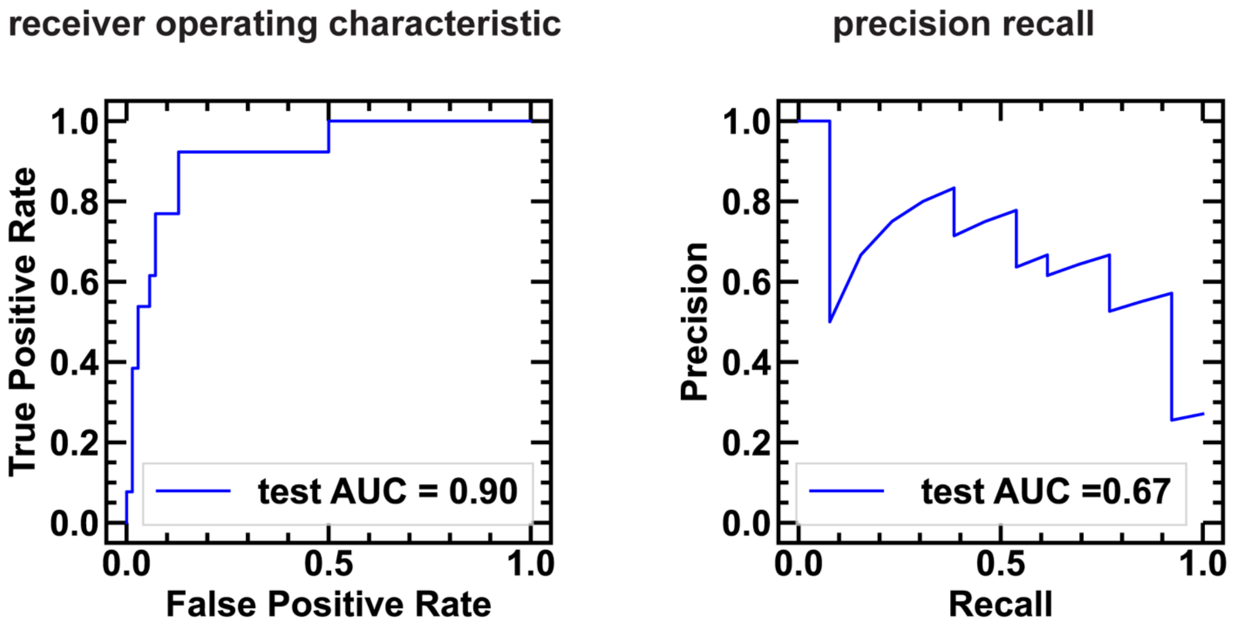


**Figure S4.** Receiver operating characteristic curve (left) and precision recall curve (right) of the retrained classifier on the originally set-aside test set. The area under the curve is shown in the legend.

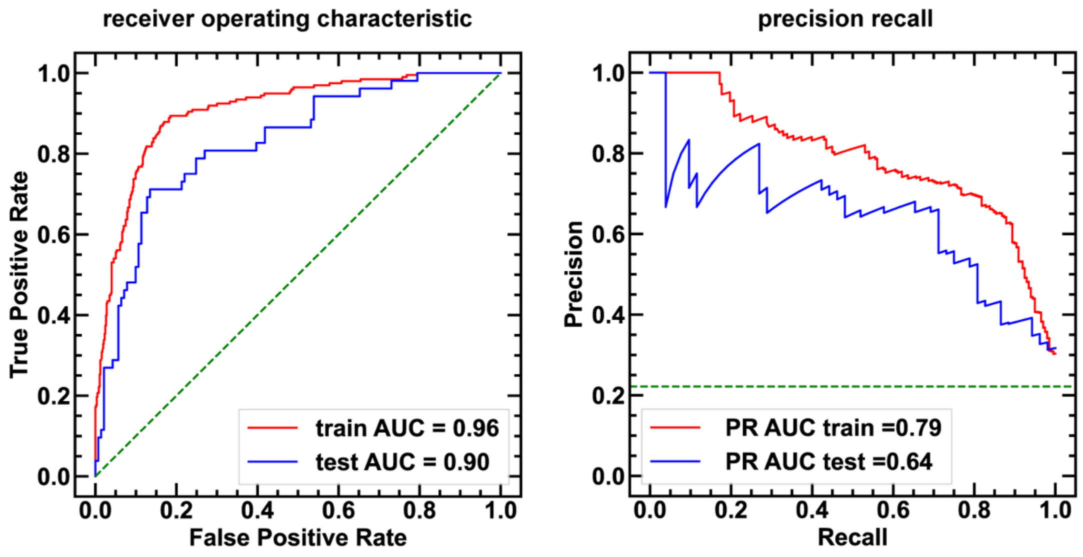


**Figure S5.** The receiver operating characteristic curve (left) and the precision recall curve (right) of the retrained classifier on the new training (red, 771 complexes) and test (blue, 193 complexes) sets that contains both the randomly selected and ML-selected sets. The area under the curve is shown in the legend.

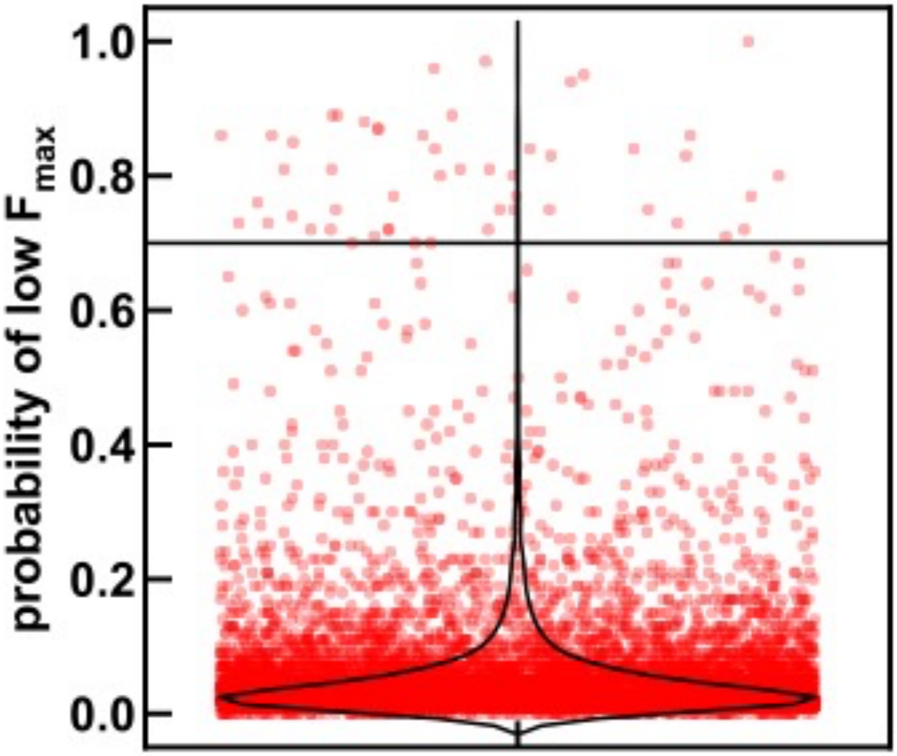


**Figure S6.** The retrained classifier predicted probability of unbridged ferrocenes restricted by number of atoms (red, 9,675 complexes) not simulated in either random or ML-selected sets. The density distribution of these points is shown with black outlines.

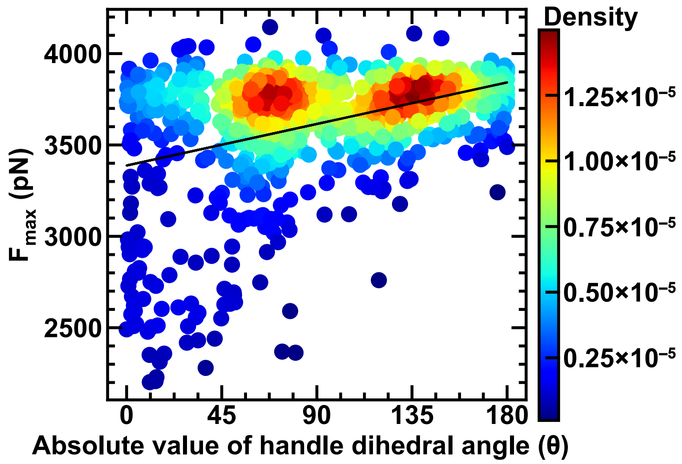


**Figure S7.** CoGEF simulated $F_{max}$ values as a function of the absolute value of the handle dihedral angle in unbridged ferrocenes. Data points are colored by kernel density estimation (KDE) density values, as indicated by the color bar. A linear fit is shown as a black line.

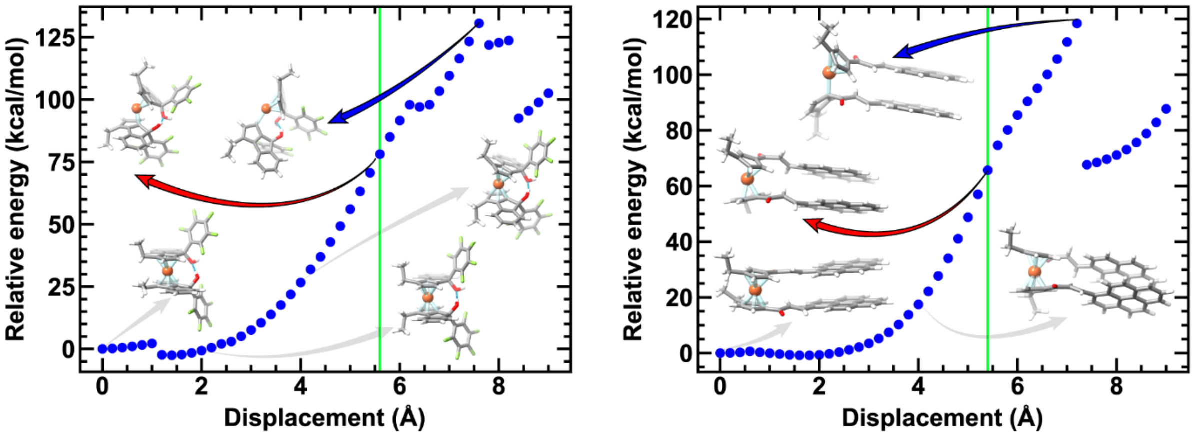


**Figure S8. CoGEF simulations of unbridged ferrocenes.** CoGEF simulation plots of ORIMEJ (left) and NAGBEE (right) complexes. Both complexes are unbridged ferrocenes with no covalent crosslinks between two Cp ligands. Each complex features a favorable non-covalent interaction between two Cp ligands, with hydrogen bonding present in ORIMEJ and extended $\pi$-stacking interaction present in NAGBEE. Both structures undergo a peeling ligand dissociation mechanism that doesn't involve Cp ring rotation. The displacement distance at $F_{max}$ is shown by a light green line. Several structures are shown as insets, including initial structures, structures associated with $F_{max}$ (red arrow), and structures associated with $E_{max}$ (blue arrow). Atoms in the structures are colored as follows: iron in orange, oxygen in red, carbon in gray, hydrogen in white, fluorine in cyan.

**Text S5.** Analysis of ligand–ligand interaction energies.

We calculated ligand–ligand interaction energies as the difference between the energy of the optimized complex with the Fe atom removed and the energies of individual ligands. As expected, ligand–ligand interaction energies alone are only weakly correlated with the force-induced reactivity, with a Pearson correlation coefficient of 0.28 (Supporting Information Figure S12). However, a further inspection of the relationship between $F_{max}$ and the absolute value of dihedral, as well as ligand–ligand interaction energies, reveals that once through-space interactions between ligands become sufficiently favorable (i.e., negative), the absolute value of the dihedral angle becomes correlated to activation energies (Supporting Information Figure S10–S11). In fact, if we consider ligand–ligand interactions that are overall attractive, we find that the absolute value of the dihedral angle has a Pearson correlation coefficient of 0.84 (Supporting Information Figure S9). To further support this hypothesis, we analyzed non-covalent interactions for select complexes using NCIPLOT[6] and quantum theory of atoms in molecule (QTAIM)[7] approaches, confirming strongly favorable through-space interactions for these complexes (Supporting Information Figures S12–S13).

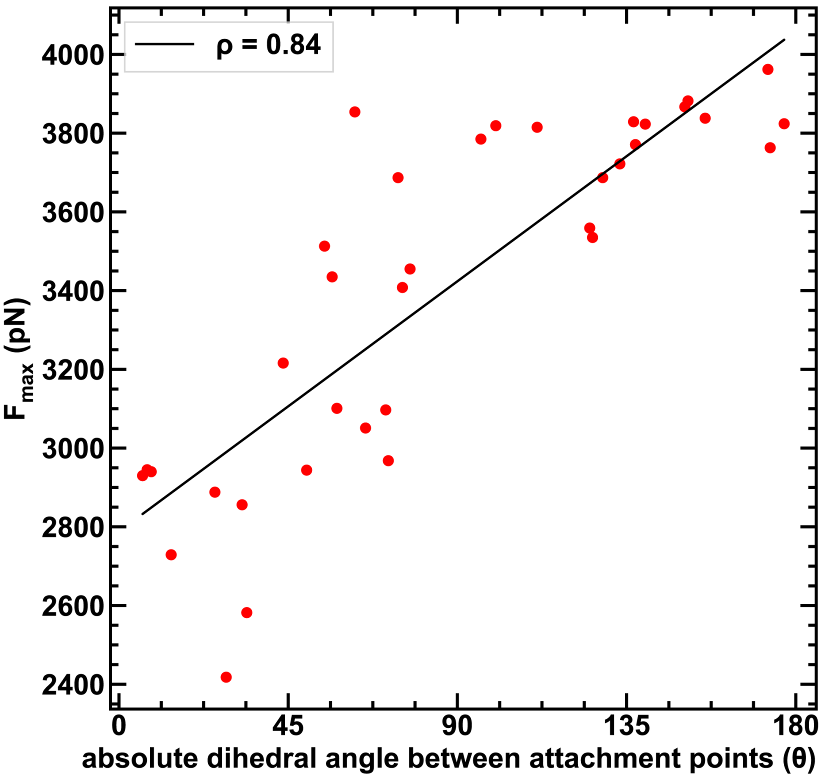


**Figure S9. CoGEF simulation relationship between $F_{max}$ and dihedral angle.** CoGEF simulated $F_{max}$ values as a function of the absolute value of the dihedral angle between attachment points for the subset of unbridged ferrocene complexes with favorable ($\Delta E < 0$) ligand–ligand non-covalent interaction energies. A linear fit is shown as a black line.

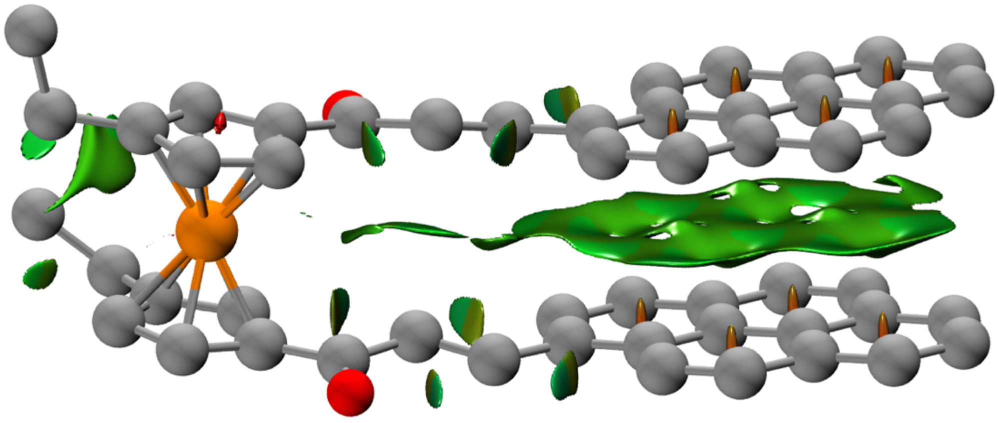

**Figure S10. Non-covalent interaction visualization of a representative structure, NAGBEE.** NCIPLOT analysis of the ground state NAGBEE structure. Hydrogens are omitted. An isovalue of 0.4 was used to construct the NCI surface. Red corresponds to unfavorable interactions, green corresponds to weakly favorable interactions, and blue corresponds to strongly favorable interactions.

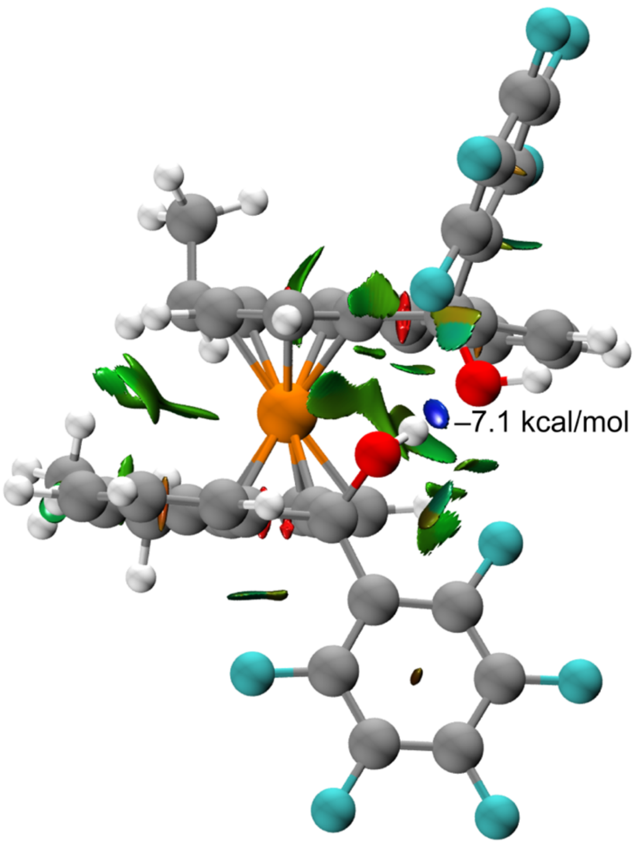


**Figure S11. Non-covalent interaction visualization of a representative structure, ORIMEJ.** NCIPLOT analysis of the ground state ORIMEJ structure. An isovalue of 0.4 was used to construct the NCI surface. Red corresponds to unfavorable interactions, green corresponds to weakly favorable interactions, and blue corresponds to strongly favorable interactions. Hydrogen bonding interaction strength was analyzed using the atoms-in-molecules critical point density using Multiwfn and is shown in the label.

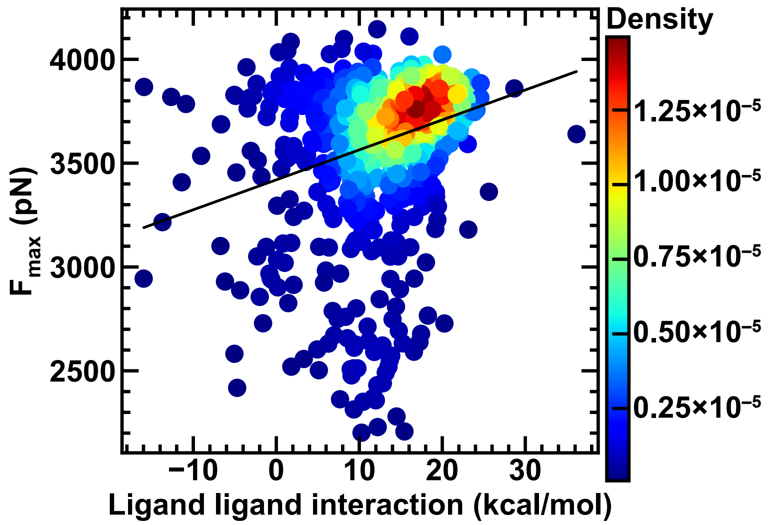


**Figure S12.** CoGEF simulated $F_{max}$ values as a function of the non-covalent interaction energy between two ligands in unbridged ferrocenes. Data points are colored by kernel density estimation (KDE) density values, as indicated by the color bar. A linear fit is shown as a black line.

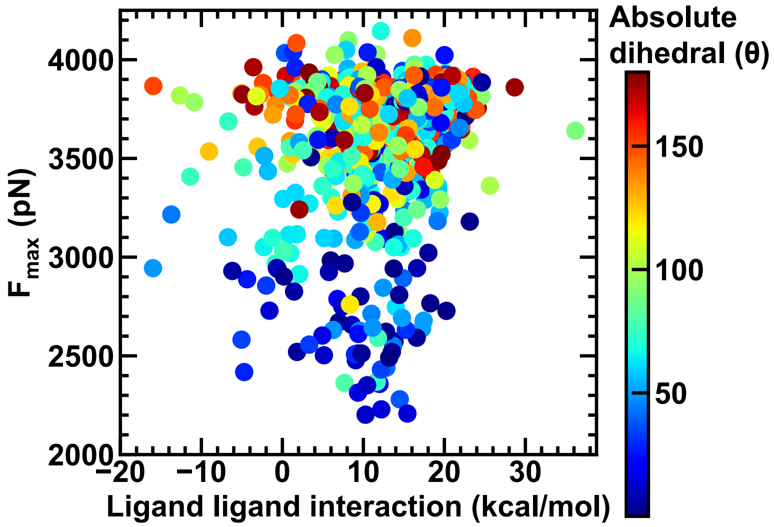


**Figure S13.** CoGEF simulated $F_{max}$ values as a function of the non-covalent interaction energy between two ligands in unbridged ferrocenes. Data points are colored by the absolute dihedral angle (θ), as indicated by the color bar.

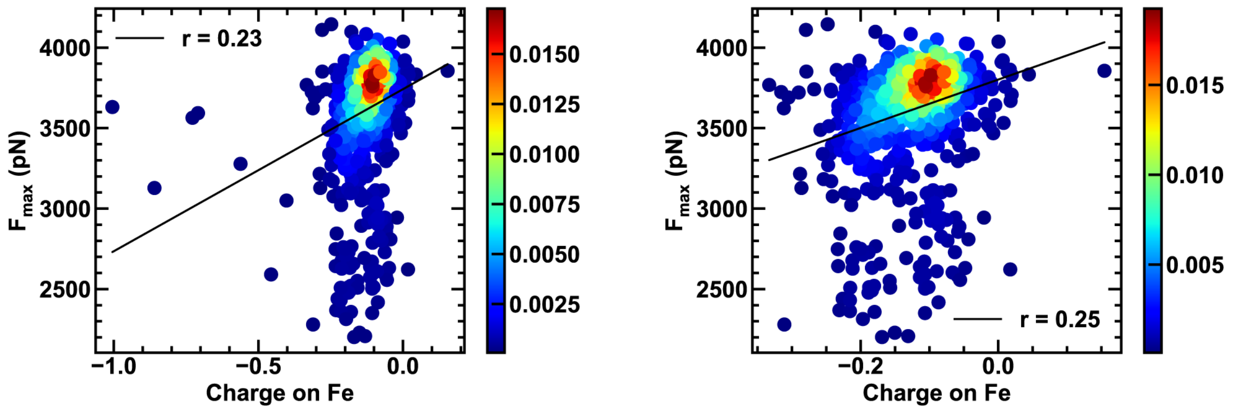


**Figure S14.** CoGEF simulated $F_{max}$ values as a function of the charge on the iron of all unbridged ferrocenes (left) and a subset after dropping highly negatively charged outliers (right). Data points are colored by kernel density estimation (KDE) density values, as indicated by the color bar. A linear fit is shown as a black line.

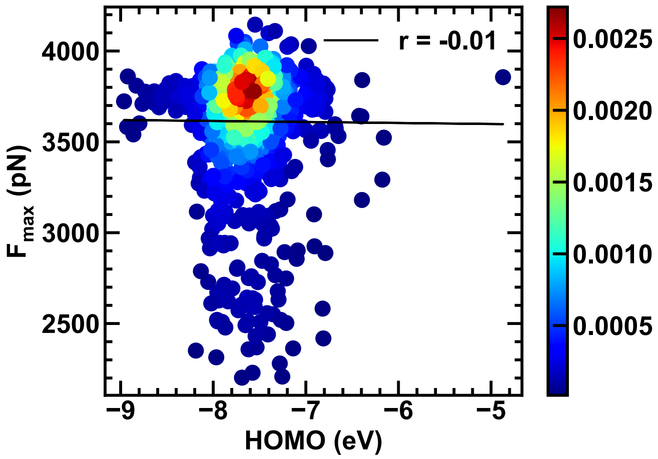


**Figure S15.** CoGEF simulated $F_{max}$ values as a function of the HOMO energies. Data points are colored by kernel density estimation (KDE) density values, as indicated by the color bar. A linear fit is shown as a black line.

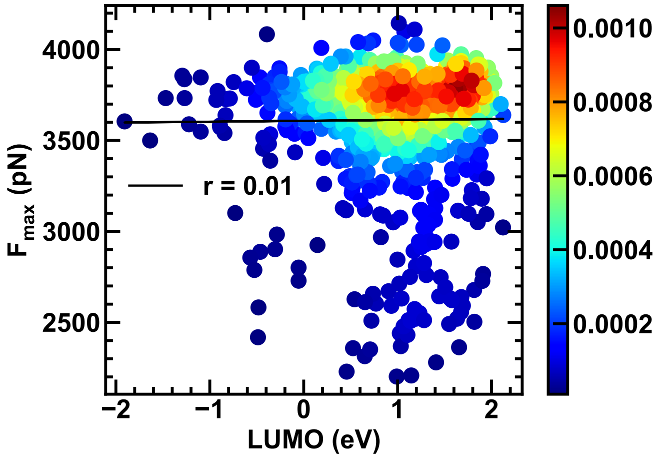


**Figure S16.** CoGEF simulated $F_{max}$ values as a function of the LUMO energies. Data points are colored by kernel density estimation (KDE) density values, as indicated by the color bar. A linear fit is shown as a black line.

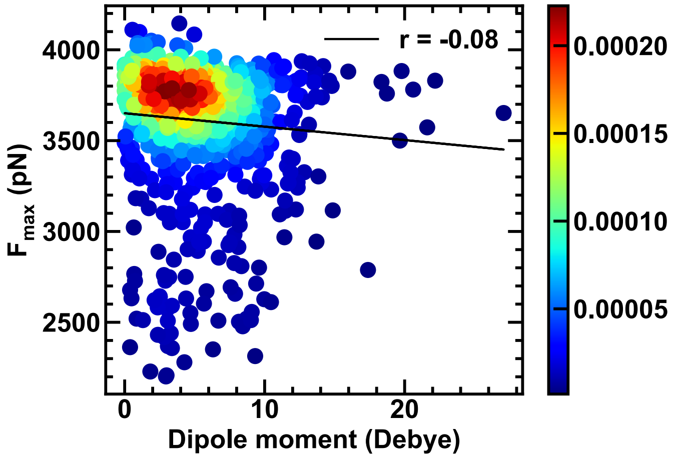


**Figure S17.** CoGEF simulated $F_{max}$ value as a function of the complex dipole moment. Data points are colored by kernel density estimation (KDE) density values, as indicated by the color bar. A linear fit is shown as a black line.

**Text S6.** Analysis of electronic and geometric descriptors.

To analyze how the electronic character of the ground state complex affects mechanochemical reactivity, we considered the HOMO and LUMO energetics, the Mulliken charge on the iron, and the dipole moment of the complex. These electronic characteristics do not correlate strongly with the calculated $F_{max}$ values. In particular, HOMO and LUMO energetics show no relationship with the calculated $F_{max}$, with Pearson correlation coefficients of −0.01 and 0.01, respectively (Supporting Information Figures S15–S16. Similarly, the dipole moment shows a weak correlation with the calculated $F_{max}$ values (Supporting Information Figure S17). On the other hand, we find that the calculated Mulliken charge on the iron has a moderate correlation (0.23) with the computed $F_{max}$ values (Supporting Information Figure S14). This observation can be attributed to the nature of ligand dissociation, which involves heterolytic cleavage. Dissociation of the ligand leads to the accumulation of a positive charge on the transition metal, which can be stabilized by increased electron density on the iron center. These insights highlight that electronic character plays a role in the force-induced activation of ferrocene complexes. However, the degree of tuning available through electronic changes is relatively limited.

In addition to localized SASA, we calculated two sets of Sterimol[8, 9] parameters that describe the length and width of the complex along each of the polymer attachments of the complex, and found that the B1 parameter, which describes the smallest width orthogonal to the polymer attachment, has a moderate negative correlation (−0.31) with $F_{max}$, while other Sterimol parameters (L, B5) are only weakly correlated (Supporting Information Figures S18–S23, Table S2). Both SASA and B1 describe a more metal-local steric environment, whereas L and B5 describe more metal-distant steric environment, suggesting that local steric crowding improves mechanochemical reactivity.

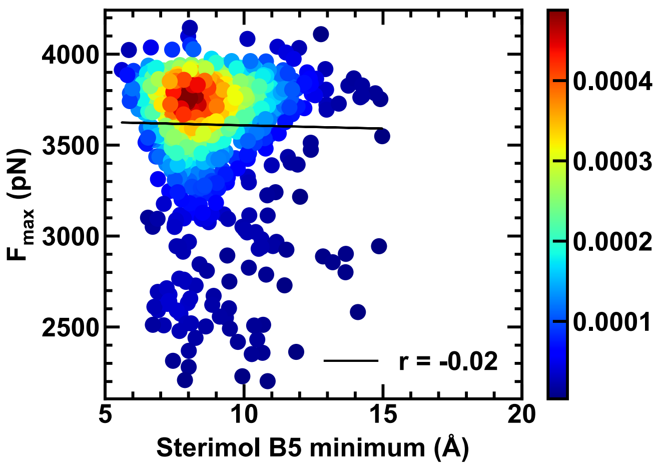


**Figure S18.** CoGEF simulated $F_{max}$ values as a function of the minimum of the B5 parameters calculated for each ligand. The B5 parameter describes the maximum width orthogonal to the ligand attachment to the ethyl group bond. Data points are colored by kernel density estimation (KDE) density values, as indicated by the color bar. A linear fit is shown as a black line.

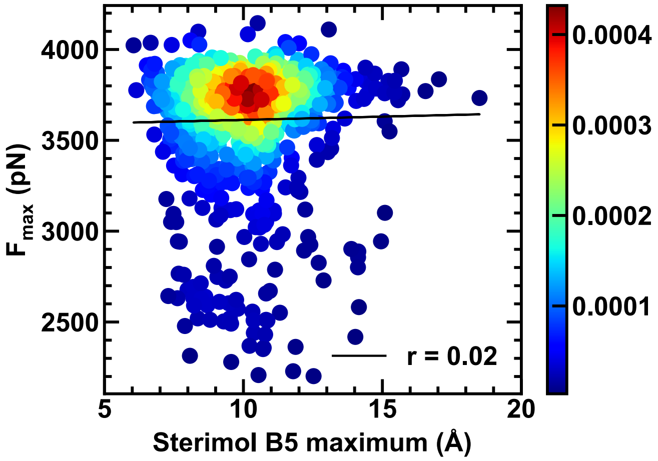


**Figure S19.** CoGEF simulated $F_{max}$ values as a function of the maximum of the B5 parameters calculated for each ligand. The B5 parameter describes the maximum width orthogonal to the ligand attachment to the ethyl group bond. Data points are colored by kernel density estimation (KDE) density values, as indicated by the color bar. A linear fit is shown as a black line.

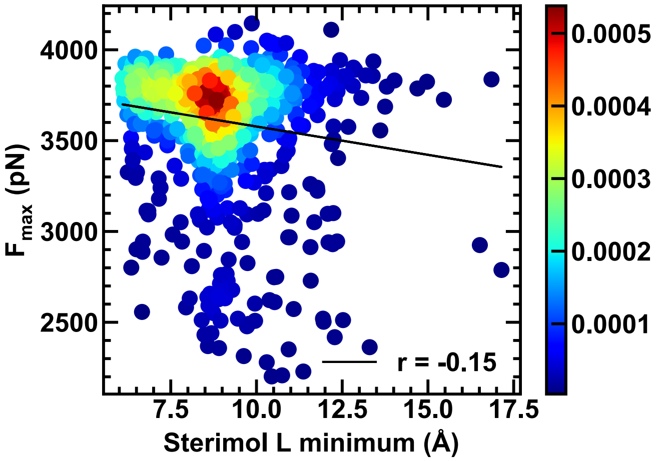


**Figure S20.** CoGEF simulated $F_{max}$ values as a function of the minimum of the L parameters calculated for each ligand. The L parameter describes the length parallel to the ligand attachment to the ethyl group bond. Data points are colored by kernel density estimation (KDE) density values, as indicated by the color bar. A linear fit is shown as a black line.

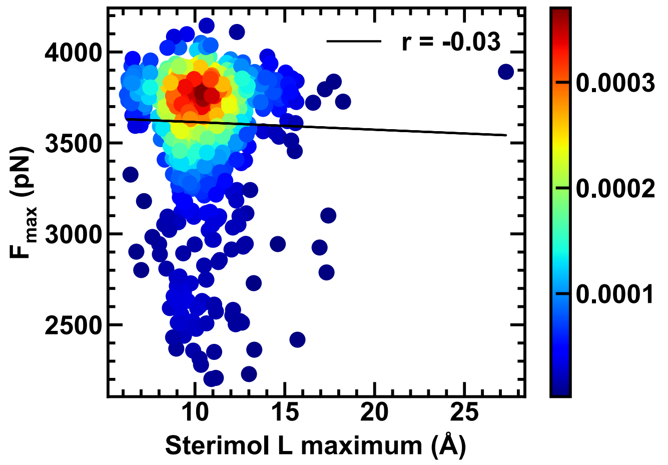


**Figure S21.** CoGEF simulated $F_{max}$ values as a function of the maximum of the L parameters calculated for each ligand. The L parameter describes the length parallel to the ligand attachment to the ethyl group bond.  Data points are colored by kernel density estimation (KDE) density values, as indicated by the color bar. A linear fit is shown as a black line.

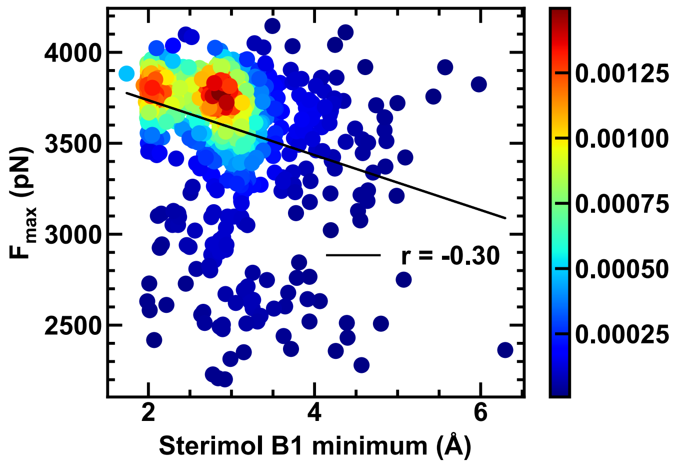


**Figure S22.** CoGEF simulated $F_{max}$ values as a function of the minimum of the B1 parameters calculated for each ligand. The B1 parameter describes the minimum width orthogonal to the ligand attachment to the ethyl group bond. Data points are colored by kernel density estimation (KDE) density values, as indicated by the color bar. A linear fit is shown as a black line.

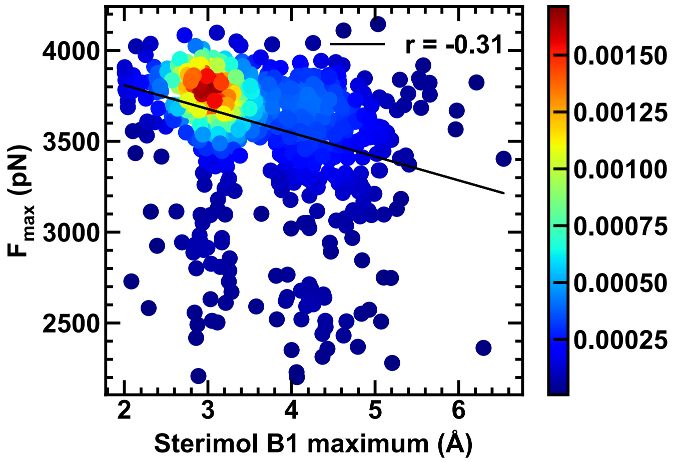


**Figure S23.** CoGEF simulated $F_{max}$ values as a function of the maximum of the B1 parameters calculated for each ligand. The B1 parameter describes the minimum width orthogonal to the ligand attachment to the ethyl group bond. Data points are colored by kernel density estimation (KDE) density values, as indicated by the color bar. A linear fit is shown as a black line.

**Table S2.** Pearson's correlation of different features with $F_{max}$.

| **Feature** | **Pearson's r** |
|---|---|
| Absolute dihedral | 0.38 |
| Ligand–ligand interaction energy | 0.28 |
| Absolute dihedral (NCI subset) | 0.84 |
| HOMO | −0.01 |
| LUMO | 0.01 |
| Dipole moment | −0.08 |
| Fe charge | 0.23 |
| SASA max | 0.35 |
| Sterimol L max | −0.03 |
| Sterimol L min | −0.15 |
| Sterimol B5 max | 0.02 |
| Sterimol B5 min | −0.02 |
| Sterimol B1 max | −0.31 |
| Sterimol B1 min | −0.30 |

**Text S7.** Feature selection for random forest models.

To train interpretable ML models, we used a random forest approach where feature importance can be obtained based on the Gini impurity. To avoid overfitting, we used recursive feature elimination cross-validation (RFECV), which resulted in the selection of 14 features for the classification task and 22 features for the regression task, both of which included the two newly introduced features (Supporting Information Tables S3–S4).

**Table S3.** Features selected for random forest classification task. MC refers to metal-centered RACs and APC refers to attachment point centered RACs. Delta refers to RACs that encode the differences of heuristic atom properties, as opposed to products for other RACs.

| Type | Property | Depth |
|---|---|---|
| Complex | $\chi$ | 5 |
| Complex | I | 5 |
| Complex | T | 4 |
| Ligand | $\chi$ | 3 |
| Ligand | Z | 5 |
| MC | T | 4 |
| MC | S | 4 |
| APC | $\chi$ | 5 |
| APC | T | 5 |
| APC | S | 5 |
| APC-delta | Z | 4 |
| APC-delta | S | 4 |
| 3D | Dihedral | N/A |
| 3D | NCI | N/A |

**Table S4.** Features selected for random forest regression task. MC refers to metal-centered RACs and APC refers to attachment point centered RACs. Delta refers to RACs that encode the differences of heuristic atom properties, as opposed to products for other RACs.

| **Type** | **Property** | **Depth** |
|---|---|---|
| Complex | Z | 3 |
| Complex | Z | 5 |
| Complex | T | 5 |
| Complex | S | 4 |
| Ligand | Z | 5 |
| MC | T | 4 |
| MC | S | 4 |
| MC-delta | $\chi$ | 4 |
| MC-delta | T | 4 |
| MC-delta | S | 3 |
| MC-delta | S | 5 |
| APC | $\chi$ | 4 |
| APC | $\chi$ | 5 |
| APC | T | 5 |
| APC | S | 5 |
| APC-delta | $\chi$ | 3 |
| APC-delta | $\chi$ | 4 |
| APC-delta | $\chi$ | 5 |
| APC-delta | Z | 4 |
| APC-delta | S | 4 |
| 3D | Dihedral | N/A |
| 3D | Dihedral | N/A |

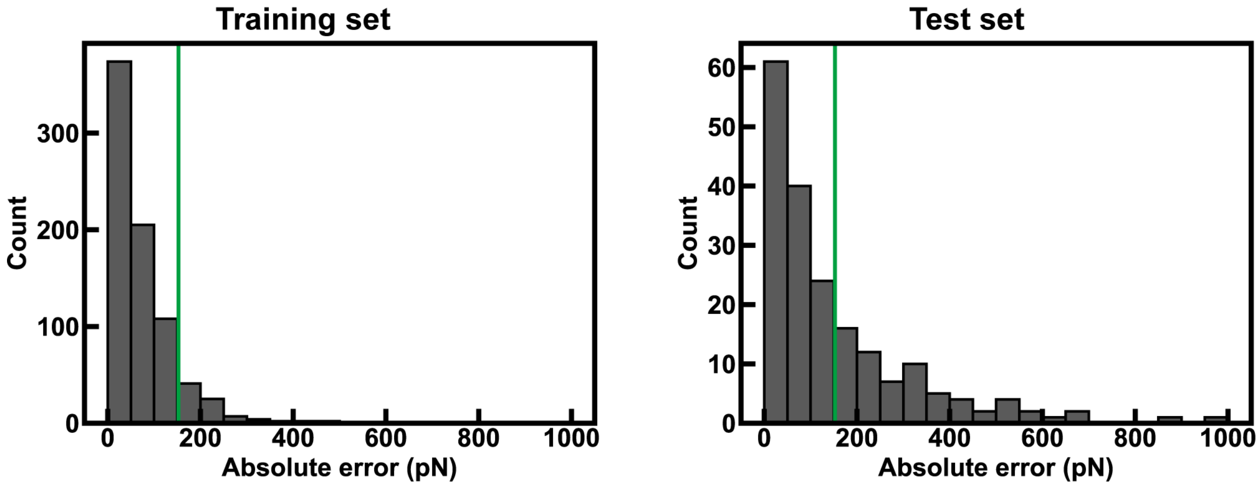


**Figure S24.** Distribution of training (left) and test (right) set errors for a random forest regression model of maximum force ($F_{max}$, bins of 50 pN), with the MAE annotated as a green vertical bar. Regression model was trained on the combination of randomly sampled and ML selected sets and contains both unbridged and bridged ferrocenes. The model was trained on the feature selected subset of RACs, AP dihedral angle and ligand–ligand interaction energy.

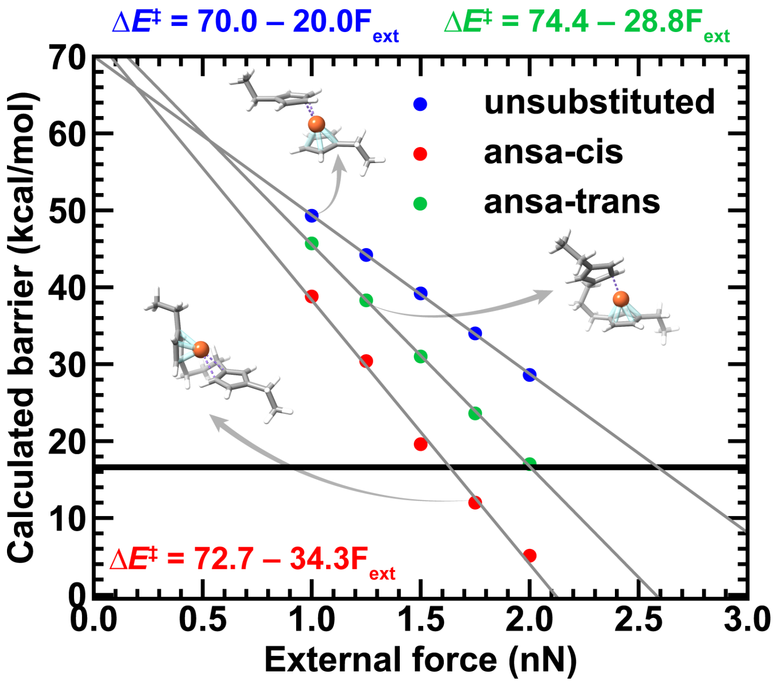


**Figure S25. Force-modified activation energies for unsubstituted and ansa-cis/trans ferrocenes.** Computed force-modified activation energies (in kcal/mol) versus external force (in nN) for the dissociation of unsubstituted, ansa-cis, and ansa-trans ferrocenes. Linear force response curves are shown as gray lines with associated linear fits as insets. The threshold energy of 16.6 kcal/mol that can be used to estimate single molecule force spectroscopy forces is shown as the bolded black line. Example transition state structures are shown as insets. Atoms are colored as follows: Fe in orange, C in gray, H in white. Coordination bonds are shown as hollow blue bonds and transition state bonds are shown as purple dashed lines.

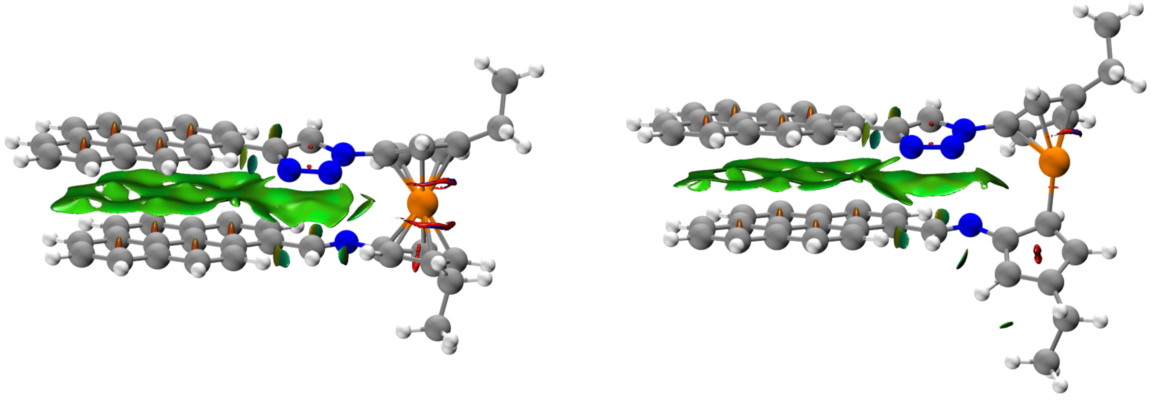

**EFILAI 1 nN GS** **EFILAI 1 nN TS**

**Figure S26. Non-covalent interaction analysis of EFILAI in the ground and transition states.** NCIPLOT analysis of **EFILAI** complex under 1 nN external applied force in the ground (GS) and transition (TS) states. Atoms are colored as follows: C in gray, H in white, N in blue, Fe in orange. The ligand–ligand interaction is 25 kcal/mol more favorable than the median computed over all studied ferrocenes.

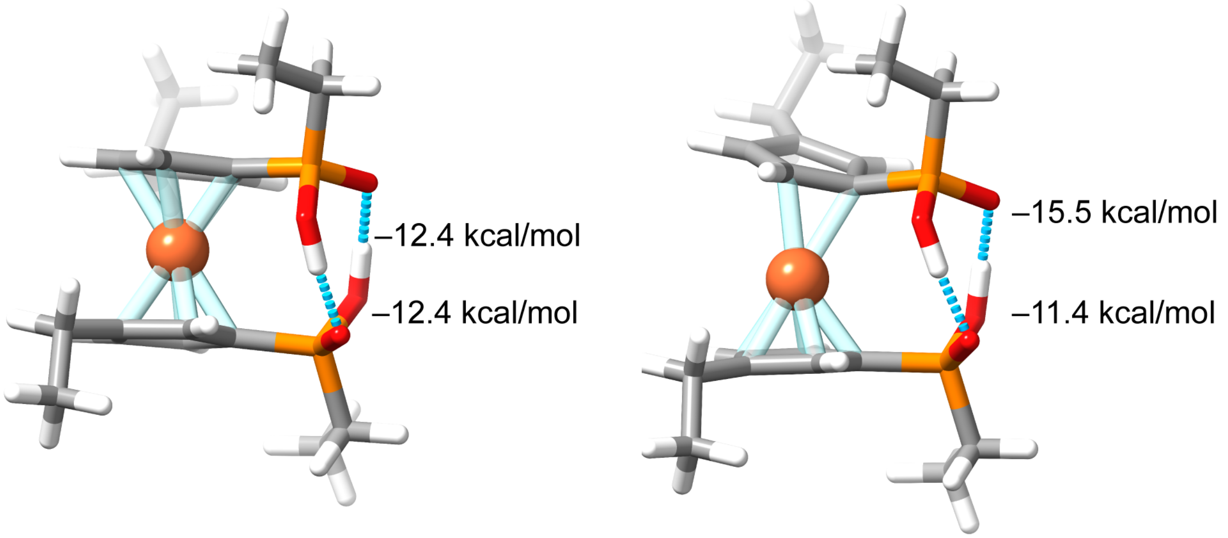


**EGIPER1 nN GS** **EGIPER1 nN TS**

**Figure S27. Hydrogen bonding analysis of EGIPER under force in the ground and transition states.** QTAIM hydrogen bonding analysis of **EGIPER** complex under 1 nN external applied force in the ground (GS) and transition (TS) states. Atoms are colored as follows: C in gray, H in white, Fe in brown, P in orange, O in red. Coordination bonds are shown as hollow blue lines and hydrogen bonds are shown as dashed blue lines. Hydrogen bonding strengths calculated using QTAIM for each bond are shown as labels. The ligand–ligand interaction is 14 kcal/mol more favorable than the median computed over all studied ferrocenes.

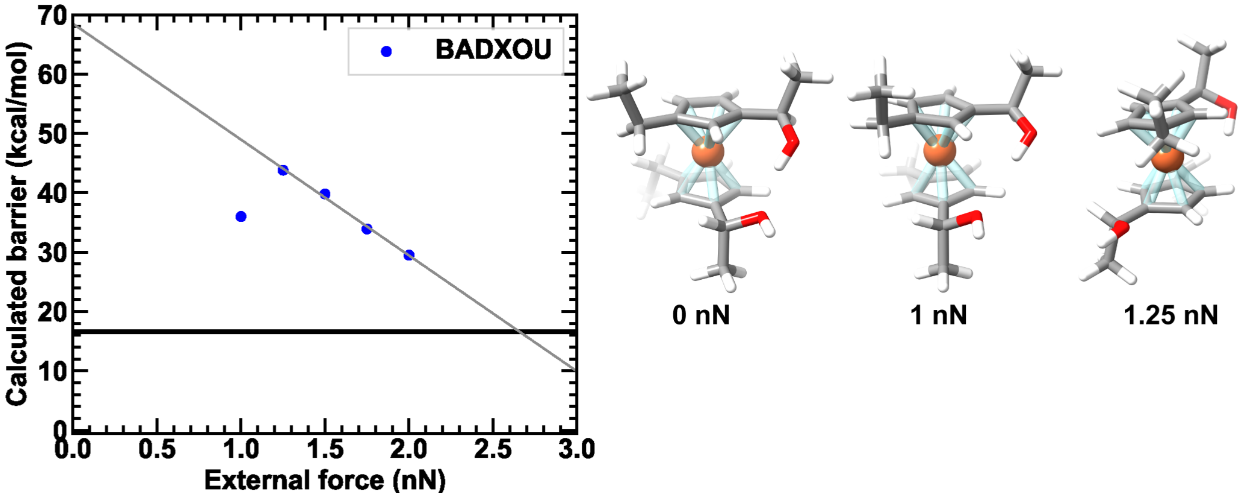


**Figure S28.** Computed force-modified activation energies (in kcal/mol) versus external force (in nN) for the dissociation of **BADXOU** (left) and ground state optimized structures of **BADXOU** complex under 0 nN, 1 nN and 1.25 nN external applied force. The linear force response curve is shown as a gray line. A linear fit was applied to activation energies calculated at 1.25 nN and above due to observed rotation at 1.25 nN. The threshold energy of 16.6 kcal/mol that can be used to estimate single molecule force spectroscopy forces is shown as the bolded black line.

**Table S5.** Energy decomposition analysis for the interaction energies. Decomposed energies correspond to the interaction energy between a Cp ligand and Fe bound to second Cp ligand. The change is computed as the difference in the interaction energy in the transition state geometry and in the ground state geometry. Calculations were done for structures optimized at 1.5 nN external force. $\Delta E_{dist}$ – distortion energy, $\Delta E_{elec}$ – electrostatic interaction energy, $\Delta E_{Pauli}$ – Pauli repulsion, $\Delta E_{disp}$ – dispersion interaction energy, $\Delta E_{pol}$ – intrafragmental orbital relaxation energy, $\Delta E_{CT}$ – interfragmental orbital relaxation energy. All energies are provided in kcal/mol.

| Complex | $\Delta E_{dist}$ | $\Delta E_{elec}$ | $\Delta E_{Pauli}$ | $\Delta E_{disp}$ | $\Delta E_{pol}$ | $\Delta E_{CT}$ |
|---|---|---|---|---|---|---|
| Ferrocene | 1.4 | 49.6 | −80.4 | 11.2 | 20.1 | 68.7 |
| m-TMS-Fc | 0.3 | 35.9 | −64.9 | 8.9 | 17.1 | 66.7 |
| o-TMS-Fc | 0.0 | 43.5 | −73.4 | 12.4 | 19.2 | 66.9 |

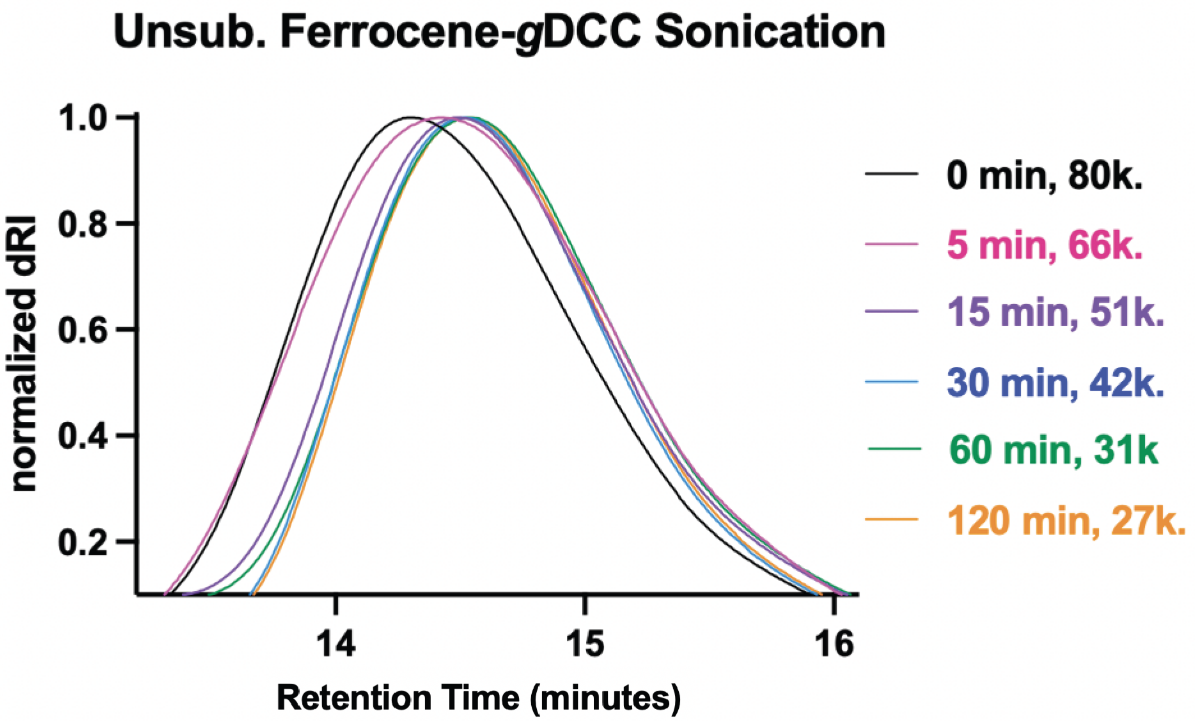


**Figure S29.** Size-exclusion chromatography overlay of sonicated polymers containing unsubstituted ferrocene. One full scission cycle is achieved at roughly 30 min.

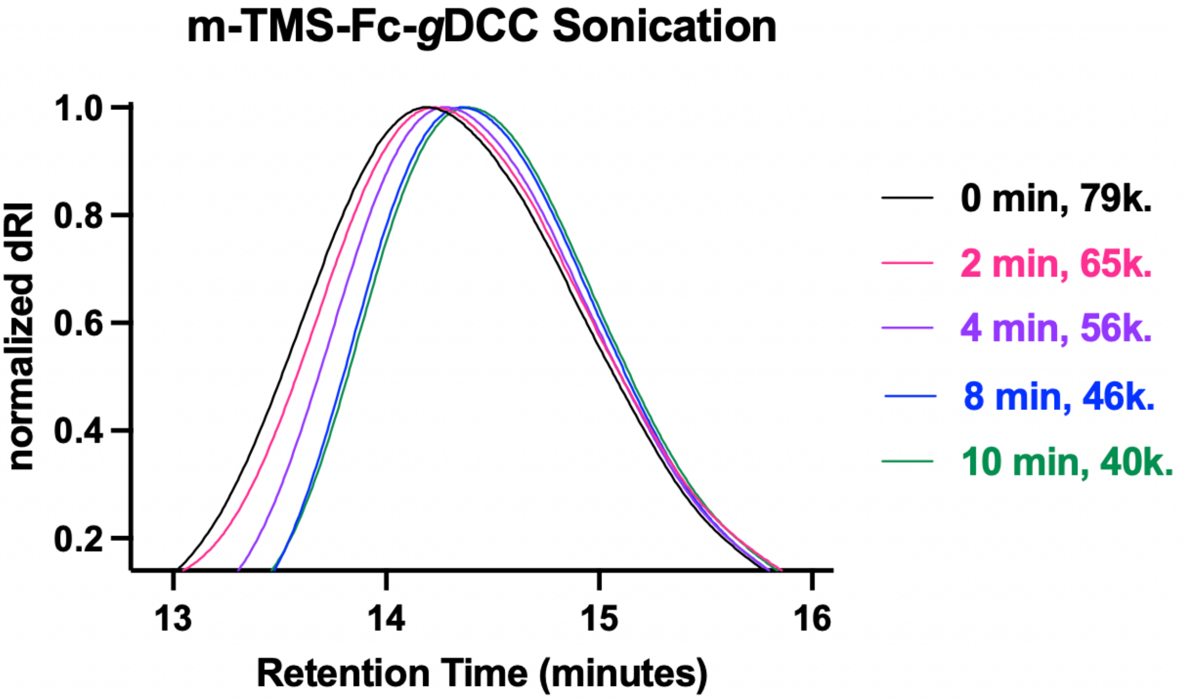


**Figure S30.** Size-exclusion chromatography overlay of sonicated polymers containing m-TMS-Fc. One full scission cycle is achieved at roughly 10 min.

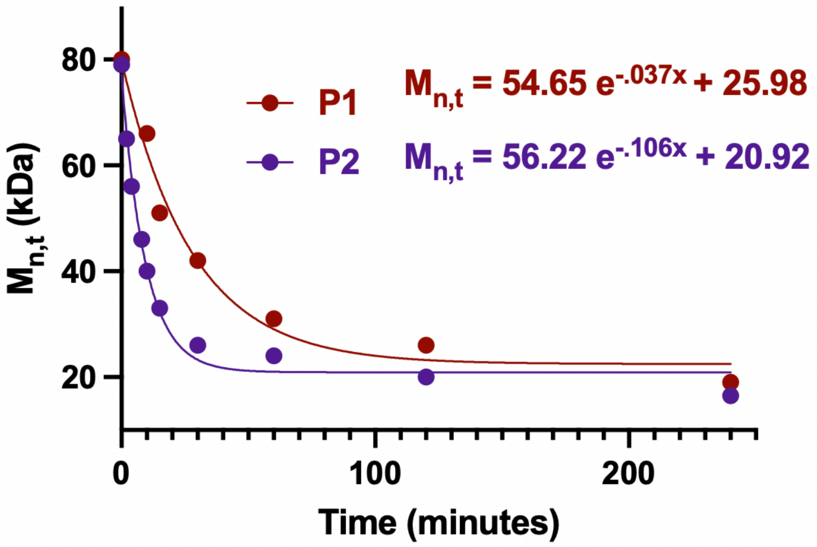


**Figure S31.** First-order exponential decay in $M_{n,t}$ as a function of sonication time. Where **P1** represents the ferrocene-containing polymer, and **P2** the m-TMS-Fc-containing polymer. The change in $M_{n,t}$ with sonication time t is fit to a first-order exponential decay ($dM_{n,t}/dt = k_d(M_{n,t} - M_{lim})$). Rate constants $k_d$ of ferrocene bond scissions of **P1** and **P2** are $3.7*10^{-2}$ $min^{1}$ and $1.1*10^{-1}$ $min^{1}$, respectively.

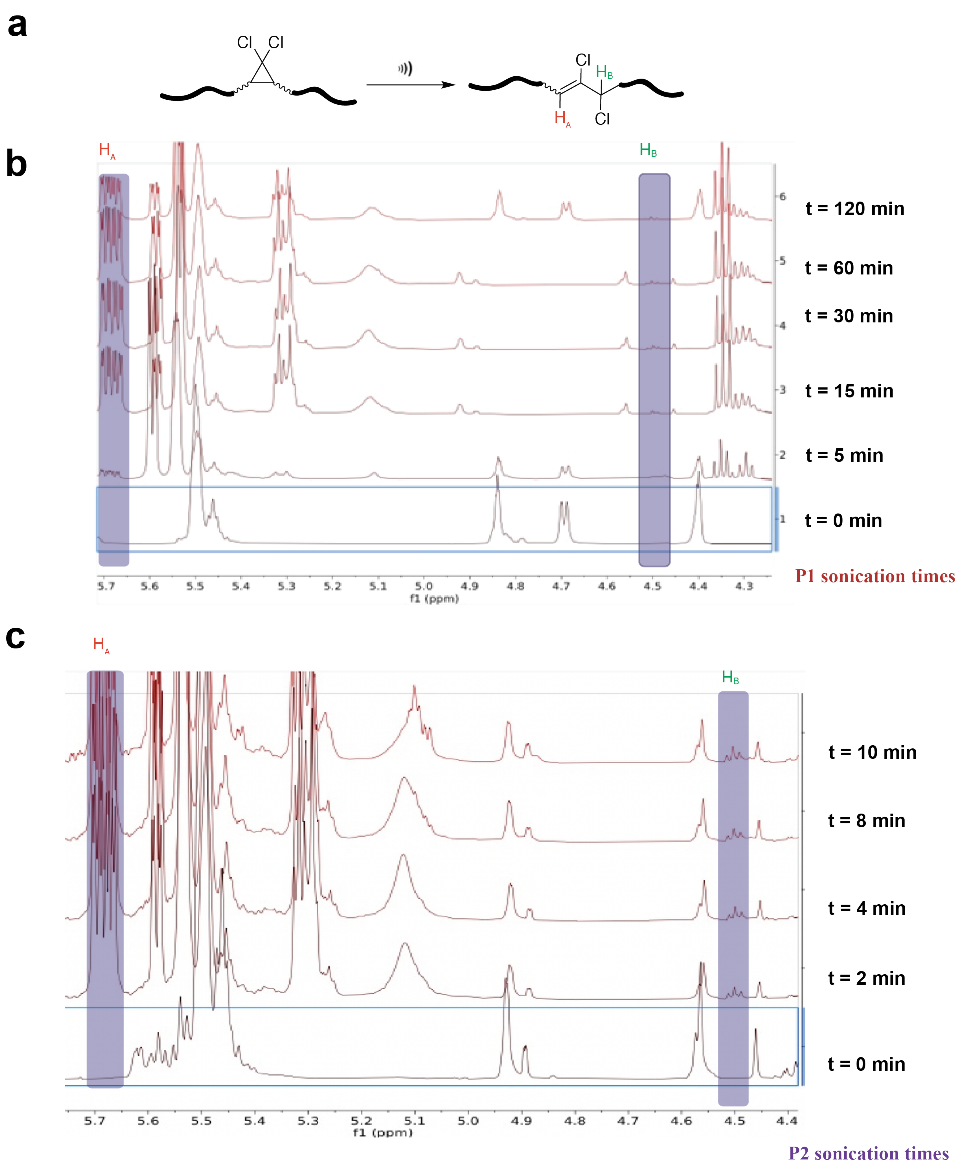


**Figure S32. a,** Chemical schematic of gDCC along a polymer strand undergoing mechanical activation by pulsed ultrasound to open to a 2,3-dichloroalkene. **b,** stacked HNMR spectra of P1 undergoing sonication at various time points. **c,** stacked HNMR spectra of **P2** undergoing sonication at various time points. Characteristic resonances for $H_a$ and $H_b$ are highlighted in violet boxes, and calculated as per previous methods.[12]

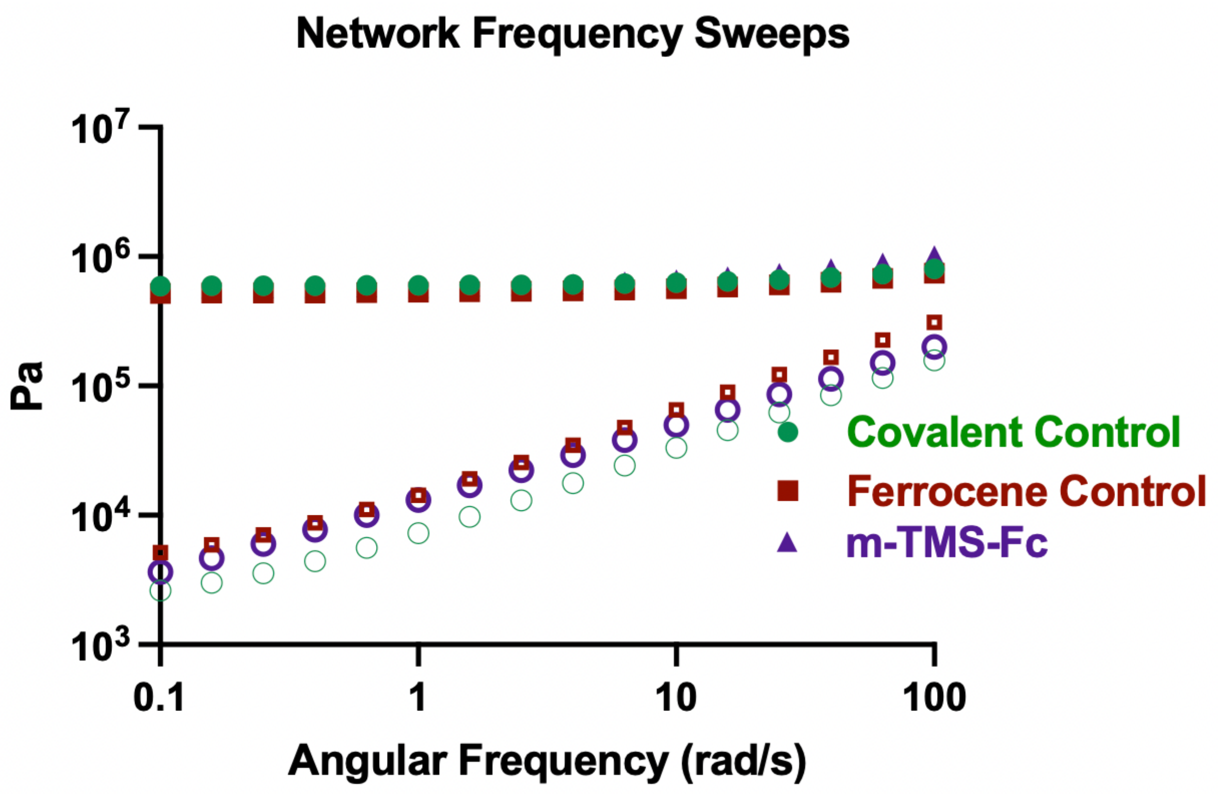


**Figure S33.** Rheological frequency sweep of elastomers formed as described in Text S11, 0.1–100 rad/s. Filled circles represent storage modulus, hollow symbols represent loss modulus. Moduli are shown in units of pascals.

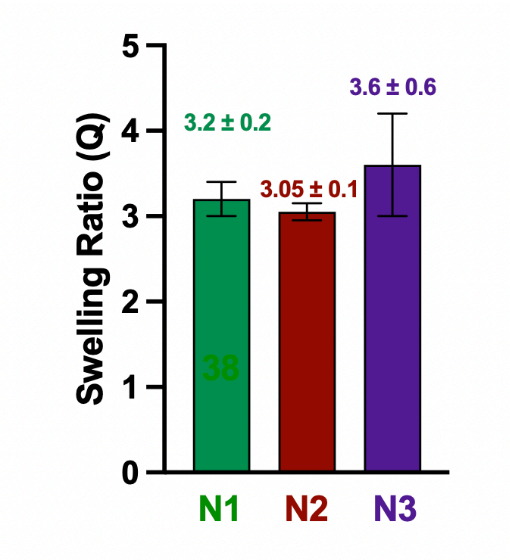


**Figure S34.** Swelling ratio (Q) by mass. Q = [(Weight swollen – Weight dried) / (Weight dried)]. N1 – network with butanediol diacrylate crosslinker, N2 – network with ferrocene diacrylate crosslinker, N3 – network with m-TMS-Fc crosslinker. Networks made as per Text S11.

**Text S8.** Synthesis of networks containing ferrocene crosslinkers.

Butanediol diacrylate was used as purchased. Ferrocene crosslinkers were purified by column chromatography, dried under rotary evaporation, and dried on a Schlenk line for five minutes before being immediately used for network synthesis. Butyl acrylate and butanediol diacrylate were passed through short alumina columns to remove inhibitor. Stoichiometric ratios of [Butyl acrylate]:[Crosslinker]:[Chain Transfer Agent]:[Azobisisobutyronitrile (AIBN)] were [1]:[85]:[1400]:[2000].

To a flame-dried 40 mL scintillation flask, 12 mL of butyl acrylate (105.2 mmol), 30.3 mg 4-Cyano-4-(((dodecylthio)carbonothioyl)thio)pentanoic acid (0.075 mmol), 8.6 mg AIBN (0.0526) and .1 mL of DMF were homogenized and equally portioned into three separate, 20 mL scintillation flasks containing stoichiometric equivalents of crosslinkers. These solutions were sparged with nitrogen for 4 minutes and transferred to Teflon molds covered by a glass lid inside an oven at 70°C under nitrogen flow, and left to cure for 24 hours. The elastomers were then removed from the Teflon molds onto separate surfaces, dried under vacuum overnight, and cut into appropriate shapes for testing as per previously reported tear testing and rheological methodologies.[14]

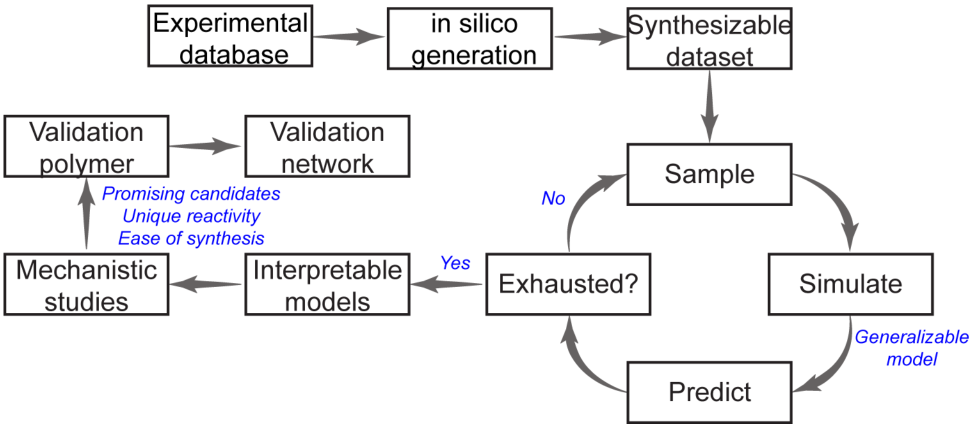


**Figure S35.** A workflow used for the discovery of new mechanophores.

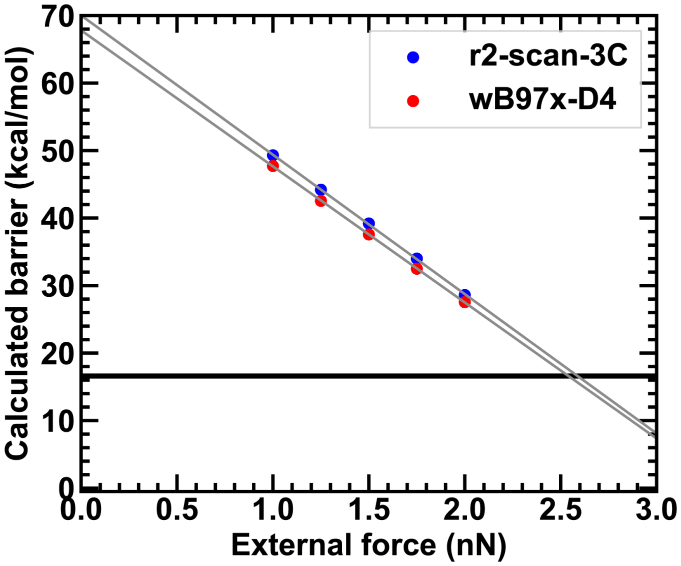


**Figure S36.** Computed force-modified activation energies (in kcal/mol) versus external force (in nN) for the unsubstituted ferrocene, using the $r^2$SCAN-3C functional and the ωB97x-D4 functional. For the ωB97x-D4 functional, optimization was carried out using def2-SVP basis set and single-point energy calculations were carried out using the def2-TZVP basis set. All calculations employed implicit solvent model C-PCM with a dielectric of ε = 7.25 to model the experimental THF solvent.

**Table S6.** Calculated vertical spin-splitting energies for m-TMS-Fc under tension. All calculations, besides r2scan-3c, utilized def2-TZVP basis set, and were carried out with implicit solvation in THF using CPCM.

| Method | r2scan-3c | B3LYP | M06 | PBE | PBE0 | ωB97x-D3 |
|---|---|---|---|---|---|---|
| 1 nN | 55.7 | 51.4 | 39.2 | 72.8 | 42.7 | 49.3 |
| 2 nN | 43.3 | 39.7 | 27.3 | 60.9 | 30.7 | 37.6 |

**Table S7.** Set of hyperparameter choices for ANN models.

| Hyperparameter | Search Space | Gen 0 model | Gen 1 model |
|---|---|---|---|
| **Learning rate** | uniform(1e-5, 1e-3) | 0.00054918541 | 0.0006420198 |
| **Dropout rate** | uniform(0, 0.5) | 0.48699029062 | 0.1119184915 |
| **Regularization** | loguniform(log(1e-5), log(1) | 7.275799543 e-5 | 0.1020764889 |
| **Batch size** | [16, 32, 64, 128, 256] | 256 | 64 |
| **Hidden layer** | [(128, 128), (256, 256), (512, 512), (128, 128, 128), (256, 256, 256), (512, 512, 512)] | (512,512) | (512,512) |
| **Beta_1** | uniform(0.80, 0.99) | 0.87074004895 | 0.8989555783 |
| **Decay** | loguniform(log(1e-5), log(1) | 0.00089562619 | 2.201310427 e-5 |
| **Residual layer** | True, False | False | True |
| **Bypass** | True, False | False | True |

**Table S8.** Set of hyperparameter choices for RF models.

| Hyperparameter | Search Space | Classification | Regression |
|---|---|---|---|
| **Max depth** | 3, 4, 5, 6, 7, 8, 9 | 9 | 9 |
| **Max features** | log2, sqrt | sqrt | log2 |
| **Bootstrap** | True, False | True | False |
| **n_estimators** | 100,200,300,400,500,600,700,800,900,1000 | 800 | 700 |
| **min_samples_split** | 2, 3, 4, 5 | 2 | 4 |

**Text S9. General Experimental Details.**

**Materials**

All commercially available compounds (ferrocene, butyllithium solution, trimethylsilyl chloride, hydroxyethyl acrylate, butyl acrylate, butanediol diacrylate, RAFT agent, AIBN) were purchased from Sigma-Aldrich and used as received, and novel compounds were made with commercial precursors from Sigma. $CO_2$ (99.999%) was purchased from Airgas.

**Characterization and methods**

$^1H$ NMR and $^{13}C$ NMR spectra were recorded on a 400 MHz or 500 MHz Varian NMR spectrometer using $CDCl_3$ as solvent. The chemical shifts are reported with respect to $CHCl_3/CDCl_3$($\delta$(1H) = 7.26 ppm, $\delta$(13C) = 77.0 ppm). ESI-MS spectra were collected on an Agilent LC/MSD Trap instrument.

Pulsed ultrasound experiments were conducted on Vibracell Model VCX50 sonicator at 20 kHz with a 12.8 mm replaceable tip titanium probe from Sonics and Materials. Sonication was peformed on 2 mg/mL polymer solutions in THF immersed in an ice bath. The solutions were sparged with nitrogen for 30 min before sonication and a continuous $N_2$ stream was bubbled in the system during sonication. Pulsed ultrasound was performed at a power of 8.7 W/cm$^2$. The sonication sequence was set as 1s on, 1s off.

Rheological measurements were conducted on an Anton Paar MCR 302 rheometer with an 8 mm parallel plate geometry. Uniaxial tensile tests and tearing energy measurements were performed on a TA Instruments RSA III Dynamic Mechanical Analyzer (force resolution: 0.0001 N, displacement resolution: 1 μm), courtesy of Duke University's Shared Material Instrument Facility (SMIF).

**Text S10. Small Molecule Synthesis.**

1,1′-Bis(trimethylsilyl)ferrocene[10], DPTS[11], and unsubstituted ferrocene dienes and macrocycles[12], and 10-hydroxydecyl acrylate[13] were synthesized as previously reported.

Synthesis of 1,1′-Bis(trimethylsilyl)ferrocene dicarboxylic acid **(1)**.

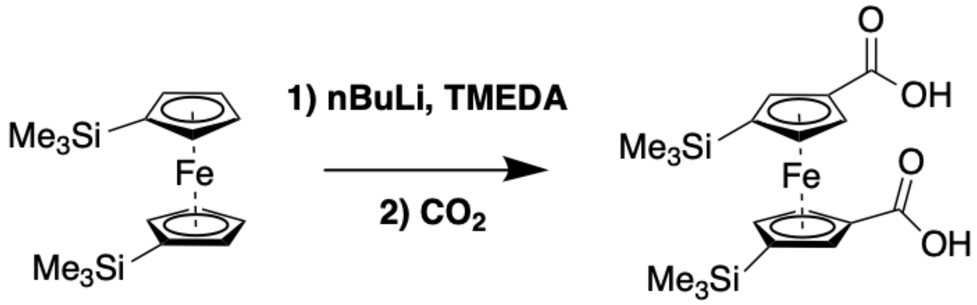


**1**

To a flame-dried 100 mL round bottom flask and stir bar was charged 1,1′-Bis(trimethylsilyl)ferrocene (2 grams, 6.06 mmol), dry hexanes (10 mL), TMEDA (4.6 mL, 30 mmol), n-BuLi (12.2 mL, 2.5 M solution, 30 mmol) under nitrogen. The mixture was allowed to stir for 24 hours before cooling to –78 °C. An outlet needle was inserted in the septum, and $CO_2$ gas was sparged in the solution for 90 – 120 minutes. A color change from red to yellow occurred instantly. If necessary, additional aliquots of dry hexane were added to the mixture. The reaction was then carefully quenched with 20 mL deionized water and rinsed with benzene, and the organic layer was rinsed with water washes until they ran clear. The combined water layer was precipitated with a copious amount of 1M HCl solution, collected by filtration, dried, purified by column chromatography (dichloromethane:methanol = 95:5), and again by recrystallization in a solution of acetone with drops of methanol to yield **1** in 50-65% yield. $^1$H NMR (500 MHz, $CDCl_3$): δ 5.07 (s, 1H), 4.89 (s, 1H), 4.31 (s, 1H), 0.27 (s, 18H). $^{13}$C NMR (126 MHz, $CDCl_3$) δ 175.747, 79.92, 76.60, 73.64, 0.0 (TMS).

Synthesis of 1,1′-Bis(trimethylsilyl)ferrocene diene **(2)**.

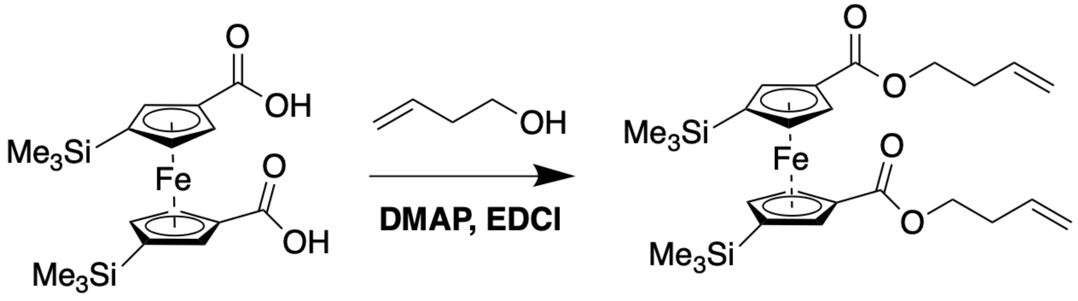


To a 250 mL round bottom flask and stir bar was charged **1** (500 mg, 1.2 mmol), DMAP (244 mg, 2.4 mmol), EDC·HCl (1.4 g, 7.2 mmol), and 80 mL of dichloromethane. The reaction was stirred for five minutes and then charged with but-3-en-1-ol (520 mg, 7.2 mmol) and left to stir for two days. 50 mL of water was then added to the mixture, the organic layer extracted, and the aqueous layer washed with dichloromethane until it ran clear and used directly in the next step.

Synthesis of 1,1′-Bis(trimethylsilyl)ferrocene macrocycle **(3)**.

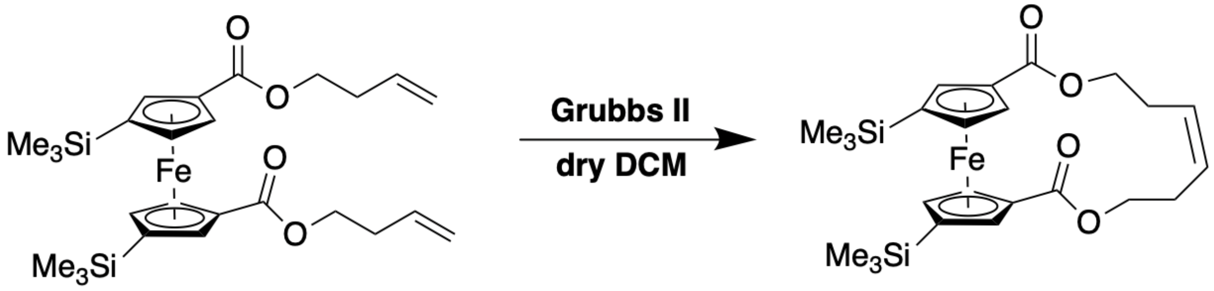


To a 250 mL round bottom flask and stir bar was charged **2** (116 mg, 1.2 mmol), Grubbs II catalyst (18 mg, 21.2 mmol) and 120 mL of dry dichloromethane. The septum was secured with several wrappings of parafilm and the reaction was stirred for ten hours at 45 °C, followed by quenching with a small amount of ethyl vinyl ether and concentration onto silica. The mixture was purified by flash column chromatography (hexanes:ethyl acetate = 7:3) to afford macrocycle **3** (27 mg, 25% yield). $^{1}$H NMR (500 MHz, $CDCl_3$): δ 5.62 (m 2H), 4.89 (dd, 2H), 4.21-4.62 (m, 6H), 4.08

(dt, 2H), 0.28 (s, 18H). $^{13}C$ NMR (126 MHz, $CDCl_3$) δ 170.16, 131.048, 72.39, 59.294, 0.0 (TMS). $[M + H]^+$ calculated for $C_{24}H_{34}O_4Si_2{}^{56}Fe$: 499.1418; observed 499.1417. $[M + Na]^+$ calculated 521.1237; observed 521.1241.

Synthesis of ferrocene diacrylate **(4)**.

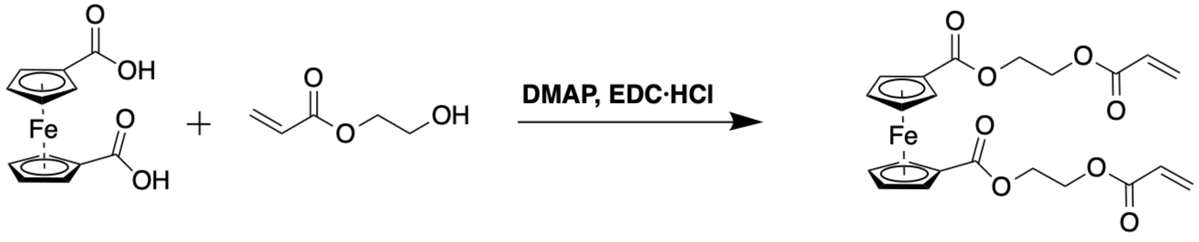


4

To a 500 mL round bottom flask and stir bar was charged 1,1'-Ferrocenedicarboxylic acid

(500 mg, 1.8 mmol), DMAP (440 mg, 3.6 mmol), EDC·HCl (2.1 g, 10.8 mmol), and 350 mL of dichloromethane. The reaction was stirred for five minutes and then charged with 2-hydroxyethylacrylate (1.24 ml, 10.8 mmol) and left to stir for two days. 50 mL of water was then added to the mixture, the organic layer extracted, and the aqueous layer washed with dichloromethane until it ran clear. The combined organic layers were then dried with sodium sulfate and concentrated onto silica. The mixture was purified by flash column chromatography (hexanes:ethyl acetate = 95:5) to afford diacrylate **4** (610 mg, 71% yield). $^1H$ NMR (500 MHz, $CDCl_3$): δ 6.46 (ddd, 2H), 6.19 (ddd, 2H), 5.89 (ddd, 2H), 4.84 (t, 4H), 4.42-4.52 (m, 8H), 4.28-4.42 (m, 2H), 3.7-4.15 (m, 2H). $^{13}C$ NMR (126 MHz, $CDCl_3$) δ 170.217, 166.481, 131.35, 128.06, 73.07, 71.717, 66.16, 61.04, 14.16. $[M + H]^+$ calculated for $C_{22}H_{22}O_8{}^{56}Fe$: 471.0737; observed 471.0738. $[M + Na]^+$ calculated 493.0556; observed 493.0562.

Synthesis of 1,1′-Bis(trimethylsilyl)ferrocene diacrylate **(5)**.

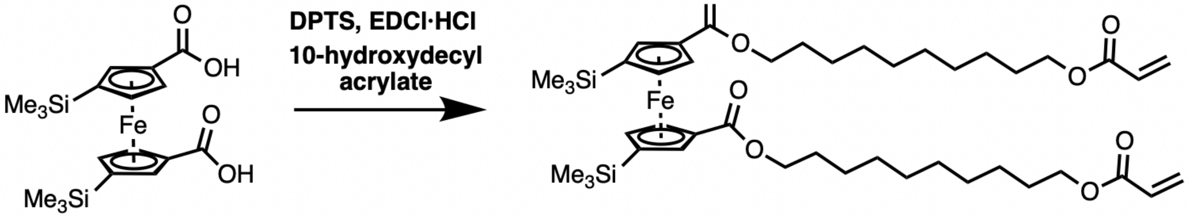


5

To a 250 mL round bottom flask and stir bar was charged **1** (500 mg, 1.2 mmol), DPTS (700 mg, 2.4 mmol), EDC·HCl (1.4 g, 7.2 mmol), and 150 mL of dichloromethane. The reaction was stirred for five minutes and then charged with 10-hydroxydecyl acrylate[4] (1.3 ml, 7.2 mmol) and stirred for 60 hours. 50 mL of water was then added to the mixture, the organic layer extracted, and the aqueous layer washed with dichloromethane until it ran clear. The combined organic layers were then dried with sodium sulfate and concentrated onto silica. The mixture was purified by flash column chromatography (hexanes:ethyl acetate = 80:20) to afford diacrylate **5** (427 mg, 55% yield). $^{1}H$ NMR (500 MHz, $CDCl_3$): δ 6.37 (dd, 2H), 6.14 (dd, 2H), 5.81 (dd, 2H), 4.92 (dd, 2H), 4.51 (m, 2H), 4.27 (m, 2H), 4.14 (m, 8H), 1.22-1.42 (m, 32H), 0.27 (s, 18H). $^{13}C$ NMR (126 MHz, $CDCl_3$) δ 170.522, 166.75, 130.84, 129.08, 65.119, 32.01, 29.923, 23.074, 14.542, 0.002 (TMS). $[M + H]^+$ calculated for $C_{44}H_{70}O_8{}^{56}FeSi_2$: 839.4031; observed 839.4026. $[M + Na]^+$ calculated 856.4297; observed 856.4297. $[M + K]^+$ calculated 861.3851, observed 861.3849.

A bis(trimethylsilyl)ferrocene diacrylate made with 2-hydroxyethyl acrylate was initially used to probe the toughening effect of m-TMS-Fc. However, the compound was found to be volatile enough to significantly increase the sol fraction and decrease the gel yield. To ameliorate this, longer alkyl handles were used.

**Text S11.** Synthesis of ferrocene-containing polymers.

To a flame dried, 5 mL RBF with a magnetic stir bar was charged **3** (29 mg, .058 mmol, 1 eq), GDCC-cyclooctene (99 mg, 0.524 mmol, 9 eq), 0.4 mL dry dichloromethane, and GII catalyst (0.62 mg, 0.00074 mmol, 1/150 eq). The vial was stirred for 24-48 hours, quenched with ethyl vinyl ether, and precipitated twice with a mixture of 90/10 methanol/water and dried overnight on a Schlenk line. The unsubstituted ferrocene was made by a similar procedure, as previously reported.

**NMR Spectra.**

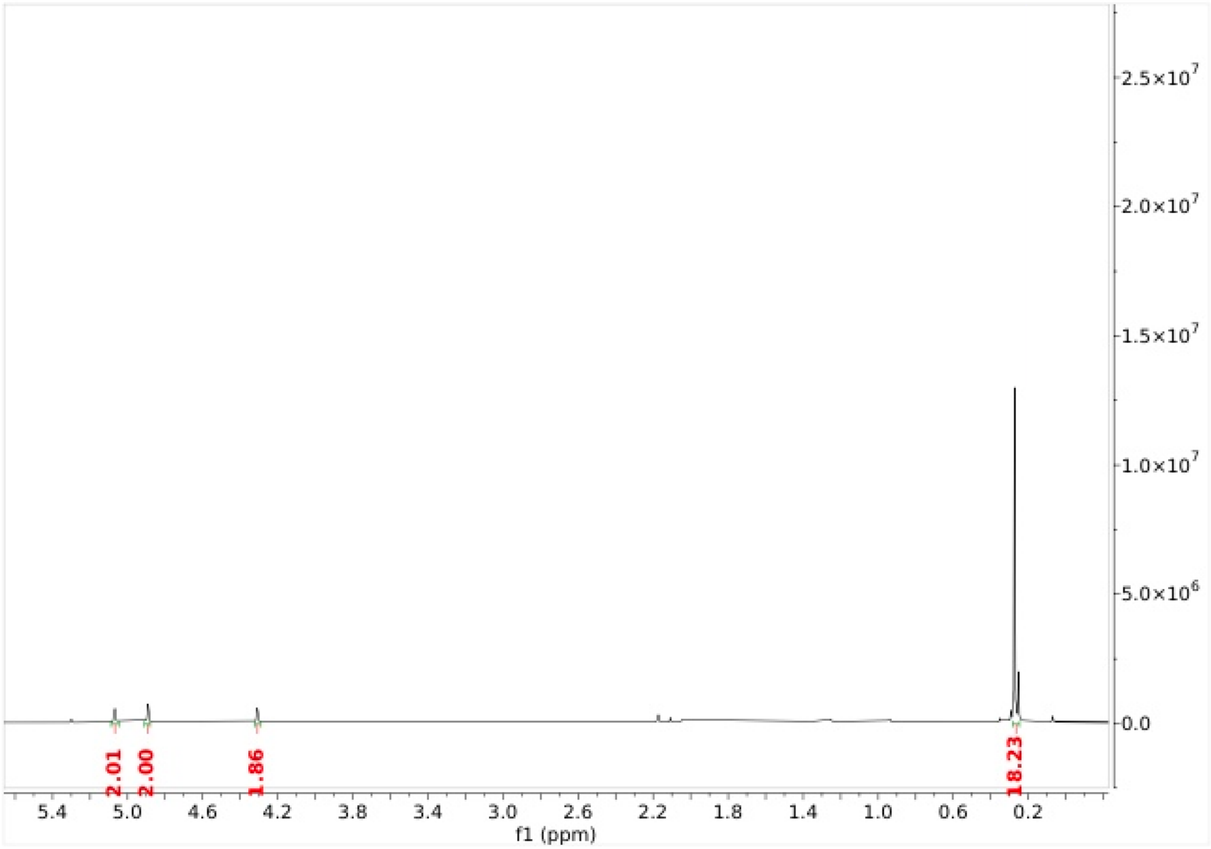


**Figure S37.** 1,1′-Bis(trimethylsilyl)ferrocene dicarboxylic acid **(1)** HNMR.

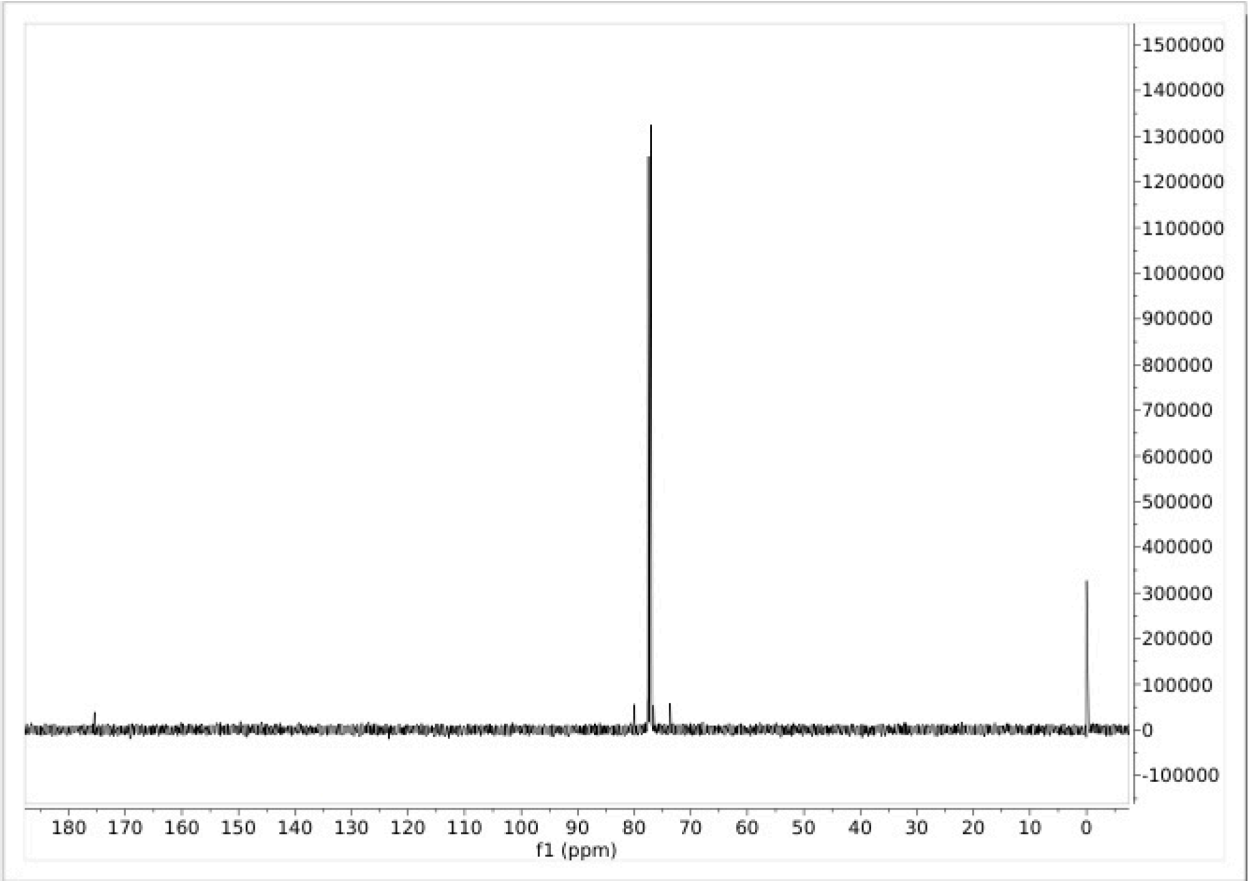


**Figure S38.** 1,1′-Bis(trimethylsilyl)ferrocene dicarboxylic acid **(1)** CNMR.

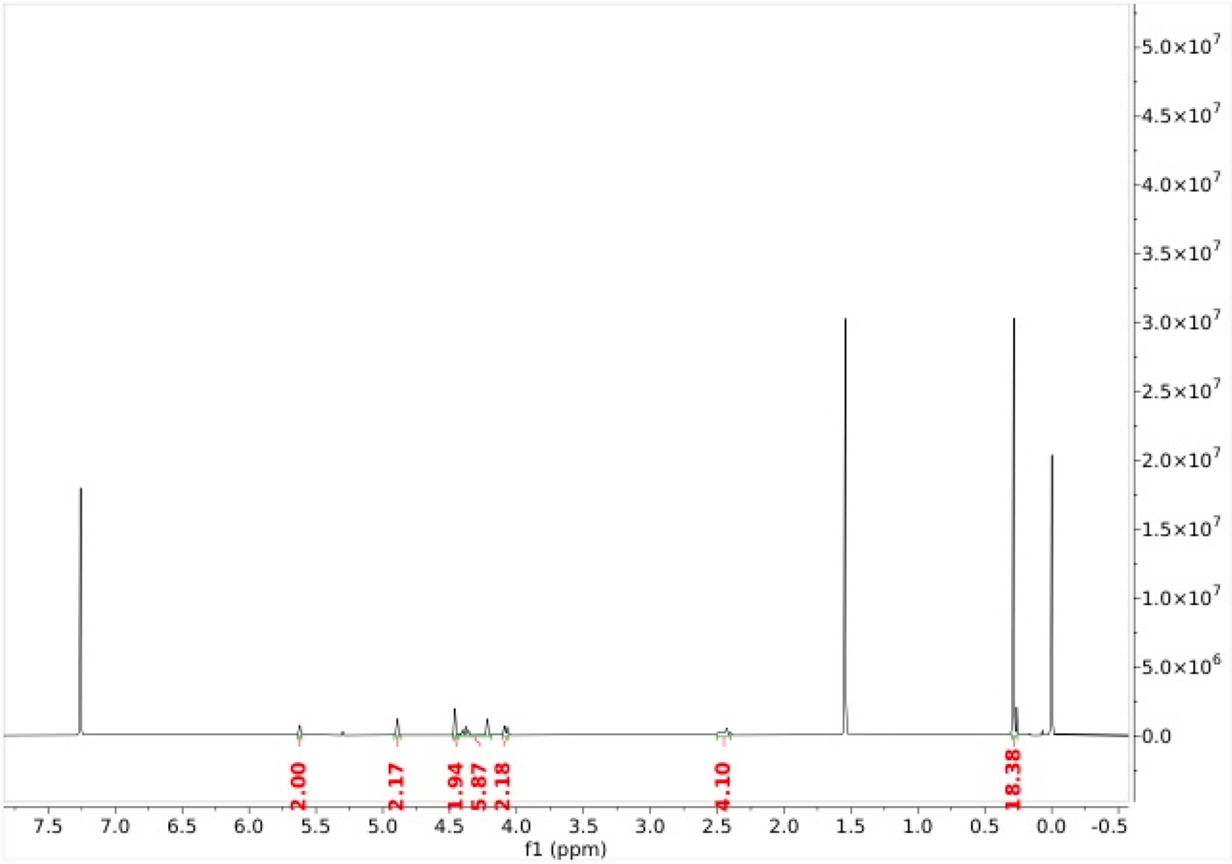


**Figure S39.** 1,1′-Bis(trimethylsilyl)ferrocene macrocycle **(3)** HNMR.

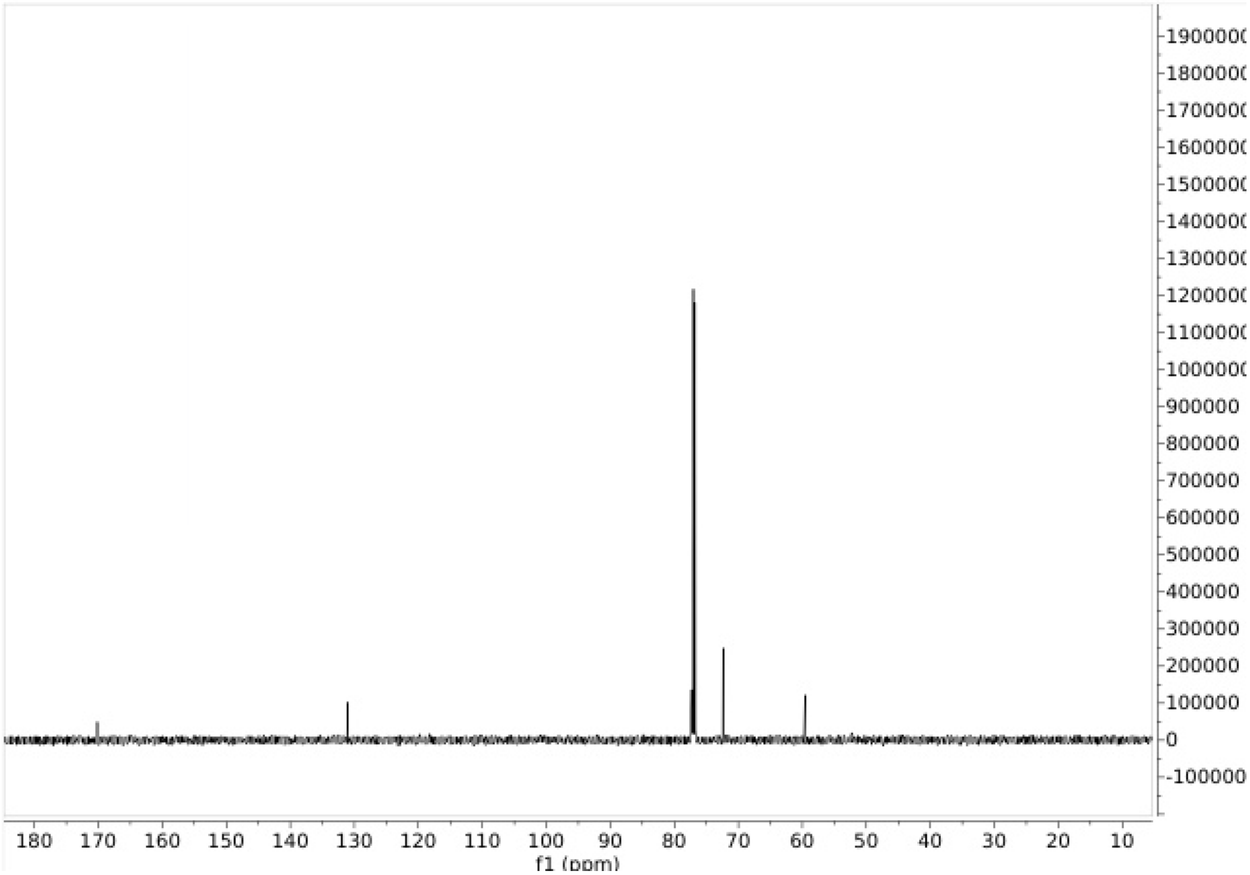


**Figure S40.** 1,1′-Bis(trimethylsilyl)ferrocene macrocycle **(3)** CNMR.

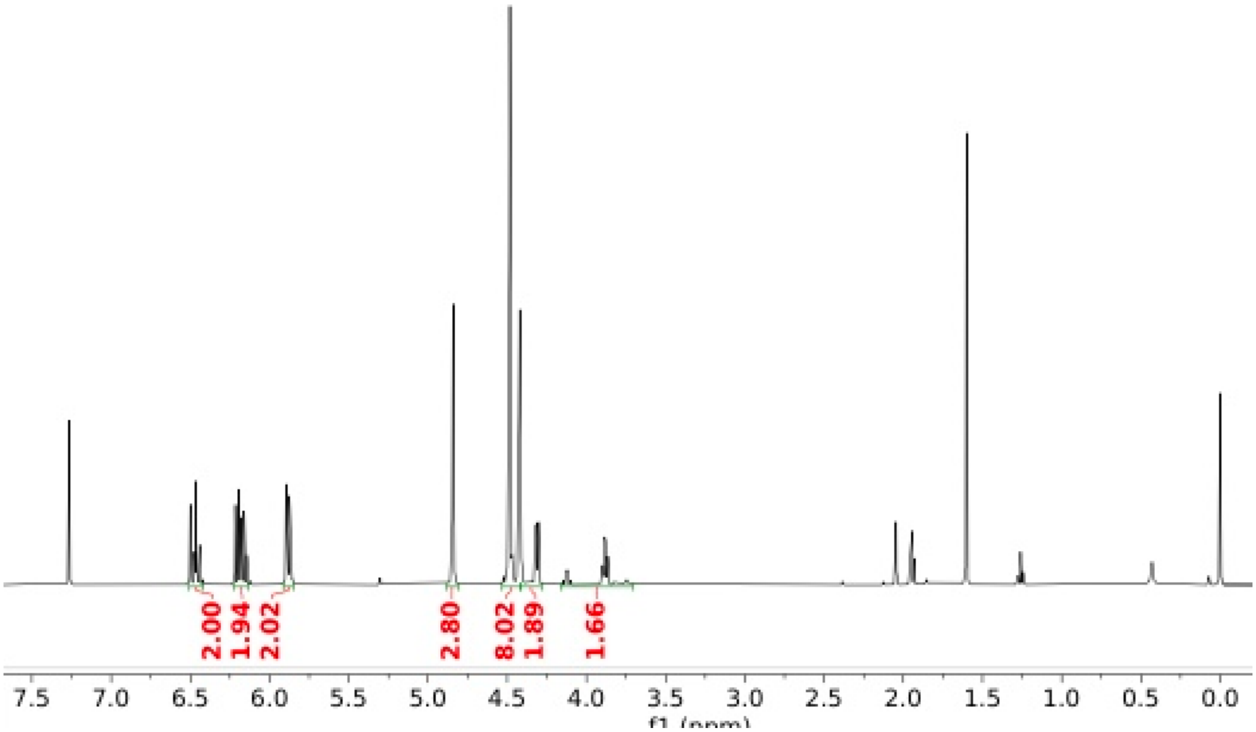


**Figure S41.** Ferrocene diacrylate **(4)** HNMR.

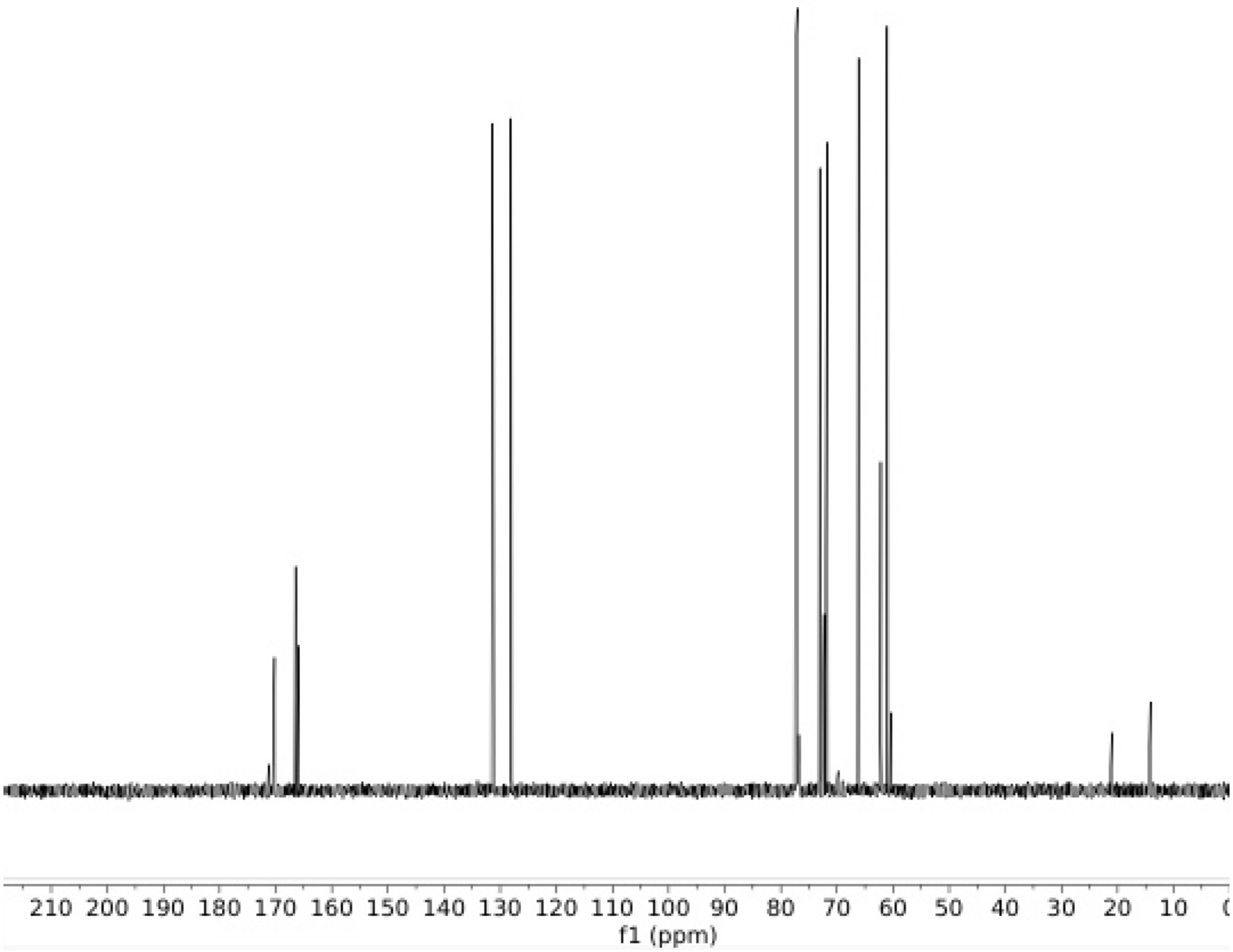


**Figure S42.** Ferrocene diacrylate **(4)** CNMR

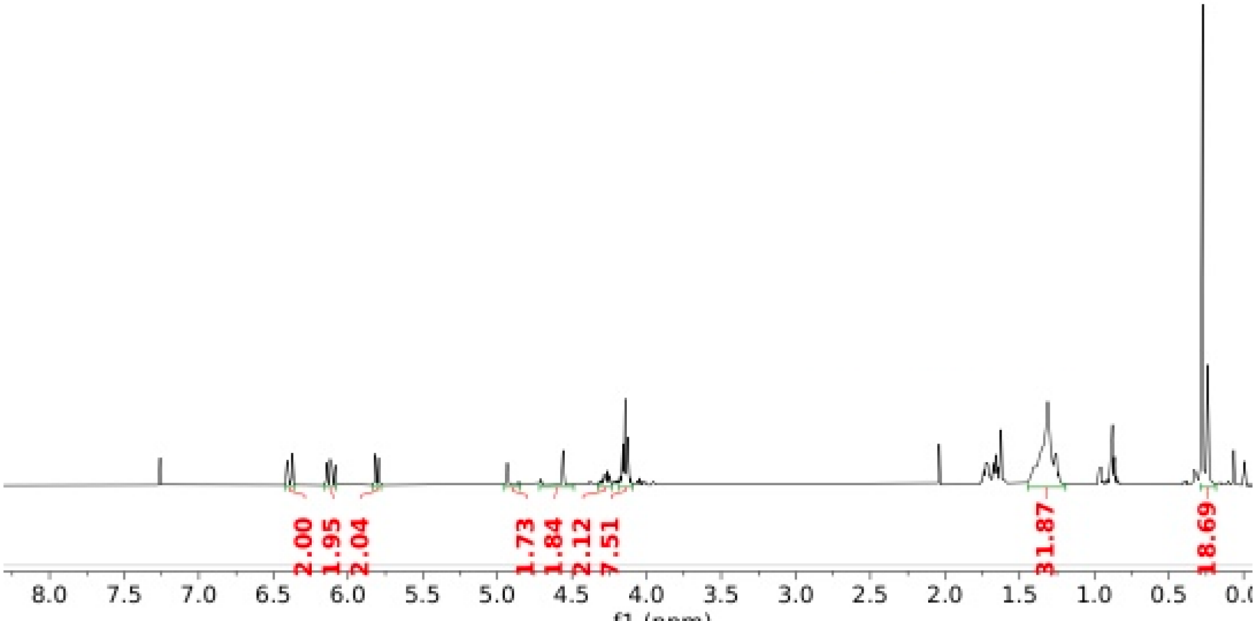


**Figure S43.** m-TMS-Fc diacrylate **(5)** HNMR.

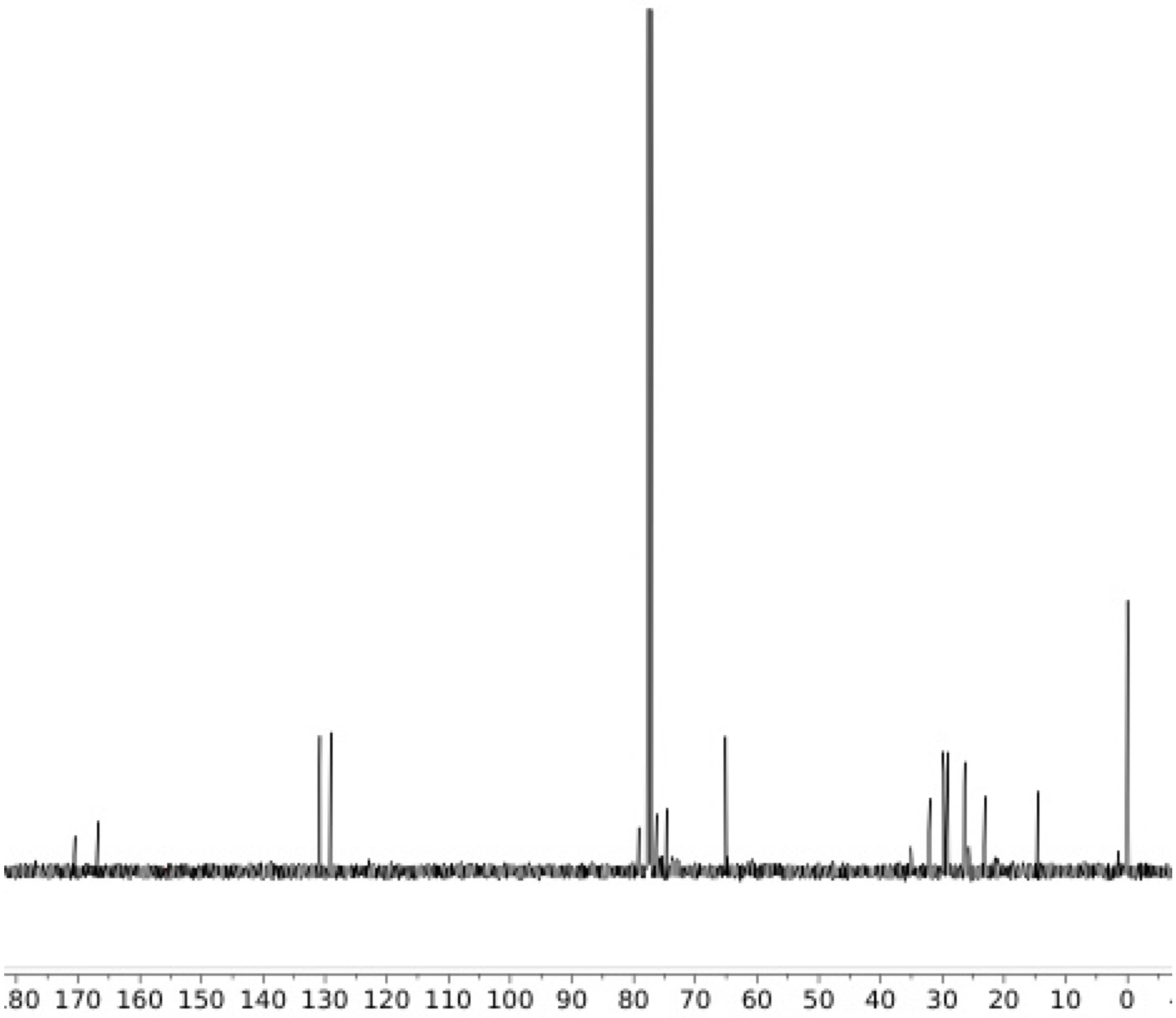


**Figure S44.** m-TMS-Fc diacrylate **(5)** CNMR.

**ESI-MS Spectra.**

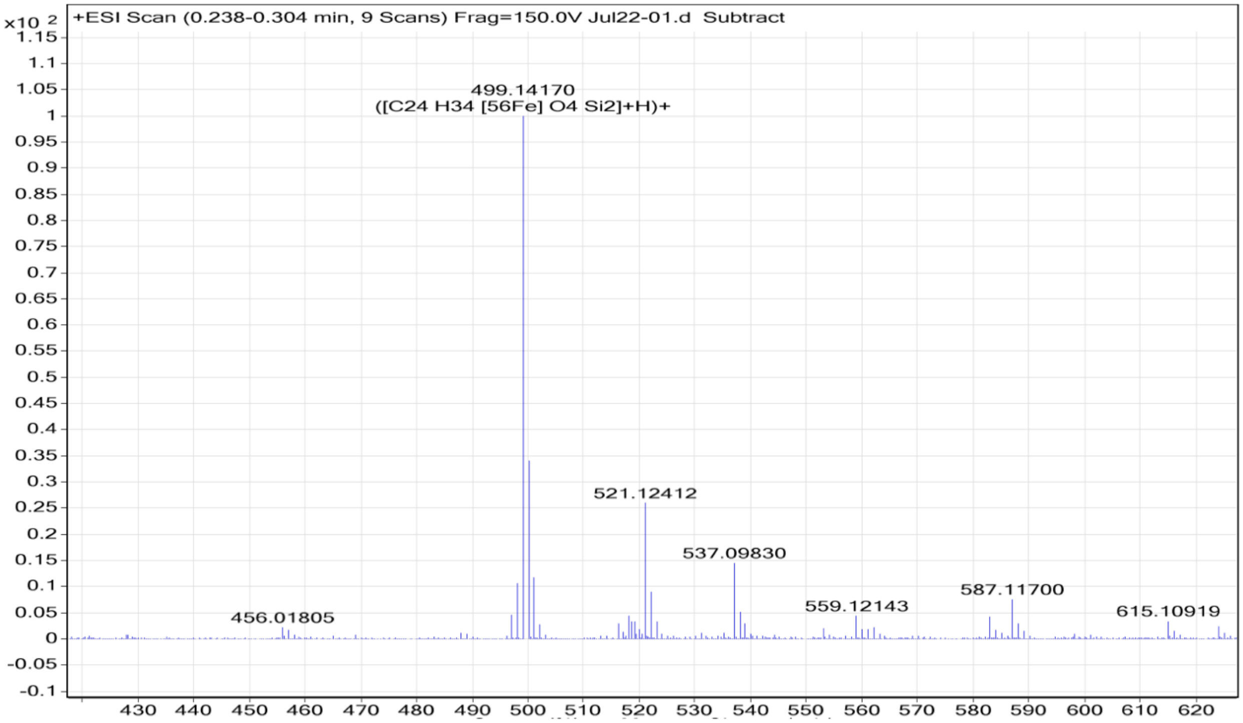


**Figure S45.** ESI-MS of **(3).** Confirmed by H+, Na+ adducts.

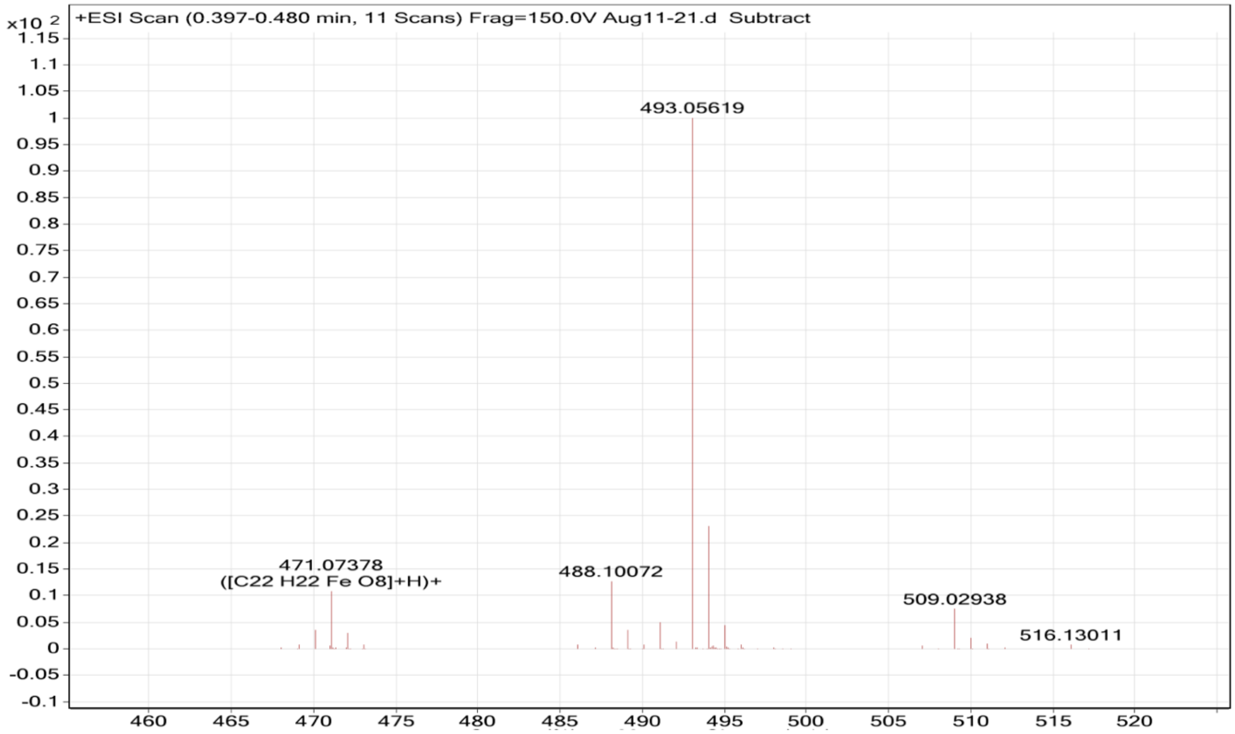


**Figure S46.** ESI-MS of **(4).** Confirmed by H+, Na+ adducts.

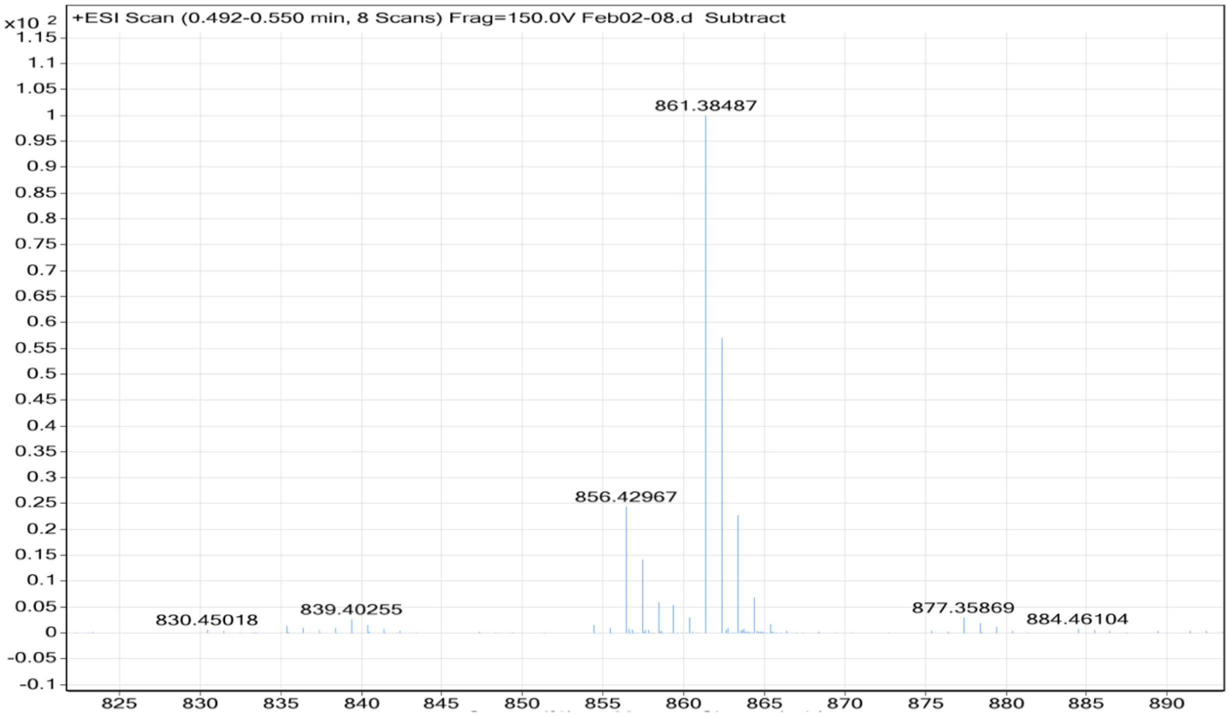


**Figure S47.** ESI-MS of **(5).** Confirmed by H+, Na+, K+ adducts.

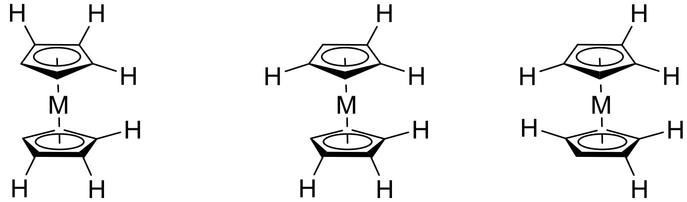


**Figure S48.** Structures considered in the first quarry. Carbon atoms without explicit hydrogen could either be substituted or unsubstituted.

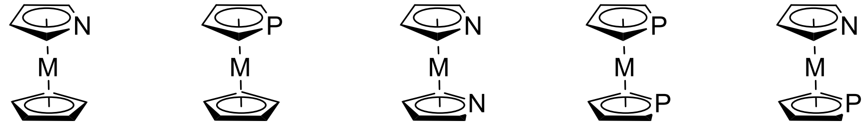


**Figure S49.** Structures considered in the second quarry.

**Text S12**. Description of Supplemental video.
Side-by-side tearing comparison of networks at identical stoichiometries containing, from left to right, a covalent linker (N1), a ferrocene control linker (N2), and m-TMS-ferrocene linker (N3). Video sped up ~80x.

**References**


1. Zenodo repository for High-Throughput Discovery of Ferrocene Mechanophores with Enhanced Reactivity and Network Toughening https://doi.org/10.5281/zenodo.13682716. (Accessed September 04, 2024).
2. Stevenson, R.; De Bo, G., Controlling Reactivity by Geometry in Retro-Diels–Alder Reactions under Tension. *Journal of the American Chemical Society* **2017,** *139* (46), 16768-16771.
3. Robb, M. J.; Kim, T. A.; Halmes, A. J.; White, S. R.; Sottos, N. R.; Moore, J. S., Regioisomer-Specific Mechanochromism of Naphthopyran in Polymeric Materials. *Journal of the American Chemical Society* **2016,** *138* (38), 12328-12331.
4. Küng, R.; Germann, A.; Krüsmann, M.; Niggemann, L. P.; Meisner, J.; Karg, M.; Göstl, R.; Schmidt, B. M., Mechanoresponsive Metal-Organic Cage-Crosslinked Polymer Hydrogels. *Chemistry – A European Journal* **2023,** *29* (18), e202300079.
5. Klein, I. M.; Husic, C. C.; Kovács, D. P.; Choquette, N. J.; Robb, M. J., Validation of the CoGEF Method as a Predictive Tool for Polymer Mechanochemistry. *Journal of the American Chemical Society* **2020,** *142* (38), 16364-16381.
6. Contreras-García, J.; Johnson, E. R.; Keinan, S.; Chaudret, R.; Piquemal, J.-P.; Beratan, D. N.; Yang, W., NCIPLOT: A Program for Plotting Noncovalent Interaction Regions. *Journal of Chemical Theory and Computation* **2011,** *7* (3), 625-632.
7. Bader, R. F. W., Atoms in molecules. *Accounts of Chemical Research* **1985,** *18* (1), 9-15.

8. Verloop, A.; Hoogenstraaten, W.; Tipker, J., Chapter 4 - Development and Application of New Steric Substituent Parameters in Drug Design. In *Drug Design*, Ariëns, E. J., Ed. Academic Press: Amsterdam, 1976; Vol. 11, pp 165-207.
9. Verloop, A., THE STERIMOL APPROACH: FURTHER DEVELOPMENT OF THE METHOD AND NEW APPLICATIONS. In *Pesticide Chemistry: Human Welfare and Environment*, Doyle, P.; Fujita, T., Eds. Pergamon: 1983; pp 339-344.
10. Huang, W.; Han, C. D., Synthesis and Intramolecular Charge-Transfer Interactions of a Donor–Acceptor Type Polymer Containing Ferrocene and TCNAQ Moieties. *Macromolecules* **2012,** *45* (10), 4425-4428.
11. Moore, J. S.; Stupp, S. I., Room temperature polyesterification. *Macromolecules* **1990,** *23* (1), 65-70.
12. Sha, Y.; Zhang, Y.; Xu, E.; Wang, Z.; Zhu, T.; Craig, S. L.; Tang, C., Quantitative and Mechanistic Mechanochemistry in Ferrocene Dissociation. *ACS Macro Letters* **2018,** *7* (10), 1174-1179.
13. Basavaiah, D.; Sarma, P. K. S., Terminal Hydroxyalkyl Acrylates as Substrates for Baylis-Hillman Reaction. *Synthetic Communications* **1990,** *20* (11), 1611-1615.
14. Wang, S.; Hu, Y.; Kouznetsova, T. B.; Sapir, L.; Chen, D.; Herzog-Arbeitman, A.; Johnson, J. A.; Rubinstein, M.; Craig, S. L., Facile mechanochemical cycloreversion of polymer cross-linkers enhances tear resistance. *Science* **2023,** *380* (6651), 1248-1252.
