# Supplementary material for: High-Throughput Discovery of Ferrocene Mechanophores with Enhanced Reactivity and Network Toughening
Source: ACS Cent Sci. 2025 Aug 1;11(10):1839–51. doi: 10.1021/acscentsci.5c00707 (PMC12550622; doi:10.1021/acscentsci.5c00707)
Supplement: Supplementary file 2 [file oc5c00707_si_002.pdf]

oc-2025-00707b.R1

Name: Peer Review Information for "High-Throughput Discovery of Ferrocene Mechanophores with Enhanced Reactivity and Network Toughening"

First Round of Reviewer Comments

Reviewer: 1

Comments to the Author

The authors developed and used HTVS approach to discover new mechanophores with improved mechanical lability. They incorporated adequate computations and ML models to derive mechanistic insights and identify of novel TM-mechanophores. The mechanistic insights are extremely valuable (steric crowding of dissociating ligands, rotational locking of Cp rings withligand–ligand noncovalent interactions, or through the introduction of functional groups that lead to metal–ligand interactions in the TS). It's especially exciting that the collaborative team also synthesized promising candidates from the computational search and screening. The manuscript should be published as is. Congratulations!

Reviewer: 2

Comments to the Author

The manuscript studies mechanophores with a focus on ferrocenes as elements in copolymers, due to their thermal stability and mechanical lability. The authors employ high-throughput virtual screening to identify ferrocene derivatives that are mechanically less stable than the unsubstituted parent compound. To this end, they train machine learning models — a combination of fully connected feed-forward neural networks and random forests — on quantum chemically calculated maximum resistance forces, thereby establishing a structure–force relationship.

After screening approximately 12,000 compounds, the authors select 8 ferrocene derivatives for further investigation using higher-level quantum chemical methods. However, the criteria used to select these specific 8 compounds are not explained such that any downstream success borders on luck and not as a clear result of efficient screening. Among these, a ferrocene derivative bearing a trimethylsilane substitution on the cyclopentadienyl rings is found to reduce mechanical resistance when the substituent is in the meta position relative to the polymer attachment points (in contrast to the ortho-substitution). This theoretical prediction is validated experimentally.

While the study addresses an interesting topic, the manuscript is challenging to follow. The methods and results are presented in entirely separate sections, requiring the reader to frequently jump between them — as well as to the Supporting Information — in order to understand the rationale behind key steps. The manuscript would benefit significantly from including brief (1–2 sentence) explanations within the Results section to clarify the purpose and function of specific methods as they are applied. Additionally, the text presumes a high level of familiarity with ferrocene chemistry, which may limit accessibility for the broad readership of ACS Central Science.

Given these issues, I cannot recommend the manuscript for publication in its current form.

Below, I list specific points that should be addressed before the paper can be reconsidered. (Note: line numbers refer to those printed in the margin of the manuscript and may not correspond to the actual number of lines from the top of the page.)

Introduction: Could the authors elaborate on the underlying mechanism by which incorporating a mechanically labile unit into a polymer can enhance the overall tear resistance of the material? This point is central to the motivation of the study and would benefit from clearer exposition. Please also consider explicitly stating this as the main objective of the work.

Page 3, line 39: The abbreviation "Cp" (cyclopentadienyl) is used without prior definition. Please introduce it at first mention for clarity.

Figure 1

* Consider defining the acronym "CSD" to ensure accessibility to readers unfamiliar with it.

* The combination of the virtual screening workflow and the scission mechanism examples in a single figure feels somewhat arbitrary. These might be more effectively communicated if split into two separate figures.

* The term "ground state" is used to describe the reactant minimum energy geometry, which could be misleading. "Ground state" typically refers to the electronic ground state, and its use here may cause confusion.

* Given the complexity of the scission mechanism and the central role it plays in the study, it would be helpful to include a schematic (e.g., as a new subfigure) that clearly illustrates what structural features are being analyzed. Specifically, indicate the polymer attachment points and how the relevant dihedral angle is defined.

Page 6, line 8: Please clarify whether "structure" refers to chemical structure rather than molecular geometry. As the study appears to focus on a single maximum force value (F_max) per compound, this distinction would aid understanding.

Page 6, line 38: Is there a rationale for using 3.5 nN as the cutoff value? If this choice is based on prior literature, empirical distribution, or domain knowledge, a brief explanation would strengthen the justification.

Figure 2

* In panel 2a, what does the distribution of green points represent?

* The orientation of the "strands" in the plot is unclear — many are tilted upward rather than parallel to the x-axis. Is there a reason for this pattern?

* Finally, the white marker indicating the median is difficult to see. Consider adjusting the color or contrast to improve visibility.

Page 7, line 50: Could the authors briefly explain what revised autocorrelations (RACs) are? As there are an increasing number of approaches for representing molecular structures as numerical vectors, it would be helpful to provide 1–2 sentences of context here. This could

also be a good place to direct the reader to Text S8 in the Supporting Information or to the computational details in Section 4.

Page 7, line 55: This would be a good point to include a reference to Table S5 in the Supporting Information, as well as to the relevant subsection in Section 4.

Page 8, line 18: Could the authors clarify whether the classifier incorrectly predicted some reactive samples as non-reactive, or if the majority of predictions were correctly classified as non-reactive, consistent with the distribution observed in the 425 DFT-computed compounds?

Page 8, line 54: It is not entirely clear which atoms define the dihedral angle in question, or what the polymer attachment points are. A clarification in the text or a simple schematic would help the reader understand how this geometric feature is defined.

Page 9, line 15: The transition to bridged ferrocene complexes occurs somewhat abruptly. The remainder of the study appears to focus primarily on unbridged ferrocenes (e.g., Figure 2a refers to “ML-unbridged”). It would help to introduce the discussion of bridged compounds more gradually or clarify how they relate to the main analysis.

Figure 3 and general comment on scatter/parity plots

* For plots comparing predicted vs. actual values, have the authors considered using hexbin plots with color-coded density? This could improve readability compared to scatter plots where points are irregularly distributed and yet color-coded by density.

* Specifically regarding Figure 3, the inclusion of training set performance may not be particularly informative, as it does not reflect generalization ability. Since the model was trained on this data, the training set results are expected to show low error. Could the authors clarify why they chose to include this panel?

Page 11, line 3: How do the authors define “less sterically crowded”? From Figure 3b, there does not appear to be a clear correlation — a linear fit would likely be nearly flat. A more detailed explanation of this assessment would be helpful.

Page 11, line 8: Are all the iron centers in these ferrocene derivatives not formally Fe(II)? If so, what specific charge are the authors referring to here? If this refers to a computed partial charge, it would be helpful to state this explicitly — for example, “Loewdin partial charge” — and mention the computational method used. Including this information would clarify the nature of the observed correlation.

Page 11, line 20: Please define what Sterimol parameters are. A short explanation or a reference would be useful for readers unfamiliar with this concept.

Page 11, line 34: The initial use of an artificial neural network (ANN) on a relatively small dataset is followed by a switch to random forest models after expanding the dataset. Could the authors clarify the motivation for this change in modeling approach beyond feature interpretability? Additionally, is there a reason why a SHAP analysis could not have been performed on the ANN, or why random forest models were not used from the outset?

Page 12, line 20: This paragraph highlights the need for a schematic figure — possibly added to Figure 1 — that clearly illustrates the design of the investigated complexes. Such a figure could define the attachment points, the relevant dihedral angle, and the positions where substituents or covalent bridges can be introduced.

Page 12, line 30: Have the authors considered evaluating how well the regression model would perform as a classifier by checking whether predicted values fall above or below the 3.5 nN threshold? Even a simple analysis of this sort could add valuable perspective on model reliability as the authors make a point that the important features for these two models are decidedly different.

Page 13, line 3: For readers unfamiliar with EFEI, it would be helpful to include a brief explanation — even a short clause such as "…(EFEI), which provides insight into XYZ…" would make the method more accessible.

Page 13, line 15: Consider writing out the full term behind the abbreviation "NCI" to remind the reader what it stands for.

Page 13, line 19: The bold six-letter code used to label compounds is not explained. What do these characters represent, and how are they assigned?

Page 13, line 28: Please define "TMS" at first use to ensure clarity for readers who may not be familiar with the abbreviation.

Page 13, line 31: Why are the eight selected compounds suddenly referred to as the "experimental set" in this subsection, which begins with the use of EFEI simulations? It would be helpful to clarify whether this set is purely computational, experimental, or a combination of both, and why this terminology is introduced at this point.

Page 13, line 36: The sentence "successfully capturing the relative barriers under applied force for the three species as measured by single-molecule force spectroscopy" is confusing. As far as I can tell, everything shown in Figure S25 is computational. How does this figure demonstrate agreement with experiment? Also, what is the significance of the horizontal black line in that plot?

Page 13, line 39: The referenced dihedral angles are not labeled in the figure. Readers cannot be expected to know which of the three ferrocene complexes have which dihedrals. A visual guide or explicit mention would improve clarity.

Page 14, line 22: How do the authors explain the low activation energy at small applied forces as shown in Figure S28? This behavior is somewhat counterintuitive and deserves further discussion.

Page 15, line 14: The sentence “Similarly, the extrapolated force-free m-TMS dissociation energy is…” could be misread as implying that “m-TMS” is a type of energy rather than the name of the complex. It would help to rephrase for clarity. Also, it is not immediately apparent that the mentioned y-intercept corresponds to the extrapolated zero-force activation energy. Lastly, please consider using a consistent term — either “activation energy” or “dissociation energy” — throughout this section.

Page 15, line 26 / Figure 5b: The statement about delocalization or stabilization due to TMS is difficult to verify from Figure 5b. The visualizations are cluttered, show the complexes from different perspectives, and red density appears near the C–Si bond in both cases. Consider simplifying the figure or providing a clearer visual or quantitative argument.

Page 16, line 52: How did the TMS-containing structure end up in the final subset of eight compounds studied with EFEI? Was this purely by chance? There are likely many compounds within the same Fmax range of 2.3 to 3.7 nN. How many such candidates are there in total? Some explanation would help rule out selection bias.

Page 17, line 32: How does the application of ultrasound relate to mechanical force? In this context, is the force truly applied via mechanical stretching of polymer strands? A short explanation of what is being done experimentally and why would help the reader follow the narrative without needing to cross-reference other sections.

Page 17, line 34 / Figure S29: What exactly are the ring-opening values Phi? Is this a probability, a fractional value, or something else? Does it have a unit? Also, consider including a version of Figure S29b in the main manuscript, as it would help readers understand the structure of the studied polymers and what aspect of their behavior is being optimized.

Page 17, line 36: Can gDDC also be cleaved under sufficient force? If so, does this make it scissile as well? It would be helpful if the authors could define what exactly they mean by “scissile” and add a brief explanation of what is measured or observed to support their conclusion.

Page 18, line 5: What precisely is meant by “tearing energy”? Is this an activation energy, a reaction energy, or something else? A short clarification would help avoid ambiguity.

Page 21, line 13: While the connection between material durability and sustainability is clear, it is less obvious how the incorporation of transition metal complexes contributes to renewability or recyclability. Could the authors elaborate on how these properties are enhanced through their use?

Page 23, line 18: What is the motivation for switching between functional, basis set, and quantum chemistry software? While a higher level of theory is often appropriate for a more detailed analysis, it is not clear why r2SCAN (a meta-GGA) is used here, as it is typically considered a lower rung than wPBEh (a range-separated hybrid). This choice deserves some justification.

Page 23, line 29: Why is a third functional with a smaller basis set used to validate the second functional? It seems more consistent to vary only one parameter at a time (e.g., increasing the basis set while keeping the functional fixed). Please clarify the rationale behind this approach.

Page 23, line 34: Why is water used as the solvent for CoGEF calculations, while THF is used for EFEI calculations, if both aim to describe the same mechanical tearing process? It would be helpful to explain whether this difference is due to methodological constraints, relevance to experiment, or another reason.

Page 23, section 4c: The explanation of RACs in the methods section is much more accessible than the version in the Supporting Information. Perhaps the SI could be revised to incorporate some of the clearer wording from the main text to improve readability.

Reviewer: 3

Comments to the Author

The manuscript reports the application of a variety of methods, with a focus on machine learning, to the study of the mechanical properties of polymers based on metallocene units. The work is very systematic, and goes to deep details in the explanation of machine-learning treatments, which is often unfortunately missing on many publications in the field. This work will constitute a useful addition to ACS Central Science after minor revision.

Even after admitting that the information supplied is very systematic, there are still some mostly minor details that can be improved.

The full specification of the CoGEF calculations requires information on the identify of the atoms is the mechanichal stress being placed. Are they in the ferrocene rings or on the substituents? I have not been able to find this information in the text.

In the Supporting Information, it is mentioned that two different searches were carried out on the CSD database. They are described, but reproducibility would be easier if the strings used in the search were specified.

The evaluation of the experimentally synthesized polymers assumes that single-strand sonication and polymer network tearing will have the same effect. As the techniques are far from identical, this requires some further comment.

The DFT calculations are said to have used a closed-shell formalism. This means that only heterolytic cleavage was considered. Some mention to the fact that the alternative of homolytic cleavage should be made.

Reviewer: 4

Comments to the Author

In this manuscript titled “High-Throughput Discovery of Ferrocene Mechanophores with Enhanced Reactivity and Network Toughening” which was contributed by Heather and co-workers, machine-learning strategy and EFEI calculations were conducted to explore the mechano-reactive ferrocene derivatives. Moreover, the computationally predicted species was then successfully employed in the practical preparation of bulk materials possessing a large tearing energy.

The work presented here is well-designed, and the process of finding a mechano-reactive ferrocene in the full chemical space is very logical. The authors first use a cheap CoGEF method to investigate the Fmax values of 425 complexes of ferrocene derivatives which are either bridged or unbridged. Subsequently they focused on those having an unbridged structure (between two Cp rings) and carried out a machine-learning study to elucidate the structure-mechanoreactivity relationship. Employing the ML algorithm here allows the authors to explore the complete database consisting of thousands of structures. It is definitely a big progress in the study of ferrocene mechanophores if compared to the previous trial-and-error studies. The EFEI is then used to locate the force-coupled transition state for a series of ferrocenes which were predicted to be mechanoreactive. Finally, a ferrocene labelled as m-TMS-Fc was chosen for the preparation of polymeric materials and it does show a good tear resistance property compared with the polymers made with another two cross-linker molecules.

In this work, both the computational study and the experimental verifications were carefully conducted, and the findings are very useful for the further development of ferrocene mechanophores. Therefore, I would like to recommend the publication in ACS central science if the following aspects can be well addressed.

1. It is well-known that iron complexes undergo spin-crossover under external stretching forces (Nano Lett. 2016, 16, 4733–4737; Nanoscale Adv. 2020, 2, 2907-2913). Also, the spin of Fe(II) complex highly depends on the ligands. I wonder, during the dissociation process of Cp ligand, whether the spin of Fe(II) has been changed. As far as I know, this issue was not addressed in the current version and all the complexes were treated as a closed-shell species. Thus, I would like to see at least for m-TMS-Fc, how the spin state of Fe(II) changes during the stretching.

2. The ball-and-stick models shown in all of the figures are difficult to recognize. A chemical formula should be also provided for a better understanding of the structures.

3. On page 2, the authors mentioned a predictive model for the mechanochemical scission (Ref. (30)). More citations are needed here since the only cited paper was not the first one to address how the structure affects the mechanoreactivity and its finding was based on many other works.

4. Citing papers on the discovery and design of self-strengthening hydrogel materials such as [https://doi.org/10.1021/jacs.1c12539, https://doi.org/10.1038/s41563-025-02137-6, https://doi.org/10.26434/chemrxiv-2022-fr09l] is recommended, as their design principles and theoretical frameworks are very similar to those of self-healing materials.

5. In Figure 4 and 5, the barrier-force relationships are fitted with linear equations. I am OK with it, but a large error is expected at low and high-force regions simply because it is actually not linear. Given this, the extension curves should be cut off and removed. The curve should be intercepted between 1.0 and 2.0 nN. In addition, I would like to see the error between the predicted activation force level and the experimental reported ones.

6. On page 33, the information of reference (74) and (75) is missing (e.g., journal name, volume, etc.).

7. The DFT calculations employed wPBEh and r2SCAN-3c functionals. As mentioned above, the system can easily undergo a spin crossover. The amount of Hartree-Fock

exchange adopted by the DFT functional significantly impacts the force-to-barrier response and coordination bond strength. Therefore, I strongly recommend carefully assessing these functionals and justifying the use of them instead of the more commonly used functionals such as M06, B3LYP, and PBE0.

8. It is interesting to see that introducing the reactive mechanophores into the polymeric material can in turn increase the toughness of it (resulting in a large tearing energy). I noticed that on page 18 the authors cited two papers (Ref (9) and (49)) to support this statement. I would like to see, at least, a brief discussion in the manuscript for the reason.

Author's Response to Peer Review Comments:

Heather J. Kulik **Massachusetts Institute of Technology**
Associate Professor of Chemical Engineering and Chemistry 77 Massachusetts Avenue, Building 66-464
Department of Chemical Engineering Cambridge, Massachusetts 02139

Department of Chemistry

PhoneEmail (617) 253hjkulik@mit.edu-
4584

June 10, 2025

Dear Editor,

Please find enclosed the resubmission of our paper entitled "High-Throughput Discovery of Ferrocene Mechanophores with Enhanced Reactivity and Network Toughening" for consideration of publication in *ACS Central Science*. The corresponding author is myself – contact information is given in the letterhead. All authors have seen and approved the submission of this manuscript, and it is being submitted exclusively to the *ACS Central Science*.

We have made the following changes as requested by the editor:
1) Abstract: we have shortened the abstract to 193 words.
2) References: We have updated reference 74.
3) Supporting Information Paragraph: We have shortened the description 4) Synopsis: We have included Synopsis following the TOC graphic

We have made changes according to concerns raised by the reviewers, as indicated below. Most of the reviewers were positive, but changes were requested to improve the clarity of the work. Reviewer comments are provided in full on pages 2-12 of this letter with response to each comment labeled "author

reply” and reproduction of corresponding changes to the text with associated page numbers. We have also provided a copy of the original manuscript with all changes marked in red.

Sincerely,

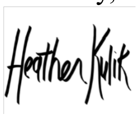

Heather J. Kulik

Reviewer #1:

The authors developed and used HTVS approach to discover new mechanophores with improved mechanical lability. They incorporated adequate computations and ML models to derive mechanistic insights and identify of novel TM-mechanophores. The mechanistic insights are extremely valuable (steric crowding of dissociating ligands, rotational locking of Cp rings with ligand-ligand noncovalent interactions, or through the introduction of functional groups that lead to metal- ligand interactions in the TS). It's especially exciting that the collaborative team also synthesized promising candidates from the computational search and screening. The manuscript should be published as is.

Response: We thank the reviewer for their time and careful consideration of our work.
Changes to text: None, see other revisions.

Reviewer #2:

The manuscript studies mechanophores with a focus on ferrocenes as elements in copolymers, due to their thermal stability and mechanical lability. The authors employ high-throughput virtual screening to identify ferrocene derivatives that are mechanically less stable than the unsubstituted parent compound. To this end, they train machine learning models — a combination of fully connected feed-forward neural networks and random forests — on quantum chemically calculated maximum resistance forces, thereby establishing a structure-force relationship.

After screening approximately 12,000 compounds, the authors select 8 ferrocene derivatives for further investigation using higher-level quantum chemical methods. However, the criteria used to select these specific 8 compounds are not explained such that any downstream success borders on luck and not as a clear result of efficient screening. Among these, a ferrocene derivative bearing a trimethylsilane substitution on the cyclopentadienyl rings is found to reduce mechanical resistance when the substituent is in the meta position relative to the polymer attachment points (in contrast to the ortho-substitution). This theoretical prediction is validated experimentally.

While the study addresses an interesting topic, the manuscript is challenging to follow. The methods and results are presented in entirely separate sections, requiring the reader to frequently jump between them — as well as to the Supporting Information — in order to understand the rationale behind key steps. The manuscript would benefit significantly from including brief (1–2 sentence) explanations within the Results section to clarify the purpose and function of specific methods as they are applied. Additionally, the text presumes a high level of familiarity with ferrocene chemistry, which may limit accessibility for the broad readership of ACS Central Science.

Given these issues, I cannot recommend the manuscript for publication in its current form.

Below, I list specific points that should be addressed before the paper can be reconsidered. (Note: line numbers refer to those printed in the margin of the manuscript and may not correspond to the actual number of lines from the top of the page.)

Response: We thank the reviewer for their time and consideration of our work.

Changes to text: None, see other revisions.

Introduction: Could the authors elaborate on the underlying mechanism by which incorporating a mechanically labile unit into a polymer can enhance the overall tear resistance of the material? This point is central to the motivation of the study and would benefit from clearer exposition. Please also consider explicitly stating this as the main objective of the work.

Response: We thank the reviewer for the helpful suggestion. We have added the mechanism for the tear resistance improvement with a relevant citation.

Changes to text: Added to page 2: "A combination of simulations and structure-activity relationships supported a mechanism for toughening in which each preferential scission of a side-chain crosslinker results in an effective increase the length of the highly tensioned strand, and the longer strand accommodates more stored elastic energy when it ultimately breaks to allow the crack to propagate[12]. As a result, the network can withstand greater stresses without tearing than it would if the high tension were constrained to only the strand located between the initial cross-linking points."

Page 3, line 39: The abbreviation "Cp" (cyclopentadienyl) is used without prior definition. Please introduce it at first mention for clarity.

Response: We have added the definition of Cp acronym at the first mention of the ligand.

Changes to text: Added on page 3 in blue: "This includes a shearing mechanism where unconstrained cyclopentadienyl (Cp) rings".

Figure 1 * Consider defining the acronym "CSD" to ensure accessibility to readers unfamiliar with it. * The combination of the virtual screening workflow and the scission mechanism examples in a single figure feels somewhat arbitrary. These might be more effectively communicated if split into two separate figures.
* The term "ground state" is used to describe the reactant minimum energy geometry, which could be misleading. "Ground state" typically refers to the electronic ground state, and its use here may cause confusion.
* Given the complexity of the scission mechanism and the central role it plays in the study, it would be helpful to include a schematic (e.g., as a new subfigure) that clearly illustrates what structural features are being analyzed. Specifically, indicate the polymer attachment points and how the relevant dihedral angle is defined.

Response: We thank the reviewer for the helpful suggestion. We have now split Figure 1 into two components. In Figure 1 (HTVS) we changed CSD in the description to Cambridge Structural Database. In figure 2, we have now included a schematic that demonstrates the mechanism of the two different transition states, and within the schematic, we have highlighted four atoms that define the structurally relevant dihedral angle. **Changes to text:** Figure 1 split to Figure 1 & Figure 2, with some changes within.

Page 7, line 50: Could the authors briefly explain what revised autocorrelations (RACs) are? As there are an increasing number of approaches for representing molecular structures as numerical vectors, it would be helpful to provide 1-2 sentences of context here. This could also be a good place to direct the reader to Text S8 in the Supporting Information or to the computational details in Section 4.

Response: We have included an expanded description of RACs feature set in the main text and referenced methods section and SI.
Changes to text: Added on page 8 in blue: "The RACs features are sums of products and differences of five atomwise properties (topology, identity, electronegativity, covalent radius and nuclear charge) on the molecular graph that have shown good performance for transition metal complex property predictions (see Computational Details and Supporting Information Text S8)."

Page 7, line 55: This would be a good point to include a reference to Table S5 in the Supporting Information, as well as to the relevant subsection in Section 4.

Response: We have added the reference to Sec. 4c and Table S5.
Changes to text: Changed on page 8 in blue: "We trained an artificial neural network (ANN) classifier in order to accelerate the screening of our entire dataset to identify additional ferrocenes with high reactivity (Supporting Information Table S5 and Sec. 4c)"

Page 8, line 18: Could the authors clarify whether the classifier incorrectly predicted some reactive samples as non-reactive, or if the majority of predictions were correctly classified as non-reactive, consistent with the distribution observed in the 425 DFT-computed compounds?

Response: We thank the reviewer for the comment. We have added the recall of the classifier to the main text. **Changes to text:** Added on page 8: "Our ANN classification model demonstrates promising performance on a setaside 20% test set, identifying the majority (85%) of more reactive complexes accurately (Figure 2b and Supporting Information Figure S2 and Table S1)."

Page 9, line 15: The transition to bridged ferrocene complexes occurs somewhat abruptly. The remainder of the study appears to focus primarily on unbridged ferrocenes (e.g., Figure 2a refers to "ML-unbridged"). It would help to introduce the discussion of bridged compounds more gradually or clarify how they relate to the main analysis.

Response: We thank the reviewer for the comment. Bridged, a.k.a. ansa-ferrocenes were used as reference because they have experimentally demonstrated increased reactivity with an established feature related to the dihedral angle between attachment points. We have added the reference to this to explain why we used this dihedral angle in our analysis.

Changes to text: Added on page 9 "Among the experimentally studied ferrocene mechanophores, bridged- or ansaferrocenes have shown the highest reactivity, and lower dihedral angle between attachment points was shown to lower activation energies for ansa-ferrocenes."

Figure 3 and general comment on scatter/parity plots

* For plots comparing predicted vs. actual values, have the authors considered using hexbin plots with color-coded density? This could improve readability compared to scatter plots where points are irregularly distributed and yet color-coded by density. * Specifically regarding Figure 3, the inclusion of training set performance may not be particularly informative, as it does not reflect generalization ability. Since the model was trained on this data, the training set results are expected to show low error. Could the authors clarify why they chose to include this panel?

Response: We have updated the parity plot to be a hexbin plot and have removed the training set from the parity plot

Changes to text: Updated Figure 3 (now Figure 4)

Page 12, line 3: How do the authors define "less sterically crowded"? From Figure 3b, there does not appear to be a clear correlation — a linear fit would likely be nearly flat. A more detailed explanation of this assessment would be helpful.

Response: We have extended the description on how SASA is related to steric crowding. Furthermore, while we don't see SASA alone having strong correlation with forces, it is notable that the rate at which more reactive complexes appear is increased with decrease of SASA.

Changes to text: added: "Given that lower SASA means Cp ring has lower solvent-accessible surface area, when the maximum SASA among two ligands is low, it implies that both ligands are sterically congested. While this descriptor doesn't have a high correlation, we observe increased rate of low activation forces when this descriptor is small."

Page 11, line 8: Are all the iron centers in these ferrocene derivatives not formally Fe(II)? If so, what specific charge are the authors referring to here? If this refers to a computed partial charge, it would be helpful to state this explicitly — for example, "Loewdin partial charge" — and mention the computational method used.

Including this information would clarify the nature of the observed correlation.

Response: We thank the reviewer for the helpful suggestion. We have made the suggested changes.

Changes to text: Changed text on page 12 in blue: "Furthermore, we observe that the Mulliken partial charge on the iron center in the ground state calculated from the DFT optimized geometry is also moderately positively correlated with the F_{max} values"

Page 11, line 20: Please define what Sterimol parameters are. A short explanation or a reference would be useful for readers unfamiliar with this concept.

Response: We thank the reviewer for the suggestion. We have added reference 54 to the text.

Changes to text: Added reference to the text on page 12: "Other electronic and steric descriptors that relate to both metal-local, and more global steric and electronic trends, such as Sterimol parameters"

Page 11, line 34: The initial use of an artificial neural network (ANN) on a relatively small dataset is followed by a switch to random forest models after expanding the dataset. Could the authors clarify the motivation for this change in modeling approach beyond feature interpretability? Additionally, is there a reason why a SHAP analysis could not have been performed on the ANN, or why random forest models were not used from the outset?

Response: We thank the reviewer for the comment. We initially selected ANNs as they showed better performance in terms of identifying more reactive complexes (recall) when compared to RF. We have now included initial RF model performance metrics in the SI and referenced it in the main text. The main reason for training the final model was to analyze features, and while we agree with referee's comments that SHAP analysis could have been used here, RF models gave sufficient agreement with the experiments and offer a more interpretable framework, as discussed in the text.

Changes to text: Added to page 8 in blue: "We chose an ANN because it offered a better performance for classification, and in particular, performed much better at identifying reactive complexes on the set aside test set (Supporting Information Table S1)."

Page 12, line 20: This paragraph highlights the need for a schematic figure — possibly added to Figure 1 — that clearly illustrates the design of the investigated complexes. Such a figure could define the attachment points, the relevant dihedral angle, and the positions where substituents or covalent bridges can be introduced.

Response: We thank the reviewer for the suggestion. Figure 2 now includes a schematic.

Changes to text: Added schematic to the new Figure 2.

Page 12, line 30: Have the authors considered evaluating how well the regression model would perform as a classifier by checking whether predicted values fall above or below the 3.5 nN threshold? Even a simple analysis of this sort could add valuable perspective on model reliability as the authors make a point that the important features for these two models are decidedly different.

Response: We thank the reviewer for the suggestion. We have now included in the text, the performance of the regression model as the classifier.

Changes to text: Added to page 13: "While models have distinct features, the regression model can still correctly classify 76% of the complexes if treated as a classification model."

Page 13, line 3: For readers unfamiliar with EFEI, it would be helpful to include a brief explanation — even a short clause such as "…(EFEI), which provides insight into XYZ…" would make the method more accessible.

Response: We thank the reviewer for the suggestion and have provided rational for the use of EFEI calculations. **Changes to text:** Added to page 14: "EFEI calculations can be used to build force-modified potential energy surfaces, and provide additional mechanistic insights into factors governing reactivity"

Page 13, line 15: Consider writing out the full term behind the abbreviation "NCI" to remind the reader what it stands for.

Response: Defined non-covalent interactions (NCI) at the first mention.
Changes to text: On page 14 changed to: "a non-covalent interaction (NCI) set"

Page 13, line 19: The bold six-letter code used to label compounds is not explained.
What do these characters represent, and how are they assigned?

Response: We thank the reviewer for pointing out the confusion with unique identifiers of CSD - refcodes. Now, before mentioning any CSD refcodes, we first label them with a prefix of "CSD refcode:"
Changes to text: On page 15 changed in blue: "CSD refcode: **EGIPER**", "CSD refcode: **EFILAI**", "CSD refcode:
BADXOU"

Page 13, line 28: Please define "TMS" at first use to ensure clarity for readers who may not be familiar with the abbreviation.

Response: We have defined trimethylsilyl at its first mention.
Changes to text: On page 14 change text in blue: to "bulky trimethylsilyl (TMS) group "

Page 13, line 31: Why are the eight selected compounds suddenly referred to as the "experimental set" in this subsection, which begins with the use of EFEI simulations? It would be helpful to clarify whether this set is purely computational, experimental, or a combination of both, and why this terminology is introduced at this point.

Response: Experimental set refers to three complexes previously studied experimentally for mechanochemical activation. We have now added i.e. experimental set in text when first mentioning three experimental complexes. **Changes to text:** added to page 14: "i.e. experimental set"

Page 13, line 36: The sentence "successfully capturing the relative barriers under applied force for the three species as measured by single-molecule force spectroscopy" is confusing. As far as I can tell, everything shown in Figure S25 is computational. How does this figure demonstrate agreement with experiment? Also, what is the significance of the horizontal black line in that plot?

Response: This refers to previous experimental work. We have modified this sentence by adding "in the previous work", with an appropriate citation. The Horizontal black line is defined in the caption referring to the energy needed for the reaction to occur under experimental SMFS experiments. **Changes to text:** Added to Page 15 in blue: "in the previous work"

Page 13, line 39: The referenced dihedral angles are not labeled in the figure. Readers cannot be expected to know which of the three ferrocene complexes have which dihedrals. A visual guide or explicit mention would improve clarity.

Response: We thank the reviewer for the suggestion. Now we define relevant chemical groups in text and include a schematic that highlights these groups.
Changes to text: Added to page 15 in blue: "the APs (i.e. ethyl groups) are nearly aligned in **EFILAI**". Added to Figure 5 (previously Figure 4), schematic representation of two complexes with ethyl groups highlighted.

Page 14, line 22: How do the authors explain the low activation energy at small applied forces as shown in Figure S28? This behavior is somewhat counterintuitive and deserves further discussion.

Response: We thank the reviewer for this comment. We now explicitly point out that the H-bonding gets disrupted at higher forces (i.e. 1.25 nN and above) to explain the observed behavior.

Changes to text: Added to page 16 in blue: "However, when ligand–ligand interaction is weak, as is the case for the complex **BADXOU**, hydrogen bonding is easily disrupted at forces above 1.0 nN external force, resulting in a lower activation energy at 1.00 nN external force. Consequently, the complex behaves like unsubstituted ferrocene at higher forces (Supporting Information Figure S28)."

Page 15, line 14: The sentence "Similarly, the extrapolated force-free m-TMS dissociation energy is…" could be misread as implying that "m-TMS" is a type of energy rather than the name of the complex. It would help to rephrase for clarity. Also, it is not immediately apparent that the mentioned y-intercept corresponds to the extrapolated zero-force activation energy. Lastly, please consider using a consistent term — either "activation energy" or "dissociation energy" — throughout this section. **Response:** We thank the reviewer for the helpful comment. We have rephrased the sentence and changed dissociation energy to activation energy, which is consistently used throughout the rest of the manuscript. Given that the linear fit corresponds to the activation energy as a function of force, the intercept would therefore be activation energy when force is equal to 0. This linear model is commonly used for extrapolating tension-free reactivity.

Changes to text: Changed on page 17 in blue: "While linearity of force-dependent activation energies can deviate at very low and very high forces, the intercept still provides valuable qualitative insights on relative thermal activation energies. Similarly, the extrapolated force-free activation energy of m-TMS ferrocene is also substantially (i.e., 8 kcal/mol) lower than that of unsubstituted ferrocene (Figure 6a). "

Page 15, line 26 / Figure 5b: The statement about delocalization or stabilization due to TMS is difficult to verify from Figure 5b. The visualizations are cluttered, show the complexes from different perspectives, and red density appears near the C-Si bond in both cases. Consider simplifying the figure or providing a clearer visual or quantitative argument.

Response: We thank the reviewer for the helpful comment. We have included the results of energy decomposition analysis (ALMO-EDA) in the SI that further corroborate the findings and show that TMS substituent stabilizes reactivity through favorable interactions in the TS, that are only present when the substituent is meta to the pulling direction.

Changes to text: Added to page 17 in blue: "These findings are further supported by energy decomposition analysis[50] (ALMO-EDA), which reveals that the most favorable changes in interaction energies, when compared to unsubstituted ferrocene, result from increased intramolecular polarization, leading to stronger electrostatic interactions and not from improved strain relief through either lower distortion or lower Pauli repulsion in the TS (Supporting Information Table S7)."; "and the force-modified reactivity of o-TMS-Fc strongly resembles that of unsubstituted ferrocene (Figure 6). This analysis is again consistent with the findings from ALMO-EDA (Supporting Information Table S7)."

Page 16, line 52: How did the TMS-containing structure end up in the final subset of eight compounds studied with EFEI? Was this purely by chance? There are likely many compounds within the same Fmax range of 2.3 to 3.7 nN. How many such candidates are there in total? Some explanation would help rule out selection bias.

Response: All of the personal selection was driven by synthetic considerations that are independent of the computational screening for reactivity.

Changes to text: Added to page 14: **"**Because of our intention to test the results of the computations in experiments, we also evaluated the synthetic accessibility as follows. First, our standard method for attaching crosslinking groups to ferrocene derivatives involves lithiation of the Cp rings, and so we prioritized compounds with functional groups that were likely to be compatible with that methodology. Second, we made an initial appraisal of the likely synthetic effort that would be required and prioritized compounds that we perceived to require less intensive synthesis. No additional attempt to judge the likely mechanochemical reactivity of the candidate complexes was made or considered."

`Page 17, line 32: How does the application of ultrasound relate to mechanical force? In this context, is the force truly applied via mechanical stretching of polymer strands? A short explanation of what is being done experimentally and why would help the reader follow the narrative without needing to cross-reference other sections.`

Response: We agree that this will make this section of the paper much easier to read and have provided the requested explanation.

Changes to text: Added to page 19. "The polymers were then characterized by pulsed ultrasonication of solutions of the polymers, which is the most commonly employed technique for characterizing mechanochemical reactivity in polymers. Sonication leads to cavitation of gas bubbles within the solution, and the bubble collapse creates an elongational flow field in the solution that rushes to center of the collapsing bubbles and stretches polymer chains caught within it. As the polymers stretch, force increases, and two mechanochemical events are possible. First, the *g*DCC mechanophores can open in a non-scissile fashion (i.e., without the polymer breaking) to give a 2,3dichloroalkene product that can be quantified by ^{1}H NMR. These ring opening reactions continue to occur along high force regions of the polymer until either the bubble completes its collapse or the second mechanochemical event occurs – the polymer chain breaks, which can be quantified by size exclusion chromatography and multiangle light scattering. Because neither the *g*DCC nor its ring opened product are themselves sites of preferred scission, the extent of *g*DCC reactivity per chain breaking event gives an indication of the relative mechanical strength of the weakest bonds along the rest of the polymer backbone."

`Page 17, line 34 / Figure S29: What exactly are the ring-opening values Phi? Is this a probability, a fractional value, or something else? Does it have a unit? Also, consider including a version of Figure S29b in the main manuscript, as it would help readers understand the structure of the studied polymers and what aspect of their behavior is being optimized.`

Response: Phi is a fractional value and it is unitless. We have added text to clarify. We have also moved Figure S29 to the main text as Figure 7.

Changes to text: Added to page 21: "The amount of *g*DCC ring opening is expressed as φ, the fraction of *g*DCC mechanophores that have reacted, and we evaluate φ relative to the number of scission events per chain in order to achieve a direct competition."

`Page 17, line 36: Can gDDC also be cleaved under sufficient force? If so, does this make it scissile as well? It would be helpful if the authors could define what exactly they mean by "scissile" and add a brief explanation of what is measured or observed to support their conclusion.`

Response: gDCC is not itself cleaved, nor does its product appear to any more susceptible to scission than a conventional polymer backbone. We have now stated that and clarified the term "scissile".

Changes to text: Added to page 20: "Because neither the gDCC nor its ring opened product are themselves sites of preferred scission, the extent of gDCC reactivity per chain breaking event gives an indication of the relative mechanical strength of the weakest bonds along the rest of the polymer backbone." and "First, the *g*DCC mechanophores can open in a non-scissile fashion (i.e., without the polymer breaking)"

Page 18, line 5: What precisely is meant by "tearing energy"? Is this an activation energy, a reaction energy, or something else? A short clarification would help avoid ambiguity.

Response: We thank the reviewer for bringing this to our attention and have defined the term in the main text.

Changes to text: Added to page 22: "By tearing energy, we refer to the energy stored in applied stress to the network that is necessary for a crack to propagate through the material. A larger tearing energy therefore corresponds to a material that is more difficult to tear."

Page 21, line 13: While the connection between material durability and sustainability is clear, it is less obvious how the incorporation of transition metal complexes contributes to renewability or recyclability. Could the authors elaborate on how these properties are enhanced through their use?

Response: We thank the reviewer for the comment. To clarify that our intention was regarding discovery of other mechanophores, we have updated the sentence.

Changes to text: Added on page 25 in blue: "The approach has the potential to further integrate diverse mechanophore discovery into advanced polymer material design, including those based on renewable, recyclable feedstocks."

Page 23, line 18: What is the motivation for switching between functional, basis set, and quantum chemistry software? While a higher level of theory is often appropriate for a more detailed analysis, it is not clear why r2SCAN (a meta-GGA) is used here, as it is typically considered a lower rung than wPBEh (a range-separated hybrid).
This choice deserves some justification.

Response: We thank the reviewer for the comment. EFEI calculations were carried out on a different software package, where the wPBEh functional was not available. Furthermore, r2scan-3c has demonstrated high accuracy at relatively lower cost and, given the number of transition state structures necessary to build force modified potential energy surfaces, we opted to choose a more affordable method. This is now stated explicitly in the methods section.

Changes to text: Added on page 27 in blue: "A low-cost, highly optimized method was chosen to accelerate the optimization of several transition state structures needed to build force dependence curves."

Page 23, line 29: Why is a third functional with a smaller basis set used to validate the second functional? It seems more consistent to vary only one parameter at a time (e.g., increasing the basis set while keeping the functional fixed). Please clarify the rationale behind this approach.

Response: We thank the reviewer for spotting the typo in the basis set. As stated in the SI, geometries were obtained using a smaller basis set, but the energies were evaluated using def2-tzvp basis set. We have now correctly stated the basis set used for benchmarking r2scan against a range-separated hybrid functional (def2-tzvp, not def2-svp). **Changes to text:** Changed on page 27 in blue: "we computed force modified ligand dissociation activation energies of unsubstituted ferrocene using ωB97x-D4/def2-TZVP, with geometries optimized at ωB97x-D4/def2-SVP level of theory"

Page 23, line 34: Why is water used as the solvent for CoGEF calculations, while THF is used for EFEI calculations, if both aim to describe the same mechanical tearing process? It would be helpful to explain whether this difference is due to methodological constraints, relevance to experiment, or another reason.

Response: As stated in text, we chose THF for additional calculations to match experimental conditions. We have now included further clarification as to which experiments we were referring to.

Changes to text: Added on page 27 in blue "with a dielectric of $\varepsilon = 7.25$ to model the experimental THF solvent in sonication experiments"

Page 23, section 4c: The explanation of RACs in the methods section is much more accessible than the version in the Supporting Information. Perhaps the SI could be revised to incorporate some of the clearer wording from the main text to improve

Response: We appreciate the reviewer's concern. We note that the two text sections serve different purposes - the purpose of Text S8 is to elaborate on fine technical details for anyone wishing to reproduce our work. We have clarified this point in the body of Text S8 (Now Text S2) and added a general clarifying statement about RACs. **Changes to text:** Added to SI Text S8 (Now Text S2): "RACs are graph-based representations that are products and differences in the molecular graph of heuristic properties. They therefore incorporate information about connectivity but do not directly encode through-space interactions. A short description of RACs is provided in the main text. Here, we provide more technical details regarding the specific implementation of RACs used in this work to enable reproducibility."

Reviewer #3:

The manuscript reports the application of a variety of methods, with a focus on machine learning, to the study of the mechanical properties of polymers based on metallocene units. The work is very systematic, and goes to deep details in the explanation of machine-learning treatments, which is often unfortunately missing on many publications in the field. This work will constitute a useful addition to ACS Central Science after minor revision.

Even after admitting that the information supplied is very systematic, there are still some mostly minor details that can be improved.

Response: We thank the reviewer for their time and careful consideration of our work.

Changes to text: None, see other revisions.

The full specification of the CoGEF calculations requires information on the identify of the atoms is the mechanichal stress being placed. Are they in the ferrocene rings or on the substituents? I have not been able to find this information in the text.

Response: We thank the reviewer for the comment. We have added more explicit mention on how the CoGEF was carried out and we have also included a schematic that explicitly shows where the force is applied in the new Figure 2.

Changes to text: Added to page 27 in blue: "The constrained geometries simulate external force (CoGEF) calculations were carried out by increasing terminal carbon atom distances at 0.2 Å increments using the TRIC optimizer, while all other internal coordinates were allowed to relax (Figure 2)."

In the Supporting Information, it is mentioned that two different searches were carried out on the CSD database. They are described, but reproducibility would be easier if the strings used in the search were specified.

Response: We thank the reviewer for the suggestion. We have included structures considered for each search in the SI.
Changes to text: Added to page S3 in the SI in blue: "Furthermore, each Cp ring was restricted to containing at most two substituents on the five-membered ring to ensure greater synthetic accessibility (Supporting Information Figure S48)."; "Due to the limited size of the second set, no constraint was placed on the number of substitutions applied to the Cp rings (Supporting Information Figure S49)"

The evaluation of the experimentally synthesized polymers assumes that singlestrand sonication and polymer network tearing will have the same effect. As the techniques are far from identical, this requires some further comment.

Response: We thank the reviewer for bringing the need for clarification on this point to our attention, and we
have revised the text accordingly.
Changes to text: On page 22, we have added: "The sonication experiments had confirmed experimentally in single chains what the computations predicted, namely that m-TMS-Fc will break at lower force than the other, more conventional components of the polymer. Based on our earlier studies[12], we therefore expected that same preferential mechanochemical reactivity to give rise to mechanophore toughening effects in material tearing tests, even though the sonication and tearing experiments are very different techniques."

The DFT calculations are said to have used a closed-shell formalism. This means that only heterolytic cleavage was considered. Some mention to the fact that the alternative of homolytic cleavage should be made.

Response: We thank the reviewer for the comment. Only heterolytic cleavage was considered in this work because past experimental and computational work suggests that this is operative mechanism for mechanochemical ferrocene dissociation. This is now explicitly stated in the methods section.
Changes to text: Added to page 27 in blue: "A closed shell singlet was chosen because past experimental and computational work has demonstrated that mechanochemical ferrocene cleavage proceeds through heterolytic cleavage[36], and closed shell treatment has the added benefit of being lower in computational cost."

<u>Reviewer #4:</u>

In this manuscript titled "High-Throughput Discovery of Ferrocene Mechanophores with Enhanced Reactivity and Network Toughening" which was contributed by Heather and co-workers, machine-learning strategy and EFEI calculations were conducted to explore the mechano-reactive ferrocene derivatives. Moreover, the computationally predicted species was then successfully employed in the practical preparation of bulk materials possessing a large tearing energy.

The work presented here is well-designed, and the process of finding a mechanoreactive ferrocene in the full chemical space is very logical. The authors first use a cheap CoGEF method to investigate the Fmax values of 425 complexes of ferrocene derivatives which are either bridged or unbridged. Subsequently they focused on those having an unbridged structure (between two Cp rings) and carried out a machine-learning study to elucidate the structure-mechanoreactivity relationship. Employing the ML algorithm here allows the authors to explore the complete database consisting of thousands of structures. It is definitely a big progress in the study of ferrocene mechanophores if compared to the previous trialand-error studies. The EFEI is

then used to locate the force-coupled transition state for a series of ferrocenes which were predicted to be mechanoreactive. Finally, a ferrocene labelled as m-TMS-Fc was chosen for the preparation of polymeric materials and it does show a good tear resistance property compared with the polymers made with another two cross-linker molecules.

In this work, both the computational study and the experimental verifications were carefully conducted, and the findings are very useful for the further development of ferrocene mechanophores. Therefore, I would like to recommend the publication in ACS central science if the following aspects can be well addressed.

Response: We thank the reviewer for their time and careful consideration of our work.
Changes to text: None, see other revisions.

1. It is well-known that iron complexes undergo spin-crossover under external stretching forces (Nano Lett. 2016, 16, 4733−4737; Nanoscale Adv. 2020, 2, 29072913). Also, the spin of Fe(II) complex highly depends on the ligands. I wonder, during the dissociation process of Cp ligand, whether the spin of Fe(II) has been changed. As far as I know, this issue was not addressed in the current version and all the complexes were treated as a closed-shell species. Thus, I would like to see at least for m-TMS-Fc, how the spin state of Fe(II) changes during the stretching.

Response: We have calculated the spin splitting energies of m-TMS-Fc under 1 nN and under 2nN external force, and they indicate that the low spin state is significantly more favorable than the high spin state. Several methods were evaluated and all indicate the same.
Changes to text: Added to page 25 in blue: "Calculations were carried out using the C-PCM implicit solvent model with a dielectric of $\varepsilon = 7.25$ to model the experimental THF solvent in sonication experiments. To ensure that no spin crossover occurs at highly tensioned states, we evaluated the vertical spin splitting energies for the mTMS-Fc complex at 1 and 2 nN external applied force (Supporting Information Table S6). These results indicate that, regardless of the level of theory, the singlet state is significantly more stable than the quintet state. "

2. The ball-and-stick models shown in all of the figures are difficult to recognize. A chemical formula should be also provided for a better understanding of the structures.

Response: We thank the reviewer for the comment. We have added 2D structures of the most important complexes in Figures 5 and 6 (Previously Figures 4 and 5). **Changes to text:** Updated Figures 5 and 6.

3. On page 2, the authors mentioned a predictive model for the mechanochemical scission (Ref. (30)). More citations are needed here since the only cited paper was not the first one to address how the structure affects the mechanoreactivity and its finding was based on many other works.

Response: We thank the reviewer for pointing this out. We have added references 34 and 35.
Changes to text: Added on page 3: references 34 and 35.

4. Citing papers on the discovery and design of self-strengthening hydrogel materials such as [https://doi.org/10.1021/jacs.1c12539, https://doi.org/10.1038/s41563-025-021376, https://doi.org/10.26434/chemrxiv-2022-fr09l] is recommended, as their design principles and theoretical frameworks are very similar to those of self-healing materials.

Response: We thank the reviewer. We have added the suggested references.
Changes to text: Added to page 2 in blue: "Mechanophores exhibit a wide range of chemical responses to force, including covalent rearrangement[2], the release of cargo[3], self-strengthening hydrogels[4-6], and changed catalytic activity[7]."

5. In Figure 4 and 5, the barrier-force relationships are fitted with linear equations. I am OK with it, but a large error is expected at low and high-force regions simply because it is actually not linear. Given this, the extension curves should be cut off and removed. The curve should be intercepted between 1.0 and 2.0 nN. In addition, I would like to see the error between the predicted activation force level and the experimental reported ones.

Response: We thank the reviewer for this suggestion. Unfortunately, experimental forces cannot be measured with scissile mechanophores, only relative trends can be inferred. We agree in principle that Bell's model only works at moderate forces, and for this reason, curves are derived from 1-2 nN force range. However, we believe that y-intercept is still informative in spite of mentioned deviation from linearity. We now mention this explicitly in text.
Changes to text: Added to page 17 in blue: "While linearity of force-dependent activation energies can deviate at very low and very high forces, the intercept still provides valuable qualitative insights on relative thermal activation energies."

6. On page 33, the information of reference (74) and (75) is missing (e.g., journal name, volume, etc.).

Response: We thank the reviewer for noticing the error. Citations 74 and 75 (now 81 and 82) have been fixed. **Changes to text:** On page 34, updated references 81,82.

7. The DFT calculations employed wPBEh and r2SCAN-3c functionals. As mentioned above, the system can easily undergo a spin crossover. The amount of Hartree-Fock exchange adopted by the DFT functional significantly impacts the force-to-barrier response and coordination bond strength. Therefore, I strongly recommend carefully assessing these functionals and justifying the use of them instead of the more commonly used functionals such as M06, B3LYP, PBE, and PBE0.

Response: We thank the reviewer for this comment. We have benchmarked vertical spin splitting energies using r2scan-3c, M06, B3LYP, PBE, PBE0, and wB97x-D3 and consistently see that singlet state is significantly more stable than the quintet state.
Changes to text: See **R4#1**.

8. It is interesting to see that introducing the reactive mechanophores into the polymeric material can in turn increase the toughness of it (resulting in a large tearing energy). I noticed that on page 18 the authors cited two papers (Ref (9) and (49)) to support this statement. I would like to see, at least, a brief discussion in the manuscript for the reason.

Response: Thank you for the great suggestion. We have added discussion on this point in two places in the manuscript.
Changes to text: Added to page 2: "A combination of simulations and structure-activity relationships supported a mechanism for toughening in which each preferential scission of a side-chain crosslinker results in an effective increase the length of the highly tensioned strand, and the longer strand accommodates more stored elastic energy when it ultimately breaks to allow the crack to propagate[12]. As a result, the network can withstand greater stresses without tearing than it would if the high tension were constrained to only the strand located between the initial cross-linking points."

And on page 21: "The mechanochemical reactivity is critical to this effect, because the mechanism requires that when a polymer strand between two crosslinks is under high tension, one of the crosslinkers will break prior to scission of the strand itself – even if the crosslinker is under less tension than the strand in question. The selective scission of the crosslinker effectively increases the length of the highly tensioned strand, when now extends to the next crosslinker in sequence along the primary chain within the network. The effective lengthening of the higly tensioned strand leads to more elastic energy that must be stored in the strand when it stretched up to the point that it eventually breaks or (if no more crosslinkers remain) slips out of the network to allow a growing crack to propagate[12]."

oc-2025-00707b.R2

Name: Peer Review Information for "High-Throughput Discovery of Ferrocene Mechanophores with Enhanced Reactivity and Network Toughening"

Second Round of Reviewer Comments

Reviewer: 2

Comments to the Author

The authors have taken my comments to heart and have made the manuscript much more accessible to the broad readership of ACS Central Science. In my opinion the paper can be published after the following minor points have been fixed:

- Please check all the inserted (highlighted) statements for English grammar. Examples are

"Given that lower SASA means Cp ring has lower solvent-accessible surface area, when the maximum SASA among two ligands is low,..."

"The selective scission of the crosslinker effectively increases the length of the highly tensioned strand, when now extends to the next crosslinker in sequence along the primary chain within the network."

- Figure 6: I thank the authors for the added ALMO-EDA analysis and added pictures to subfigure a. Consider first mentioning all text regarding subfigure an and then mentioning subfigure b. Right now the authors jump back and forth in the caption. Also, "a" and "b" should be bold face.

- Figure 7: The caption should define "ring opening (Phi)" to make the figure accessible without searching in the text. Also the text uses small phi whereas the figure uses capital Phi.

Reviewer: 4

Comments to the Author

The authors have adequately addressed my previous comments, and I can now recommend the manuscript for publication.

Author's Response to Peer Review Comments:

Heather J. Kulik **Massachusetts Institute of Technology**
Associate Professor of Chemical Engineering and Chemistry 77 Massachusetts Avenue, Building 66-464
Department of Chemical Engineering Cambridge, Massachusetts 02139

Department of Chemistry

PhoneEmail (617) 253hjkulik@mit.edu-
4584

June 25, 2025

Dear Editor,

Please find enclosed the resubmission of our paper entitled "High-Throughput Discovery of Ferrocene Mechanophores with Enhanced Reactivity and Network Toughening" for consideration of publication in *ACS Central Science*. The corresponding author is myself – contact information is given in the letterhead. All authors have seen and approved the submission of this manuscript, and it is being submitted exclusively to the *ACS Central Science*.

We have made changes according to the minor concerns raised by the reviewers, as indicated below. Reviewer comments are provided in full on pages 1-2 of this letter with response to each comment labeled “author reply” and reproduction of corresponding changes to the text with associated page numbers. We have also provided a copy of the original manuscript with all changes marked in red.

Sincerely,

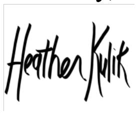

Heather J. Kulik

Reviewer #2:

- Please check all the inserted (highlighted) statements for English grammar. Examples are
"Given that lower SASA means Cp ring has lower solvent-accessible surface area, when the maximum SASA among two ligands is low,..."
"The selective scission of the crosslinker effectively increases the length of the highly tensioned strand, when now extends to the next crosslinker in sequence along the primary chain within the network."

Response: We thank the reviewer for their time and careful consideration of our work. We have reviewed the inserted statements for grammar.

Changes to text: Changed on page 12, in red: " Given that the lower SASA indicates lower solvent-accessible surface area for the Cp ring, a low maximum SASA for both ligands suggests that both ligands are sterically congested."

Changed on page 21, in red: "The selective scission of the crosslinker effectively increases the length of the highly tensioned strand, which now extends to the next crosslinker in sequence along the primary chain within the network."

- Figure 6: I thank the authors for the added ALMO-EDA analysis and added pictures to subfigure a. Consider first mentioning all text regarding subfigure an and then mentioning subfigure b. Right now the authors jump back and forth in the caption.

[redacted]

Also, "a" and "b" should be bold face.

Response: We have updated the caption to Figure 6, including reordering elements and making "a" and "b" boldface.

Changes to text: Reordered Figure 6 caption to read: " **Figure 6.** Force-modified ferrocene ligand dissociation of
NCI complexes with steric bulk. **a**, Calculated ligand dissociation barriers of **NUSZEG** and m-TMS-Fc and oTMS-FC complexes as a function of force. Linear fits for each curve are shown as black lines and their equations are shown on the top. For reference, the linear fit of unsubstituted ferrocene is shown as the dashed gray line. The threshold energy of 16.6 kcal/mol that can be used to estimate activation forces measured by single molecule force spectroscopy is shown as the bolded black line. Schematic representations of relevant complexes are shown in the middle. Atoms that had forces applied to them are highlighted as blue spheres. **b**, Density difference plots of the full complex, with dissociating ligand and

the rest of the complex in the ground and transition states at 1 nN external force, where red corresponds to negative density and blue corresponds to positive density."

- Figure 7: The caption should define "ring opening (Phi)" to make the figure accessible without searching in the text. Also the text uses small phi whereas the figure uses capital Phi.

Response: We have updated the caption and figure.
Changes to text: Figure 7 has been updated to read "where the amount of *g*DCC ring opening is expressed as φ, the fraction of *g*DCC mechanophores that have reacted."
